# Development and validation of an interpretable 3 day intensive care unit readmission prediction model using explainable boosting machines

**DOI:** 10.3389/fmed.2022.960296

**Published:** 2022-08-23

**Authors:** Stefan Hegselmann, Christian Ertmer, Thomas Volkert, Antje Gottschalk, Martin Dugas, Julian Varghese

**Affiliations:** ^1^Institute of Medical Informatics, University of Münster, Münster, Germany; ^2^Department of Anesthesiology, Intensive Care and Pain Medicine, University Hospital Münster, Münster, Germany; ^3^Institute of Medical Informatics, Heidelberg University Hospital, Heidelberg, Germany

**Keywords:** intensive care unit, readmission, artificial intelligence, machine learning, explainable AI, interpretable machine learning, doctor-in-the-loop, human evaluation

## Abstract

**Background:**

Intensive care unit (ICU) readmissions are associated with mortality and poor outcomes. To improve discharge decisions, machine learning (ML) could help to identify patients at risk of ICU readmission. However, as many models are black boxes, dangerous properties may remain unnoticed. Widely used *post hoc* explanation methods also have inherent limitations. Few studies are evaluating inherently interpretable ML models for health care and involve clinicians in inspecting the trained model.

**Methods:**

An inherently interpretable model for the prediction of 3 day ICU readmission was developed. We used explainable boosting machines that learn modular risk functions and which have already been shown to be suitable for the health care domain. We created a retrospective cohort of 15,589 ICU stays and 169 variables collected between 2006 and 2019 from the University Hospital Münster. A team of physicians inspected the model, checked the plausibility of each risk function, and removed problematic ones. We collected qualitative feedback during this process and analyzed the reasons for removing risk functions. The performance of the final explainable boosting machine was compared with a validated clinical score and three commonly used ML models. External validation was performed on the widely used Medical Information Mart for Intensive Care version IV database.

**Results:**

The developed explainable boosting machine used 67 features and showed an area under the precision-recall curve of 0.119 ± 0.020 and an area under the receiver operating characteristic curve of 0.680 ± 0.025. It performed on par with state-of-the-art gradient boosting machines (0.123 ± 0.016, 0.665 ± 0.036) and outperformed the Simplified Acute Physiology Score II (0.084 ± 0.025, 0.607 ± 0.019), logistic regression (0.092 ± 0.026, 0.587 ± 0.016), and recurrent neural networks (0.095 ± 0.008, 0.594 ± 0.027). External validation confirmed that explainable boosting machines (0.221 ± 0.023, 0.760 ± 0.010) performed similarly to gradient boosting machines (0.232 ± 0.029, 0.772 ± 0.018). Evaluation of the model inspection showed that explainable boosting machines can be useful to detect and remove problematic risk functions.

**Conclusions:**

We developed an inherently interpretable ML model for 3 day ICU readmission prediction that reached the state-of-the-art performance of black box models. Our results suggest that for low- to medium-dimensional datasets that are common in health care, it is feasible to develop ML models that allow a high level of human control without sacrificing performance.

## Introduction

Discharge decisions in an intensive care unit (ICU) are complex and require consideration of several aspects (1). Discharging a patient too early can lead to the deterioration of the patient's health status that requires subsequent ICU readmission. This is associated with mortality and poor outcomes such as an increased length of ICU stay (2–4). A study conducted in 105 ICUs in the United States in 2013 found a median ICU readmission rate of 5.9% (5). Identified risk factors include admission origin, comorbidities, physiological abnormalities, and age (4, 6, 7). However, incorporating all available information appropriately for interpretation of an individual patient case can be challenging for clinicians (8).

Machine learning (ML) can automatically detect patterns in large quantities of data and has already shown the potential to transform health care (9). However, many ML models are considered black boxes, since they can be too complex for humans to understand (10). Studies have found that ML models contained an unnoticed racial bias (11) or relied on dangerous correlations (12), which can cause distrust among stakeholders, preventing their adoption (13). Interpretable ML could alleviate these issues by providing human-understandable explanations, enabling users to ensure properties such as fairness or robustness (14). Many studies have used so-called *post hoc* explanation methods such as local interpretable model-agnostic explanations (15) or Shapley additive explanations (16), which provide an explanation for a single prediction (17–19). However, *post hoc* methods have several shortcomings with respect to robustness and adversarial attacks (20–22) limiting their usefulness in health care settings (23). Hence, in this work, we used inherently interpretable or transparent models (10, 24) that allow humans to inspect and understand the entire model before using it for predictions.

A research gap exists owing to the lack of studies about transparent ML models for health care that include human evaluations. A recent review on explainable artificial intelligence using electronic health records showed that only nine out of 42 studies used inherently interpretable models (25). Applications included mortality prediction, disease classification, risk stratification, and biomedical knowledge discovery. However, only three studies reported human expert confirmation of their results, which is considered essential for a meaningful evaluation of interpretable ML (14). For ICU readmission prediction, we identified two papers (26, 27) that explicitly developed interpretable models based on rule sets and logistic regression (LR). However, no human validation of the results was performed.

In this study, we aimed to develop an inherently interpretable explainable boosting machine (EBM) model for the prediction of 3 day ICU readmission. We involved clinicians in the development process to inspect and verify the entire model. The validation process was evaluated to determine its effect and reveal possible issues. Second, the resulting EBM model was compared with different baseline and state-of-the-art black box ML models to assess the effect of transparency on performance.

## Materials and methods

### Study setting and preregistration

**Figure 1:**
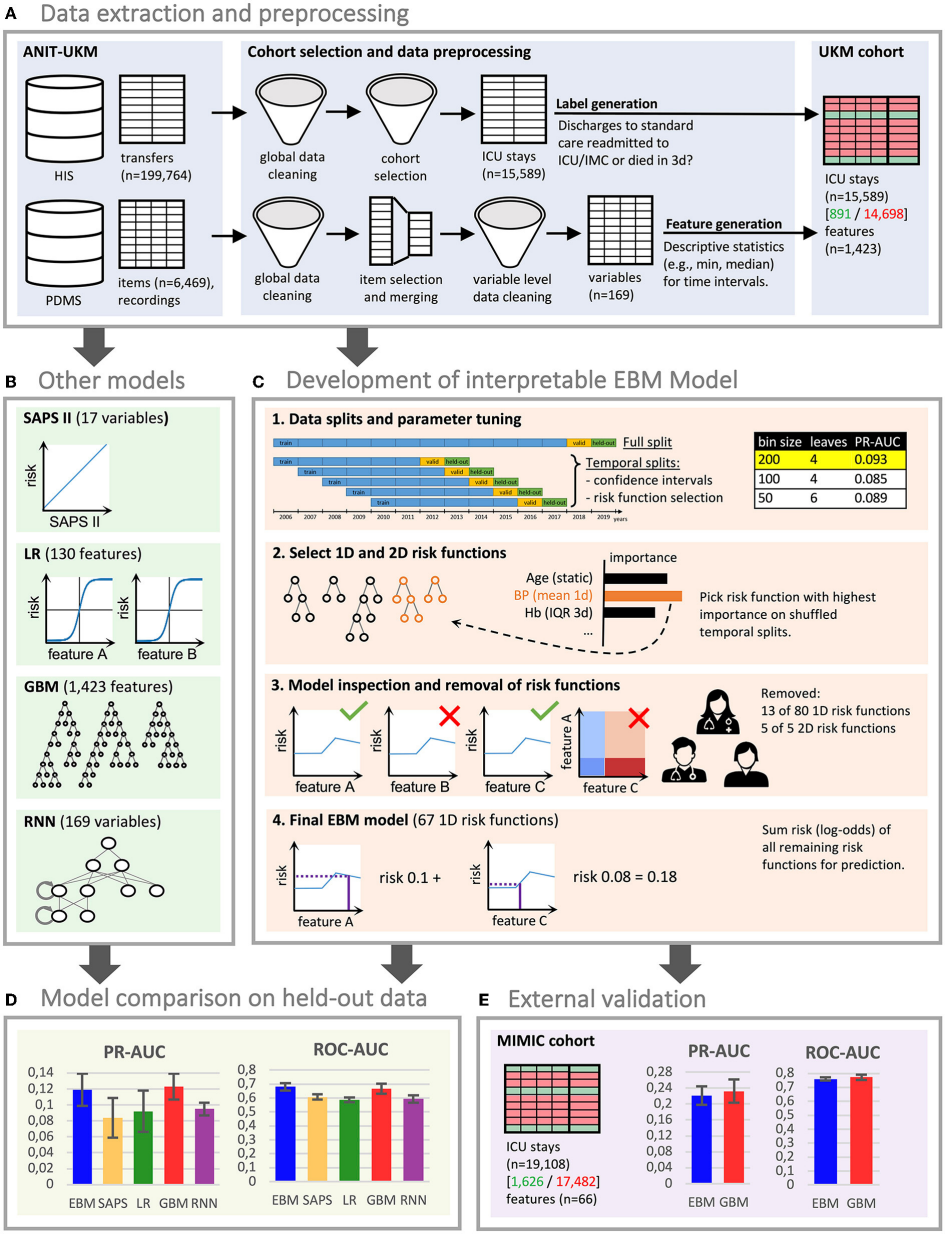
Flowchart of the study. **(A)** We created a local cohort for the development of machine learning (ML) models. Information on intensive care unit (ICU) transfers was extracted from the hospital information system (HIS), and ICU data was extracted from the patient data management system (PDMS). Extensive preprocessing was applied to clean the data. We generated labels for 3 day ICU readmission and descriptive statistics as features. **(B)** Four ML models were developed for comparison. For LR, we also performed feature selection. The RNN directly uses the time series data. **(C)** The development of the EBM model involved four steps [see 1–4 in **(C)**]. We conducted parameter tuning for EBM (and our other models) and performed greedy risk function selection based on the importance determined on the temporal splits. In step 3, we inspected the model with a team of clinicians to identify and remove problematic risk functions. The remaining risk functions were used for the predictions. **(D)** We evaluated all models for their area under the precision-recall curve (PR-AUC) and area under the receiver operating characteristic curve (ROC-AUC) on the hold-out split. **(E)** External validation for the EBM and GBM models was performed on the Medical Information Mart for Intensive Care (MIMIC) version IV. **(D,E)** Error bars were determined with the standard deviation on five temporal splits. EBM, explainable boosting machine; SAPS II, Simplified Acute Physiology Score II; LR, logistic regression; GBM, gradient boosting machine; RNN, recurrent neural network.

This study was approved by the ethics review board of the medical chamber Westfalen-Lippe (reference number: 2020-526-f-S). We provided the TRIPOD (Transparent Reporting of a Multivariable Prediction Model for Individual Prognosis or Diagnosis) checklist (28) in Supplementary material 1. This work was preregistered online (29); however, it had two deviations: a readmission interval of 3 days instead of 7 days was considered to exclude fewer patients with insufficient follow-ups. Also, we only performed external validation for the final performance results, which we considered most relevant. An overview of all steps conducted for this study can be found in Figure 1. All code for preprocessing the data, training the models, and inspecting the final EBM model is publicly available (30, 31).

### Cohort

**Figure 2:**
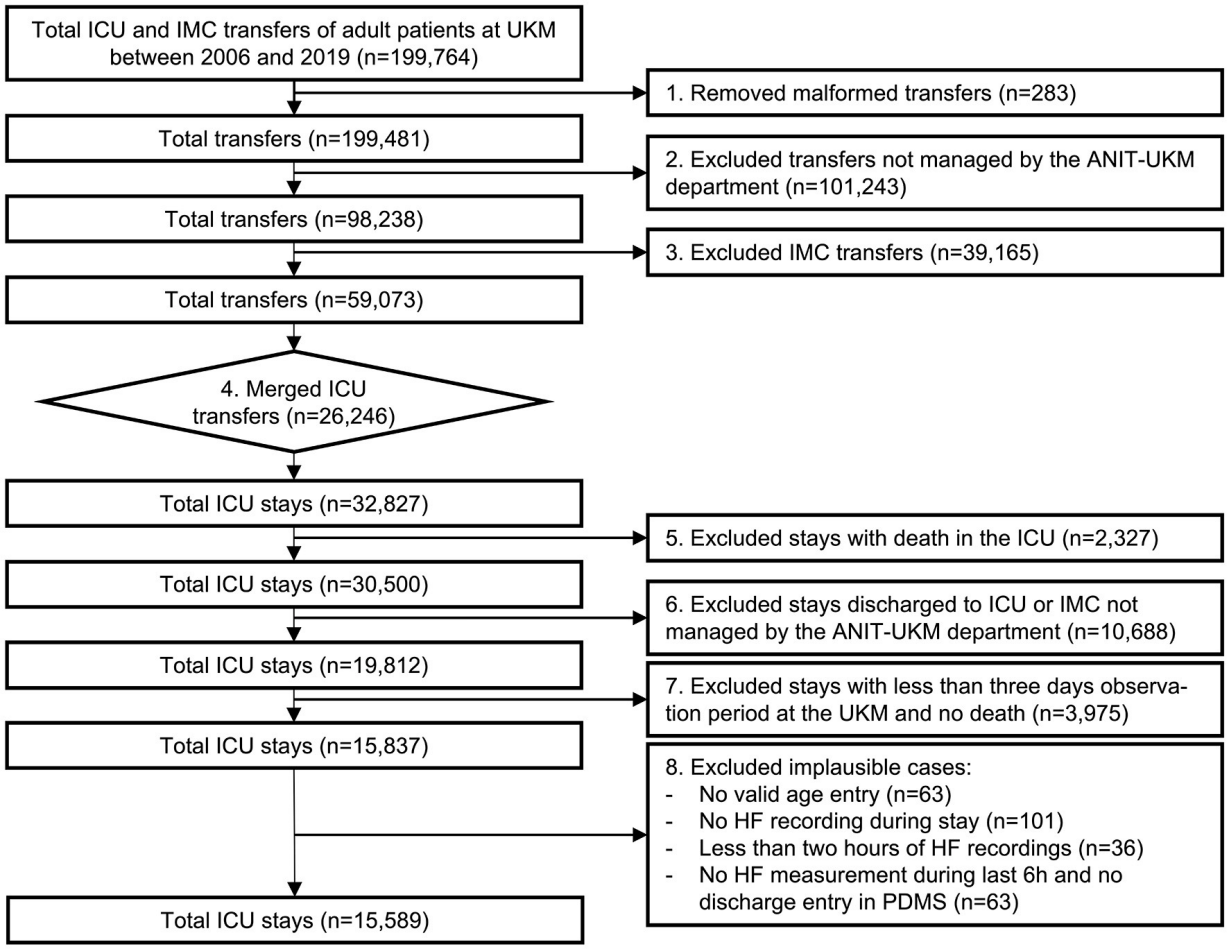
Flowchart of the cohort selection for the University Hospital Münster (UKM) cohort. Transfers to ICU and IMC wards of the UKM between 2006 and 2019 served as initial data. We included four ICUs managed by the ANIT-UKM department. Transfers had to be merged using a manual procedure to obtain consecutive ICU stays. Patients who died in the ICU and those who were discharged to an external ICU or IMC were excluded. We required an observation period of at least 3 days to ensure readmission to an ICU in the UKM. Lastly, implausible cases were removed.

We included all ICU patients managed by the Department of Anesthesiology, Intensive Care and Pain Medicine at the University Hospital Münster (ANIT-UKM) who were discharged to standard care and had a follow-up period of at least 3 days (see Figure 2). Initially, all ICU and intermediate care (IMC) transfers of adult patients between 2006 and 2019 were retrieved from the hospital information system (HIS; ORBIS, Dedalus Healthcare Group; *n* = 199,764). First, 283 entries were removed because of ambiguous discharge dates, overlapping hospital stays, or overlapping transfers that could not be delineated. Next, transfers not managed by the ANIT-UKM (*n* = 101,243) and IMC transfers (*n* = 39,165) were excluded. In step 4, we merged consecutive transfers (*n* = 26,246) into a single ICU stay. Some entries (*n* = 147) contained artifacts with short intervals between two transfers, and we designed a stepwise procedure to decide whether a discharge occurred. Next, we excluded ICU stays that ended with the death of the patient (*n* = 2,327) or a discharge to an external ICU or IMC unit (*n* = 10,688). We used the same procedure as in step 4 to identify artifacts (*n* = 67) and to distinguish consecutive transfers and readmissions to an external ICU. We then excluded all ICU stays without a 3 day follow-up period at the UKM to ensure that all patients with worsening conditions who were included were transferred to an observed ICU (*n* = 3,975). This also excluded patients who were transferred to an external facility or home, which introduced a selection bias. However, we reckoned that ensuring a complete observation interval outweighed this effect. Lastly, we removed implausible cases with no age entry (*n* = 63) or that had only very few heart frequency recordings (*n* = 200); thus, 15,589 ICU stays were included.

ICU patients who were readmitted to any ICU (*n* = 822) or IMC unit (*n* = 31) or died within 3 days (*n* = 38) were labeled as true (Supplementary material 2). Patient deaths were also labeled to obtain a consistent outcome. Patients who were discharged to standard care and underwent a planned procedure with a subsequent re-admission to an ICU or IMC unit incorrectly received a positive label. However, we considered this effect to be small. To verify our cohort selection and labeling procedure, we sampled 20 positive stays stratified across wards and verified them using additional clinical information.

Table 1 summarizes the key characteristics of the resulting UKM cohort. The ICU patients of the included stays had a mean age of 63.33 ± 14.73 years, and more than two-thirds of them were male (*n* = 10,670). ICU patients with 3 day readmission or who died after discharge showed several differences: the patients were 3 years older on average, the proportion of male patients further increased from 68.3 to 70.8%, and the mean length of the previous ICU stay was approximately 13.5 hours longer. Supplementary material 2 contains an overview of the included ICUs.

**Table 1:** Overview of the UKM cohort.

**Characteristic**	**All ICU stays**	**No 3 day readmission or death after ICU discharge**	**3 day readmissions or death after ICU discharge**
Number of ICU stays, *n* (%)	15,589 (100.0)	14,698 (94.3)	891 (5.7)
Number of patients, *n* (%)	14,188 (100.0)	13,349 (94.1)	839 (5.9)
Age, mean ± SD, years	63.33 ± 14.73	63.16 ± 14.77	66.08 ± 13.85
Female sex, *n* (%)	4,919 (100.0)	4,659 (94.7)	260 (5.3)
Male sex, *n* (%)	10,670 (100.0)	10,039 (94.1)	631 (5.9)
Length of ICU stay, mean ± SD, days	3.70 ± 8.08	3.67 ± 8.11	4.23 ±7.53
ICU at discharge	ICU 1 (*n* = 4,063)	ICU 1 (*n* = 3,820)	ICU 1 (*n* = 243)
	ICU 2 (*n* = 6,402)	ICU 2 (*n* = 6,035)	ICU 2 (*n* = 367)
	ICU 3 (*n* = 1,034)	ICU 3 (*n* = 960)	ICU 3 (*n* = 74)
	ICU 4 (*n* = 4,090)	ICU 4 (*n* = 3,883)	ICU 4 (*n* = 207)

The key characteristics of all included ICU stays and the ICU stays divided by their labels. This information is based on ICU stays, so a single patient can be considered more than once.

### Variables and features

We included data that was routinely collected in the ICU for our analysis. For this purpose, 6,496 item definitions with 651,258,647 time-stamped recordings were extracted from the patient data management system (PDMS; Quantitative Sentinel, GE Healthcare) of the ANIT-UKM (see the flow chart in Supplementary material 2). We excluded all variables that were not collected during the study period (*n* = 1,322), derived variables computed using formulas in the PDMS (*n* = 1,029), and clinical notes because of the highly heterogeneous data quality (*n* = 777). We also excluded clinically irrelevant variables (*n* = 1,979) such as device-specific or billing information. The remaining 1,362 variables were processed in consultation with a senior physician who had extensive experience with the PDMS. For 802 non-medication variables, we determined the coverage across the study period and generated descriptive statistics to exclude irrelevant variables (*n* = 522). Of the resulting 280 variables, 70 were included directly, and 210 were further processed and merged into 50 variables. For medications, we assigned World Health Organization Anatomical Therapeutic Chemical (ATC) codes to all entries. We defined 44 clinically relevant medication categories within the ATC hierarchy and merged the respective variables. All medication variables that were not assigned to any category were excluded (*n* = 187). In addition, we manually determined five medication categories as additional variables for therapeutic and prophylactic antithrombotic agents and equivalence dosages of cardiac stimulants, norepinephrine and dopamine, and glucocorticoids, which we considered clinically relevant. Hence, we included 120 non-medication and 49 medication variables (Supplementary material 2). Further data cleaning methods are described in Supplementary material 2.

We assigned variables to nine different classes according to their data and generated respective features for each class (see Supplementary material 2). This was particularly important for time series data since EBM models cannot handle it. We featurized time series data *via* median, interquartile range (IQR), minimum, maximum, and linear trend for different time windows. We defined three time horizons (high, medium, and low) based on the median sampling interval of a variable that used different time windows before ICU discharge (high: 4, 12, and 24 hours; medium: 12, 24 hours, and 3 days; low: 1, 3, and 7 days). Hence, we generated 15 features for each time series variable. Patient flows, medications, and interventions were always considered as low time horizon. For patient flows, we extrapolated the daily flow. For medications, we used a binary indicator and the number of administered drugs. For interventions, we also used a binary indicator and the interval since it was last performed. For static data, we used the last value from the most appropriate time interval (patient history, hospital stay, and ICU stay). Four additional features were created manually, which results in a total of 1,423 features. A list of all variables, feature classes, and their respective features is given in Supplementary material 2.

### Explainable boosting machines and baseline models

EBMs belong to the class of generalized additive models (32). A generalized additive model (33) models a label ŷ by a bias term β_0_ and a sum of features transformed by shape functions *f*_*i*_(*x*_*i*_). The label ŷ can optionally be transformed by a link function *g* (see equation 1). EBMs add additional shape functions for the interactions of two variables *f*_*i, j*_(*x*_*i*_, *x*_*j*_) (34) and use the logit link function for dichotomous classifications analogous to LR (see equation 2, note that the logit function was moved to the right side).


(1) $$\begin{array}{r} g\left(ŷ\right)=\beta_{0}+\underset{i}{\sum }f_{i}\left(x_{i}\right) \end{array}$$

(2) $$\begin{array}{r} ŷ=logit^{-1}\left(\beta_{0}+\underset{i}{\sum }f_{i}\left(x_{i}\right)+\underset{i\neq j}{\sum }f_{i,j}\left(x_{i},\;x_{j}\right)\right) \end{array}$$

In this study, the shape functions *f*_*i*_(*x*_*i*_) and *f*_*i, j*_(*x*_*i*_, *x*_*j*_) of EBMs are also called one- (1D) and two-dimensional (2D) risk functions, because each of them models the log-odds of being readmitted to the ICU within 3 days. Different methods can be used to estimate the risk functions (33). EBMs use boosted decision trees that allow versatile function shapes that have shown optimal performance across several tasks (35). By visualizing the learned risk functions, EBMs can be inspected and owing to their modularity, inappropriate functions can be removed. Also, for a given input, contributions of each risk function can be used as an explanation of a prediction. A study that applied them in two health care tasks highlighted their potential to identify and remove spurious correlations (12). Moreover, an evaluation revealed that physicians can grasp the concept of EBMs and feel confident working with them (36). In this work, we compared to the validated Simplified Acute Physiology Score (SAPS) II, LR with feature selection, gradient boosting machines (GBMs), and recurrent neural networks (RNNs) with long short-term memory units for comparison (Supplementary material 2). We selected 130 features for the LR model, and we conjectured that inspecting this model requires a similar effort as inspecting our EBM model with at most 100 risk functions. Hence, the LR model serves as an interpretable baseline of the same complexity. GBMs and RNNs are both considered black box models owing to their complexity.

### Development of the EBM model with a limited number of risk functions

For our experiments, we used the area under the precision-recall curve (PR-AUC) as the primary performance indicator due to the label imbalance. We also reported the area under the receiver operating characteristic curve (ROC-AUC) since it is commonly reported in the medical literature. We selected the two most recent years for validation and hold-out data to simulate a real-world deployment (17). Five temporal splits were used for risk function selection and estimation of the standard deviation as pseudo-confidence intervals (Supplementary material 2).

To limit the model size and allow inspection in a reasonable amount of time, we performed automatic risk function selection of at most 80 1D and 20 2D functions based on their importance. To obtain good parameters, we first performed tuning based on the PR-AUC on the train and validation data of the full split (Supplementary material 2). We did this in three steps: we performed parameter tuning on all features, we estimated the 80 most important 1D risk functions approximately, and performed another parameter tuning for these 80 risk functions. Next, we used these parameters for risk function selection in a greedy stepwise forward procedure based on their mean importance on the five temporal splits (Supplementary material 2). We used the temporal splits to get more robust estimates and to prevent overfitting on the full split. A random 85% training and 15% validation split were used for each temporal split because a subset of variables was only collected for some years, which led to a biased weight estimate when using training and validation data based on years. Importance was calculated as the mean absolute log-odds score of a risk function. Finally, we chose the risk function selection with the highest PR-AUC performance on the full validation split. We repeated the same procedure for 2D risk functions on the features of the included 1D risk functions. This is coherent with the EBMs training algorithm, which first trains 1D functions and then adds 2D functions for the residuals.

### Inspection of the EBM model by a multidisciplinary team

The goal of the EBM model inspection was to identify the risk functions that should not remain in the final prediction model. The model was inspected by a team of three individuals: a senior physician working at the included ICUs, a senior physician responsible for the data infrastructure at the ANIT-UKM, and the developer of the EBM model with a machine learning and health care background. They discussed and determined potential problems of the risk functions a priori to agree on a common set of exclusion criteria. For each risk function, they discussed its main properties and agreed on its content, then they determined if any of the identified problems applied, and then they decided if the problems justified the exclusion of a risk function. We recorded the identified problems for all risk functions (Supplementary material 3) and collected qualitative feedback during the EBM model inspection (Supplementary material 2).

### External validation on the medical information mart for intensive care version IV database

We used the Medical Information Mart for Intensive Care (MIMIC) version IV database for external validation (37, 38). It contains 76,540 ICU stays of 53,150 patients admitted to the Beth Israel Deaconess Medical Center between 2008 and 2019. After applying a similar cohort selection and labeling procedures, we included 19,108 ICU stays, of which 1,626 (8.5%) were labeled positively (Supplementary material 2). For performance comparison, we resampled negative instances to obtain the same positive rate as in the UKM cohort. We extracted 41 variables responsible for the 67 features used in the final EBM model from MIMIC-IV. Only a single variable could not be created. We also performed external validation with the GBM model, as it performed best in the model comparison. However, we only used the variables of the EBM model because extracting all variables from the MIMIC-IV database was not feasible. Both models were trained again on the MIMIC-IV data.

## Results

### Development of the EBM model with a limited number of risk functions

We first performed parameter tuning for an EBM with all features (Supplementary material 2). The best EBM with 1,423 1D risk functions achieved a PR-AUC of 0.151 ± 0.028 and a ROC-AUC of 0.652 ± 0.034 on the hold-out split. Next, we performed risk function selection based on the five temporal splits. Supplementary material 2 contains the performance for different numbers of risk functions and bin sizes. The best EBM model had a bin size of 200 and contained 80 1D risk functions. It achieved a PR-AUC of 0.130 ± 0.021 and a ROC-AUC of 0.681 ± 0.026. We repeated the same procedure for the 2D risk functions. We added five 2D functions with a bin size of four. The resulting model showed a decreased performance, with a PR-AUC of 0.113 ± 0.018 and ROC-AUC of 0.646 ± 0.01. The 85 most important risk functions of the resulting EBM model and their respective variables, features, and relative importance (variance) are listed in Table 2. The five 2D risk functions yielded the highest importance, followed by the 1D functions for endotracheal tube, age, antithrombotic agents in a prophylactic dosage, partial thromboplastin time, and O_2_ saturation. The graphical representations of all risk functions are given in Supplementary material 3.

**Table 2:** Overview of the variables and features of the risk functions included in the final EBM model ordered by importance.

**No**.	**Variable(s)**	**Feature(s)**	**Relative importance %**	**Excluded during model inspection**
1	Age [years], Base Excess (BE) [mmol/L]	Static per patient, IQR 3 days	4.20	X
2	Drugs for constipation, Leucocytes [thousand/μL]	Unique 1 day, median 1 day	3.52	X
3	Blood volume out [mL], Procalcitonin [ng/mL]	Extrapolate 7 days, maximum 7 days	2.57	X
4	Hematocrit [%], Blood volume out [mL]	Maximum 3 days, extrapolate 3 days	2.19	X
5	Leucocytes [thousand/μL], Blood volume out [mL]	Median 1 day, extrapolate 3 days	1.87	X
6	Endotracheal tube (tubus) exists	Days since last application	1.71	
7	Age [years]	Static per patient	1.70	
8	Antithrombotic agents prophylactic dosage	Days since last application	1.65	
9	Partial thromboplastin time (PTT) [s]	Maximum 1 day	1.63	X
10	O_2_ saturation [%]	Minimum 12 hours	1.58	
11	Blood volume out [mL]	Extrapolate 7 days	1.52	
12	Gamma-GT [U/L]	Median 7 days	1.46	
13	Chloride [mmol/L]	Trend per day 3 days	1.40	
14	Heart rate [bpm]	Minimum 4 hours	1.39	
15	Partial thromboplastin time (PTT) [s]	Maximum 3 days	1.37	X
16	Chloride [mmol/L]	Minimum 1 day	1.37	
17	Hemoglobin [mmol/L]	Maximum 3 days	1.30	
18	Length of stay before ICU [days]	Manually added	1.28	
19	Hematocrit [%]	Maximum 3 days	1.26	
20	Calcium [mmol/L]	Trend per day 3 days	1.26	X
21	Estimated glomerular filtration rate (eGFR) ml/min/1.73 m^2^	Trend per day 7 days	1.24	
22	Richmond agitation sedation (RAS) scale	Maximum 3 days	1.24	
23	Urine volume out [mL]	Extrapolate 1 day	1.24	
24	Thrombocytes [thousand/μL]	Trend per day 7 days	1.24	
25	Blood volume out [mL]	Extrapolate 3 days	1.23	
26	paO_2_/FiO_2_ [mmHg/FiO_2_]	Median 1 day	1.21	
27	pH	Trend per day 3 days	1.21	
28	Phosphate [mg/dL]	Minimum 7 days	1.20	
29	pH	Median 1 day	1.20	
30	Body core temperature [°C]	Minimum 1 day	1.18	X
31	Creatine kinase (CK) [U/L]	Minimum 7 days	1.15	
32	Richmond agitation sedation (RAS) scale	Trend per day 12 hours	1.13	X
33	Potassium [mmol/L]	Median 1 day	1.13	
34	Glasgow coma scale (GCS) score	Minimum 3 days	1.11	
35	Body core temperature [°C]	Median 1 day	1.10	
36	Base excess (BE) [mmol/L]	IQR 3 days	1.10	X
37	Blood urea nitrogen [mg/dL]	Minimum 3 days	1.10	
38	paO_2_/FiO_2_ [mmHg/FiO_2_]	Trend per day 3 days	1.09	
39	Drugs for constipation	Unique 1 day	1.09	
40	Urine volume out [mL]	Extrapolate 7 days	1.09	
41	Partial thromboplastin time (PTT) [s]	Minimum 7 days	1.07	X
42	Diastolic blood pressure [mmHg]	Median 1 day	1.06	
43	Partial pressure of oxygen (pO_2_) [mmHg]	Minimum 12 hours	1.06	
44	Creatine kinase-MB (CK-MB) [U/L]	Maximum 3 days	1.05	
45	Richmond agitation sedation (RAS) scale	Maximum 1 day	1.05	
46	Partial thromboplastin time (PTT) [s]	Minimum 3 days	1.05	X
47	Systolic blood pressure [mmHg]	IQR 12 hours	1.05	
48	paO_2_/FiO_2_ [mmHg/FiO_2_]	Median 3 days	1.04	
49	Creatine kinase (CK) [U/L]	Median 7 days	1.04	X
50	Lactate [mmol/L]	Maximum 3 days	1.04	
51	Creatine kinase-MB (CK-MB) [U/L]	Median 3 days	1.04	
52	Lactate [mmol/L]	Minimum hours	1.00	
53	Phosphate [mg/dL]	Maximum 1 day	1.00	
54	Partial thromboplastin time (PTT) [s]	Maximum 7 days	0.98	X
55	Partial pressure of carbon dioxide (PCO_2_) [mmHg]	Median 1 day	0.98	
56	Base excess (BE) [mmol/L]	Trend per day 3 days	0.97	
57	Glucose [mg/dL]	Median 3 days	0.97	
58	Base excess (BE) [mmol/L]	Minimum hours	0.96	
59	Methemoglobinemia (MetHb) [%]	Minimum hours	0.96	
60	Is on automatic ventilation	Days since last application	0.95	
61	Body core temperature [°C]	Minimum 4 hours	0.95	X
62	Partial pressure of carbon dioxide (PCO_2_) [mmHg]	IQR 1 day	0.95	
63	Sodium [mmol/L]	Median 3 days	0.93	
64	Leucocytes [thousand/μL]	Median 1 day	0.92	
65	Sodium [mmol/L]	Trend per day 3 days	0.92	
66	Procalcitonin [ng/mL]	Maximum 7 days	0.91	
67	Base excess (BE) [mmol/L]	Median hours	0.91	
68	Mean blood pressure [mmHg]	Median 4 hours	0.87	
69	Leucocytes [thousand/μL]	Trend per day 3 days	0.84	X
70	pH	Median 3 days	0.84	
71	Bilirubin total [mg/dL]	Maximum 7 days	0.84	
72	Partial pressure of oxygen (pO_2_) [mmHg]	IQR hours	0.84	
73	Base excess (BE) [mmol/L]	IQR 1 day	0.83	
74	Body core temperature [°C]	Trend per day 1 day	0.83	
75	C-reactive protein [mg/dL]	Maximum 3 days	0.83	
76	Heart rate [bpm]	Minimum 1 day	0.82	
77	Hematocrit [%]	Median hours	0.80	
78	Partial pressure of carbon dioxide (PCO_2_) [mmHg]	Minimum 3 days	0.76	
79	Mean blood pressure [mmHg]	Median hours	0.72	
80	Calcium [mmol/L]	Maximum 1 day	0.69	
81	Estimated respiratory rate	Median 1 day	0.68	
82	pH	IQR 1 day	0.67	
83	Leucocytes [thousand/μL]	IQR 3 days	0.63	
84	Heart rate [bpm]	IQR 4 hours	0.60	
85	Reduced hemoglobin (RHb)	Median hours	0.60	X

These risk functions were selected from a total of 1,423 based on their importance on the five-temporal splits. Risk functions 1–5 are two-dimensional, and the remaining functions are one-dimensional. The relative importance was determined on the final training split. The last column indicates whether a risk function was excluded during the model inspection by a team of physicians. Visualizations of all risk functions and the detailed reasons for exclusion are given in the supplement.

### Inspection of the EBM model by a multidisciplinary team

The resulting EBM model was inspected by a multidisciplinary team including two clinicians to identify and remove problematic risk functions. A priori to the model inspection, they identified four potential problems that they assigned to risk functions during the inspection:

- It encodes health care disparities that should not be reproduced (*n* = 0)
- It contains undesirable artifacts from the data generation process (*n* = 8)
- It contradicts medical knowledge (*n* = 13)
- It is not interpretable so that its effect cannot be clearly determined (*n* = 17).

**Figure 3:**
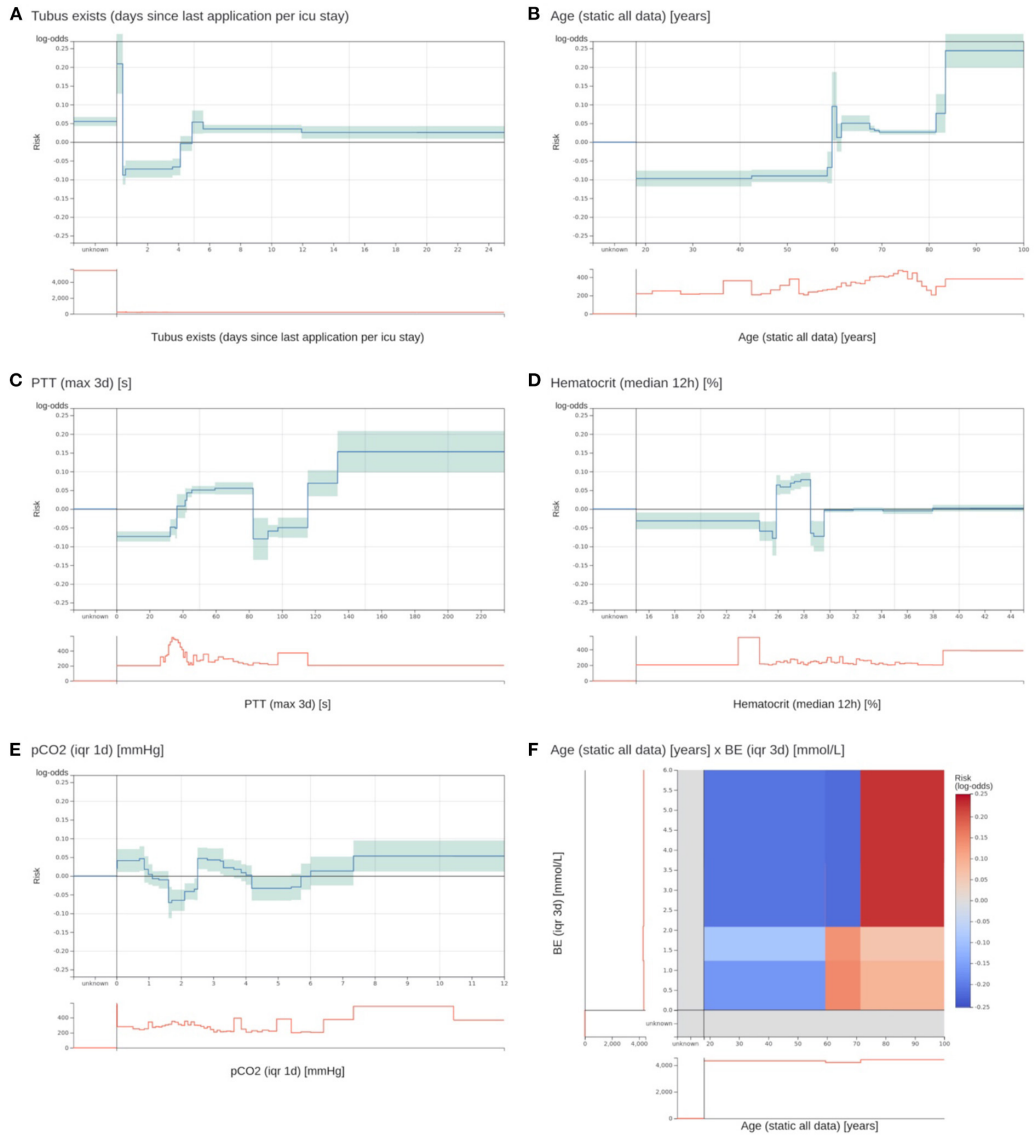
Two most important risk functions and four excluded risk functions of the EBM model. **(A,B)** Two most important risk functions that are included in the EBM model. **(A)** Contains the number of days since the last existence of an endotracheal tube. Patients that have an endotracheal tube immediately before discharge have a highly increased risk. Lower risk is assigned to values between 0.4 and 4.1 days. Also, patients with no endotracheal tube (unknown) receive an increased risk. **(B)** The risk function for age shows an increased risk for higher age values. There is a peak at 60 years with no obvious explanation. **(C)** A maximum PTT value over the last 3 days before discharge between 82.5 and 115.5 s gets a lower risk for 3 day ICU readmission. It was identified that this is an artifact of the previous procedure to determine the PTT for cardiac surgery patients. This will not generalize for future data. **(D)** For a median hematocrit between 24.875 and 28.525%, the model determined an elevated risk. For slightly lower and higher values, the risk is negative. This is against common medical knowledge, where a decreasing hematocrit value should be associated with increased risk. **(E)** The interquartile range (IQR) of the partial pressure of carbon dioxide (pCO2) over the last day before discharge receives an increased risk for values between 0 and 0.863 and 2.513 and 3.313 mmHg. However, the interpretation of this behavior and determining its clinical implications was impossible. **(F)** The 2D risk function for age and the IQR of the base excess (BE) over 3 days. Patients over 71.5 years have a high risk for a high IQR of the BE. Patients between 59.5 and 71.5 have only a slightly increased risk for low IQR values, and younger patients have a decreased risk across all BE values. The team excluded it due to a lack of interpretability.

The model inspection took 4 hours, that is, approximately 3 minutes per function. Not all risk functions with a problem were excluded, so we assigned the risk functions into three classes: included without problems (*n* = 52), included with problems (*n* = 15), and excluded with problems (*n* = 18). Most functions were excluded owing to the lack of interpretability (*n* = 10), followed by undesirable artifacts (*n* = 6) and contradictions of medical knowledge (*n* = 6). More than one problem could be assigned to each risk function. Five functions for partial thromboplastin time (PTT) were excluded because of artifacts. Using the feature histograms, the team recognized a change in the PTT measurement procedure since 2019, invalidating the risk functions learned on the training data. Also, all 2D risk functions were labeled as not interpretable and were excluded from the model. Figure 3 shows two included 1D risk functions and three 1D and one 2D functions that were excluded because of different problems. After model inspection, the EBM contained 67 1D risk functions. It achieved a PR-AUC of 0.119 ± 0.020 and a ROC-AUC of 0.680 ± 0.025 on the hold-out data. Hence, inspection decreased the PR-AUC and increased the ROC-AUC compared with a model trained on all 1D risk functions.

We collected qualitative feedback from the team during model inspection (Supplementary material 2). A major problem was drawing the line for risk function exclusion. Most functions partially fulfilled at least one problem. The team agreed to exclude a risk function when a problem was clearly present and would have a considerable impact on patients; that is, value ranges with many patients affected. Still, many functions could be assigned to either category (comments 1–3). The team stated that it was difficult to consider the cohort reduced to a single independent risk function (comments 4–7). This is against clinical practice, where several patient measurements are integrated. Also, only examining patient features at the time of discharge was hard, since usually the whole patient history is factored in (comment 8). In addition, the team members tended to construct explanations for risk functions without clear evidence (comment 9). Moreover, values outside the usual value ranges and IQR and trend features were more difficult to understand (comments 10 and 11). In particular, the 2D functions posed a problem because the combinations of features were uncommon in clinical practice. Even though it was possible to grasp the content of the risk function, it was difficult to infer its clinical implications that led to exclusion (comment 12). There was a tendency to rely more on the model to derive useful relationships when a risk function was less interpretable (comment 13). In addition to that, we collected general properties that hindered or supported interpretability, which confirmed previous findings (36).

### Performance of EBM compared to baseline models

**Figure 4:**
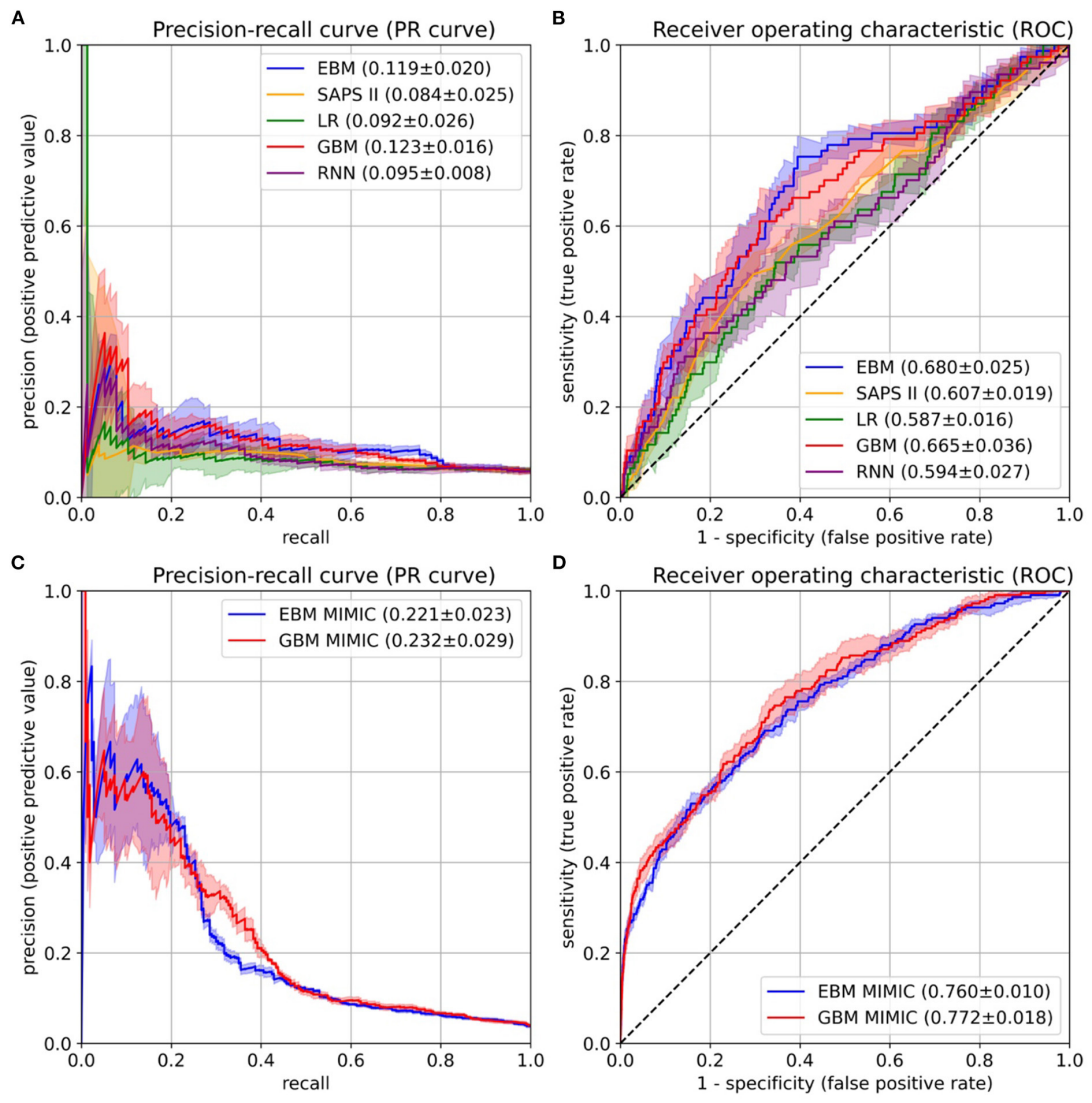
Performance evaluation on the University Hospital Münster (UKM) cohort **(A,B)** and external validation on the Medical Information Mart for Intensive Care version IV (MIMIC-IV) database **(C,D)**. **(A)** The area under the precision-recall curve (PR-AUC) was considered the most relevant performance indicator owing to the imbalanced label distribution. We optimized the PR-AUC during the parameter tuning and selection procedures for all models. The differences between models are relatively small. The explainable boosting machines (EBMs) and gradient boosting machines (GBMs) show the highest PR-AUC. **(B)** The area under the receiver operating characteristic curve (ROC-AUC) was determined as an additional performance measure. Again, the EBM and GBM models performed best. **(C,D)** The same performance indicators were determined on the MIMIC-IV database. Both models again showed similar results. The confidence intervals for all curves were determined with the standard deviation on the five temporal splits.

After the risk function selection and model inspection, the EBM model contained 67 1D risk functions. It achieved a PR-AUC of 0.119 ± 0.020 and a ROC-AUC of 0.680 ± 0.025 (Figure 4). For recall values of 0.4, 0.5, 0.6, and 0.8 the precision values were 0.130 ± 0.032, 0.111 ± 0.019, 0.105 ± 0.013, and 0.082 ± 0.005. Utilizing SAPS II in the last 24 hours showed an inferior performance of 0.084 ± 0.025 (PR-AUC) and 0.607 ± 0.019 (ROC-AUC). Also, LR with 130 selected features and the RNN achieved a lower performance, with a PR-AUC of 0.092 ± 0.026 and 0.095 ± 0.008 and a ROC-AUC of 0.587 ± 0.016 and 0.594 ± 0.027. Both were placed between the EBM and SAPS II for PR-AUC and below SAPS II for ROC-AUC. The latter could be due to the optimization of PR-AUC during parameter tuning and variable selection. The GBM trained on all 1,423 features achieved a PR-AUC of 0.123 ± 0.016 and a ROC-AUC of 0.665 ± 0.036. Hence, it performed similarly to the developed EBM model with 67 1D risk functions.

### External validation on the medical information mart for intensive care version IV database

The final EBM model for the UKM cohort used 67 features generated by 42 variables. We extracted 41 of those variables from MIMIC-IV. Variables were collected differently for the MIMIC cohort (Supplementary material 2). The EBM for external validation contained 66 1D risk functions. For the GBM model, we generated all the features of the 41 variables, resulting in 515 features. The EBM and GBM performed similarly on MIMIC-IV, with a PR-AUC of 0.221 ± 0.023 and 0.232 ± 0.029 and a ROC-AUC of 0.760 ± 0.010 and 0.772 ± 0.018 (Figure 4). This performance was much higher than that for the UKM cohort, which we mainly attributed to the better data quality of MIMIC-IV.

## Discussion

This study showed that for the prediction of 3 day ICU readmission, a transparent EBM model containing only 67 risk functions performed on par with state-of-the-art GBMs trained on 1,423 features and outperformed RNNs trained on time series data. Both the GBMs and RNNs can be considered black box models owing to their complexity. Hence, we found additional evidence that in a health care setting with structured data, a simple and inherently interpretable model can be sufficient for competitive prediction performance (10). The final model achieved a PR-AUC of 0.119 ± 0.020 and a ROC-AUC of 0.680 ± 0.025. External validation on the MIMIC-IV database showed improved EBM results of a PR-AUC of 0.221 ± 0.023 and a ROC-AUC of 0.760 ± 0.010 and confirmed that they performed similarly to the GBMs. Our results are consistent with those of previous studies, showing that EBMs outperformed LR and were on par with random forests and boosting methods (12, 34). However, in contrast to the existing work, adding 2D risk functions lead to lower performance on the hold-out data. Several risk functions of the final EBM model are consistent with the main risk factors reported in the literature (4, 6, 7), such as age, length of hospital stay before ICU admission, disease severity (e.g., based on the GCS score), physiological state (e.g., heart rate), and need for organ support (e.g., presence of an endotracheal tube). In our study, many concepts had much finer granularity; for example, several variables captured the physiological state of the patient. We also note that some known risk factors were available features but did not end up in the final model. Among those are sex, admission origin, and use of vasopressors. However, some information might be mediated through other variables. For example, blood loss is usually a clear indicator of a past surgery and might contain additional information, making it more relevant than a simple indicator for surgery. The overall predictive performance for 3 day ICU readmissions was relatively low. This is probably due to the limitations regarding data quality, which are supported by the higher performance on MIMIC-IV. MIMIC-IV was created in several iterations and integrated the feedback of many researchers, which led to higher data quality. Moreover, the prediction of ICU readmission prediction is a difficult task, and only a few readmissions are preventable (39). Still, we think that an EBM model for the prediction of 3 day ICU trained on a local cohort can offer useful insights for decision-making in the ICU.

Several studies on ICU readmission prediction have been conducted (26, 40–52), and we identified two systematic reviews (53, 54). Most of them also used MIMIC (38), not the most recent version IV, for model development or validation. The readmission intervals ranged from 48 hours (46, 47, 52) to 72 hours (26, 50, 51), 7 days (48), 30 days (40, 44, 49), and anytime until hospital discharge (41–43). A single study considers multiple intervals of 24 hours, 72 hours, 7 days, 30 days, and anytime (45). We chose an ICU readmission interval of 3 days because clinicians at the ANIT-UKM expressed that it would include relevant medical conditions that they could act upon before discharging a patient and, hence, would be most useful in practice. Also, we considered it a good trade-off between having sufficient follow-up and preventing exclusion of patients due to loss of follow-up (see step 7 in Figure 2). Previous studies have tested many models, and two (26, 27) mentioned the goal of developing interpretable models, but no validation by humans was performed. All studies reported ROC-AUC, which ranged from 0.64 (52) to 0.91 (42). Unfortunately, comparing the performance with the existing work is impossible for two reasons. First, we considered PR-AUC due to the label imbalance of ICU readmissions and optimized it in our experiments. However, none of the existing studies have reported this performance measure. One study contained a precision-recall curve (47), but no area under the curve. Second, we created a custom UKM cohort, and we used MIMIC-IV for external validation. None of the identified studies used these data. If the ROC-AUC is considered as a performance measure, our results are in the lower spectrum of the reported models. However, we did not optimize for it in our experiments.

A main goal of this study was to involve clinicians in the model development process to inspect the learned EBM and remove problematic risk functions. This approach showed mixed results. On the one hand, our collaboration confirmed that clinicians can easily grasp the concept of EBMs (36), making them a useful transparent model candidate for health care applications (55). Like LR, which is well-known in the medical domain, feature contributions are summed to a total log-odds score. This modularity also allowed to focus on a single risk function at a time. Confidence intervals and histograms over patient densities further helped to assess the relevance of function segments. For instance, it was possible to ignore fluctuations of risk functions in regions with few patients. In addition, our model development process enabled discussions with clinicians and encouraged a critical review of the model. Several aspects were raised for the first time, such as the problem with PTT measurements. Hence, with EBMs, stakeholders can be involved in the development process to establish trust, which could ultimately lead to higher adoption rates (13). Moreover, we identified and removed 18 risk functions due to the lack of interpretability, undesirable data artifacts, and contradiction of medical knowledge. This demonstrates the capability of EBMs to enable the identification and removal of undesirable components. This would have been impossible with a black box ML model (10, 12). Lastly, model inspection led to a performance increase on the hold-out data, which suggests better generalization.

However, we also observed several shortcomings during the model inspection. Of the 85 risk functions, 33 were labeled as problematic, of which 17 were not interpretable. Reducing a patient cohort to one or two features and considering a fixed time interval before discharge are counter to typical clinical practice, where many variables are usually integrated over a long time horizon. Thus, it was often difficult to create an intuition about the effect of certain risk functions. Also, for meaningful interpretation of EBMs, it is necessary to understand the model inputs (24, 55). In particular, interpretability was hindered by variables and descriptive statistics that are less common in clinical practice. One workaround would be to let clinicians choose interpretable features a priori. In addition, the shapes of risk functions sometimes showed a fluctuating behavior (36). We already increased the bin size to prevent these artifacts, but some still occurred in the final model. Another major issue was drawing the line between the inclusion and exclusion of risk functions. Most functions showed problematic behaviors. Thus, we decided to exclude only functions with a problem that affected a considerable part of the cohort. However, this decision rule is vague, and we expect low interrater reliability. We think it could be helpful to have a clear application scenario to determine more specific rules for exclusion. Moreover, we observed that it was more difficult to justify the exclusion of less interpretable functions and that the team relied on the EBM algorithm to find relevant associations in the data (56, 57).

This work has limitations. Even though the prediction of ICU readmission is a relevant medical problem, it can be difficult to turn predictions into actions when institutional factors such as insufficient ICU beds must be considered. No multicenter cohort was used for the development and validation of our prediction model, so the external validity of our results is low. Also, the data quality of the local cohort was limited, and our experiments only focused on a single interpretable model. External validation on the MIMIC-IV database was only performed for two models, and no in-depth analysis was performed for the improved performance. Moreover, interpretability should be evaluated in the context of its end task (14). Ideally, this could be increased trust leading to higher adoption of the system or even improved patient outcomes. We limited our analysis to prediction performance, the identification of problematic risk functions, and qualitative feedback. Moreover, no rigorous set of rules has been established for model inspection, so the process would likely exhibit low interrater reliability. The confidence intervals of the performance were only estimated on five temporal splits, and our EBM did not outperform the existing ML models by a large margin. Lastly, automatic risk function selection for EBMs might have removed important confounders, making it impossible to detect them during the model inspection.

## Conclusion

We demonstrated a procedure to develop a transparent EBM model for the prediction of 3 day ICU readmission that involved clinicians to inspect and verify the learned model. The EBM performed on par with or outperformed state-of-the-art black box ML models such as GBMs and RNNs. This suggests that a simple inherently interpretable model might suffice for clinical use in cases with low- to medium-dimensional data, while allowing a high level of human control. Evaluation of the model inspection revealed that an EBM model can facilitate a critical review with clinicians and enables identification of problematic components.

## Data availability statement

The patient datasets in this article are not readily available to protect patient privacy. The MIMIC-IV dataset used for external validation is available from https://doi.org/10.13026/s6n6-xd98. All the code used for the experiments is available from https://doi.org/10.5281/zenodo.5627167.

## Ethics statement

The studies involving human participants were reviewed and approved by the ethical review board of the medical chamber Westfalen-Lippe approved this study (reference number: 2020-526-f-S). Written informed consent for participation was not required for this study in accordance with the national legislation and the institutional requirements.

## Author contributions

SH designed the study, developed the code for all experiments, and wrote the manuscript. SH, TV, and CE performed the data pre-processing, cohort selection, and experiments. All authors provided critical feedback and helped shape the research and manuscript. All authors contributed to the article and approved the submitted version.

## Funding

This work was supported by the German Research Foundation (Deutsche Forschungsgemeinschaft, DFG Grants DU 352/11-1 and DU 352/11-2).

## Conflict of interest

The authors declare that the research was conducted in the absence of any commercial or financial relationships that could be construed as a potential conflict of interest.

## Publisher's note

All claims expressed in this article are solely those of the authors and do not necessarily represent those of their affiliated organizations, or those of the publisher, the editors and the reviewers. Any product that may be evaluated in this article, or claim that may be made by its manufacturer, is not guaranteed or endorsed by the publisher.
