# Supplementary material for: Development and validation of an interpretable 3 day intensive care unit readmission prediction model using explainable boosting machines
Source: Front Med (Lausanne). 2022 Aug 23;9:960296. doi: 10.3389/fmed.2022.960296 (PMC9445989; doi:10.3389/fmed.2022.960296)
Supplement: Supplementary file 2 [file Data_Sheet_2.pdf]

Supplementary Material

Figure 1. Example of the hospital stays with according ICU stays and labels.

Table 1. Table with detailed information of included ICUs.

Figure 2. Flow chart of variable selection and feature generation for UKM cohort.

Table 2. Table of all included variables grouped by feature classes.

Description of data cleaning of included variables

Table 3. Overview of feature classes.

Table 4. Features generated in addition to the standard feature classes

Description of ML models for comparison

Figure 3. Data splits for the experiments.

Settings used during parameter tuning of all models

Qualitative feedback during EBM model inspection

Cohort selection and data processing for external validation with MIMIC-IV

Figure 5. Performance during risk function selection for EBM and feature selection for LR models.

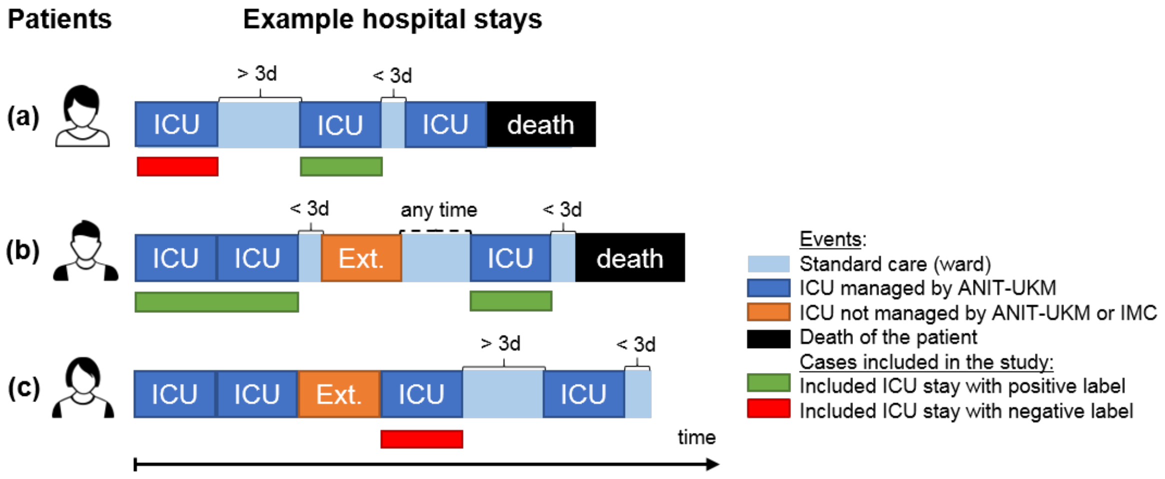

Figure 1. Example of the hospital stays with according ICU stays and labels. The event sequence is represented with colored blocks. Included ICU stays have a green (positive label) or red (negative label) bar below them. Transfers at excluded ICUs or IMC wards (orange) or standard care (light blue) were never included in the study, so there are no bars below them. However, readmission to an excluded ICU or IMC ward after discharge from an included ICU was considered for labeling. (a) The first two ICU transfers are included as negative and positive instances, because readmission happened after three days and within three days, respectively. The third ICU transfer is excluded due to death at the ICU. (b) The first two ICU transfers are consecutively, hence they are merged and considered as a single stay. It gets a positive label because readmission to an excluded ICUs or IMC ward occurs within three days. The last ICU transfer is also labeled positively because the patient dies within three days. (c) The first two ICU transfers are not included because they are merged but directly followed by a transfer to an excluded ICUs or IMC ward. However, the subsequent transfer at an included ICU is included with a negative label. The last ICU transfer is excluded because the follow-up period within the hospital is less than three days.

Table 1. Table with detailed information of included ICUs. All ICUs are managed by the ANIT-UKM. The number of patients and transfers in rows six and seven corresponds to the input of cohort selection. The last two rows represent the UKN cohort. Note that they do not sum up to the total number of patients and ICU stays because one patient or stay can be associated with more than one ICU.

**ICU**	**ICU 1**	**ICU 2**	**ICU 3**	**ICU 4**
Full name	Intensivtherapie I	Intensivstation des Herzzentrums	Intensivtherapie II	Perioperative Anästhesiestation
Short name	19A OST	19B OST	ANAES INT 2	ANAES PAS
Description	Surgical intensive care unit	Surgical intensive care unit	Surgical intensive care unit	Perioperative intensive care unit
Data in PDMS since	2/1/2001	11/1/2005	5/1/2003	2/1/2001
Number of beds[1]	11	11	11	7
Number of patients 2006-2019	7,389	10,372	6,231	17,061
Number of transfers 2006-2019	9,422	13,988	9,368	26,296
Number of included patients	4,008	5,962	1,031	10,644
Number of included ICU stays	4,008	7,576	1,354	16,233

[1] These are estimated average values because the number of beds changed several times over the years.

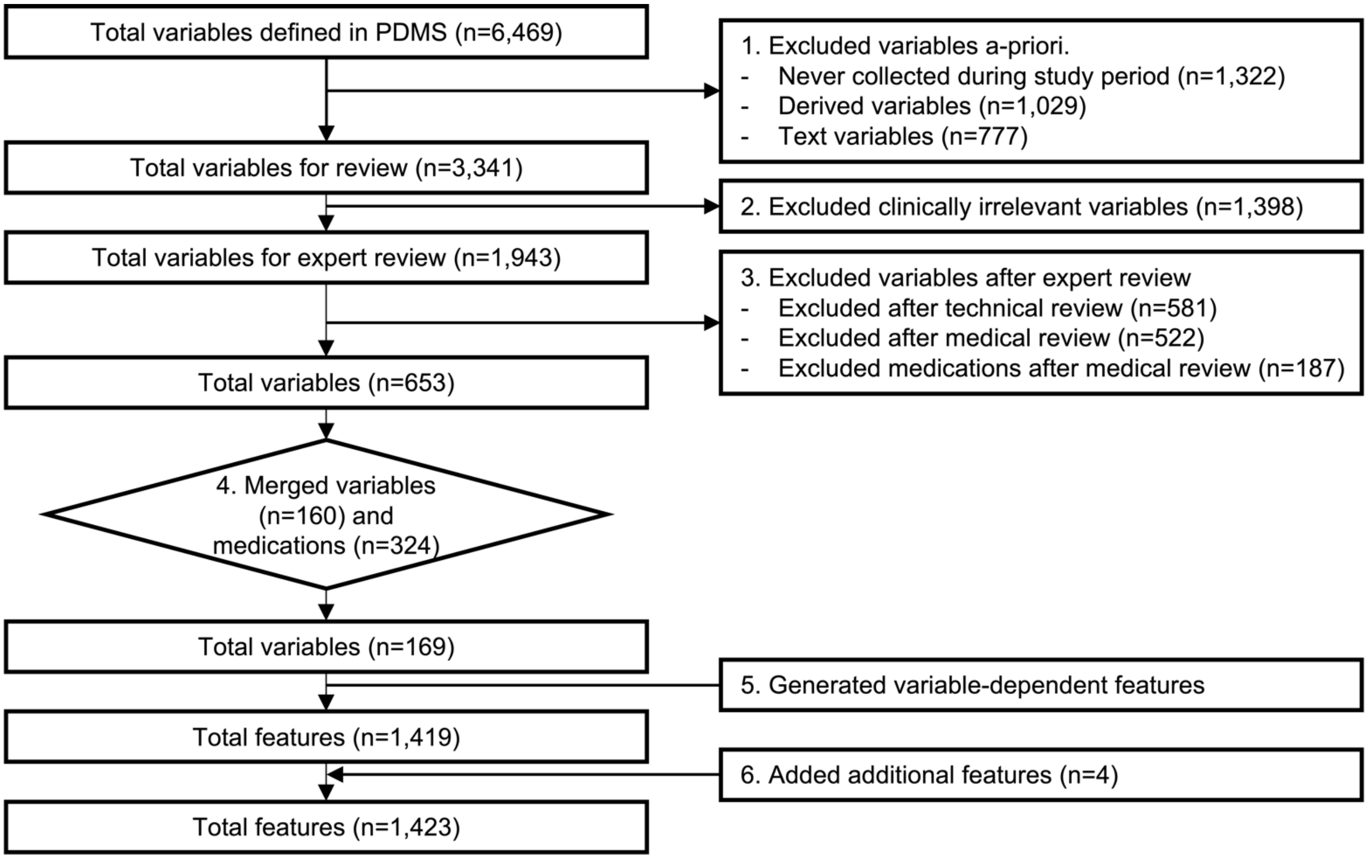

Figure 2. Flow chart of variable selection and feature generation for UKM cohort. First, 3,128 variables were excluded a-priori. Next, we reviewed the variables for their clinical relevance (n=1,398). During the technical and medical review, we excluded 581 and 709 further variables. Many variables collected the same data, so we merged 160 non-medication and 324 medication entries. The remaining 169 variables were assigned into feature classes from which we generated 1,419 features. Four additional static features were added afterwards.

Table 2. Table of all included variables grouped by feature classes.

Variable name	type	unit	Non-missing values in feature interval	median sampling interval	statistics during feat interval	Missing values in feature interval	Imputation value
**Feature class: static per patient**							
AIDS	categorical		3	2 d 09:37:00	True (n=3)	15584	-
Age	continuous	years	15639	5 d 07:09:30	median: 66.00, min: 18.00, max: 112.00	0	-
BMI	continuous	kg/m2	28261	3 d 00:00:00	median: 27.17, min: 11.30, max: 73.28	482	-
Gender	categorical		15405	03:03:00	Male (n=10519), Female (n=4886)	0	-
Height	continuous	cm	14709	02:47:00	median: 173.00, min: 63.00, max: 210.00	448	-
Hematologic malignancy	categorical		74	2 d 09:55:00	True (n=74)	15516	-
Metastatic cancer	categorical		651	2 d 11:55:00	True (n=651)	14964	-
Weight	continuous	kg	29809	2 d 21:13:00	median: 81.40, min: 30.00, max: 227.00	141	-
**Feature class: static per hospital stay**							
Admission origin	categorical		17402	2 d 19:43:30	operating room (n=14258),<br>general ward (n=1542),<br>other hospital (n=781),<br>emergency (n=306),<br>heart alarm (n=177),<br>ICU anesthesiology (n=133),<br>observation station surgery (n=101),<br>ICU non-anesthesiology (n=61),<br>intermediate care (n=43)	377	-
Admission reason	categorical		16197	2 d 11:54:00	Cardiovascular disease (n=11093),<br>Neoplasm (n=1761),<br>Neurological disorder (n=1141),<br>Respiratory disorder (n=943),<br>Trauma or bleeding (n=572),<br>Sepsis or infection (n=300),<br>GI disorder (n=247),<br>Other (n=140)	1132	-

Hygienic precautions	categorical		525	2 d 22:25:30	True (n=525)	15205	-
Patient class	categorical		15557	14:47:00	Inpatient (n=14129),<br>Emergency (n=1428)	13	Inpatient
Responsible clinic	categorical		14570	1 d 10:52:30	Cardiothoracic surgery (n=10788),<br>Thoracic surgery (n=873),<br>Orthopedic surgery (n=690),<br>Trauma surgery (n=519),<br>Neurosurgery (n=477),<br>Other (n=346),<br>General surgery (n=244),<br>Otorhinolaryngology (n=197),<br>Gynaecology (n=173),<br>Urology (n=156),<br>Oral and maxillofacial surgery (n=107)	74	Other
Type of admission	categorical		16938	2 d 10:09:00	Scheduled surgical (n=12684),<br>Medical (n=2493),<br>Unscheduled surgical (n=1761)	492	-
**Feature class: static per ICU stay**							
Any heart arrhythmia occurred	categorical		258810	01:00:00	True (n=258810)	10013	-
Has decubitus	categorical		8194	16:00:00	True (n=8194)	14505	-
Shows aphasia or dysarthria	categorical		676	08:00:00	True (n=676)	15408	-
**Feature class: time series high**							
Body core temperature	continuous	°C	3281601	00:15:00	median: 37.30, min: 25.00, max: 44.70	400	37.3
Diastolic blood pressure	continuous	mmHg	5028818	00:15:00	median: 57.00, min: 20.00, max: 300.00	14	57
Estimated respiratory rate	continuous		2567702	00:15:00	median: 20.00, min: 6.00, max: 100.00	6062	-
FiO2	continuous	%	1397347	00:15:00	median: 40.00, min: 0.00, max: 100.00	644	-
Heart rate	continuous	bpm	5679878	00:15:00	median: 83.00, min: 30.00, max: 300.00	0	-
Mean blood pressure	continuous	mmHg	5080902	00:15:00	median: 75.00, min: 20.00, max: 300.00	15	75

O2 saturation	continuous	%	5448554	00:15:00	median: 97.00, min: 0.00, max: 101.70	1	97.0
PAP - PEEP	continuous	mmHg	802165	00:15:00	median: 13.00, min: -34.00, max: 66.00	5336	-
Systolic blood pressure	continuous	mmHg	5026861	00:15:00	median: 114.00, min: 20.00, max: 300.00	14	114
**Feature class: time series medium**							
Anion gap	continuous	mEq/L	283297	02:40:00	median: 5.00, min: -97.70, max: 97.60	1606	-
BE	continuous	mmol/L	450028	02:43:00	median: 0.30, min: -24.90, max: 25.00	50	0.3
Bicarbonate	continuous	mmol/L	450284	02:43:00	median: 24.50, min: 0.00, max: 74.40	50	24.5
COHb	continuous	%	524960	02:20:00	median: 1.40, min: 0.00, max: 7.90	61	1.4
Calcium	continuous	mmol/L	453457	02:41:00	median: 1.16, min: 0.00, max: 4.36	49	1.16
Chloride	continuous	mmol/L	448707	02:43:00	median: 109.00, min: 0.00, max: 199.00	50	109.0
Glucose	continuous	mg/dL	566205	02:18:00	median: 134.00, min: 0.00, max: 1196.00	37	134.0
Hematocrit	continuous	%	499803	02:33:00	median: 29.80, min: 0.00, max: 69.40	39	29.8
Hemoglobin	continuous	mmol/L	587605	02:13:00	median: 9.60, min: 0.00, max: 28.60	40	9.6
Lactate	continuous	mmol/L	533796	02:23:00	median: 1.10, min: 0.00, max: 26.00	55	1.1
MetHb	continuous	%	524746	02:20:00	median: 0.90, min: 0.00, max: 15.70	61	0.9
O2Hb	continuous	%	275139	02:41:00	median: 95.30, min: 0.00, max: 100.00	1616	-
Potassium	continuous	mmol/L	450215	02:49:00	median: 4.30, min: 0.00, max: 10.00	47	4.3
RAS scale	continuous		1280104	01:00:00	median: 0.00, min: -5.00, max: 5.00	4	0
RHb	continuous		273565	02:41:00	median: 2.10, min: 0.00, max: 87.60	1628	-
Sodium	continuous	mmol/L	448999	02:48:00	median: 138.00, min: 0.00, max: 211.00	49	138.0
pCO2	continuous	mmHg	444251	02:44:00	median: 38.90, min: 0.00, max: 145.90	53	38.9
pH	continuous		435866	02:46:00	median: 7.42, min: 4.77, max: 7.98	68	7.417
pO2	continuous	mmHg	443732	02:44:00	median: 88.80, min: 0.00, max: 719.00	54	88.8

paO2/FiO2	continuous	mmHg /FiO2	382127	02:44:00	median: 246.00, min: 15.00, max: 2877.62	1012	246.0
**Feature class: time series low**							
Albumin	continuous	g/dL	5695	23:45:00	median: 2.80, min: 0.00, max: 5.30	13676	-
Alkaline phosphatase	continuous	U/L	10993	1 d 00:00:00	median: 74.00, min: 0.00, max: 499.00	11714	-
Antithrombin III	continuous	%	3064	12:30:00	median: 68.00, min: 30.00, max: 131.00	13593	-
Bilirubin total	continuous	mg/dL	65621	23:45:00	median: 0.60, min: 0.00, max: 49.70	2573	-
Blood Urea Nitrogen	continuous	mg/dL	81272	23:45:00	median: 21.00, min: 0.00, max: 463.00	771	-
C-reactive protein	continuous	mg/dL	76062	23:45:00	median: 6.70, min: 0.00, max: 58.70	1224	-
CK	continuous	U/L	104111	23:00:00	median: 302.00, min: 0.00, max: 10000.00	121	302.0
CK-MB	continuous	U/L	75249	13:45:00	median: 28.00, min: 0.00, max: 4116.00	1726	-
Cholinesterase	continuous	U/L	5485	23:45:00	median: 3699.00, min: 0.00, max: 14230.00	13405	-
Creatinine	continuous	mg/dL	94062	23:30:00	median: 0.90, min: 0.00, max: 59.80	97	0.9
Erythrocytes	continuous	millions/µL	49093	23:45:00	median: 3.20, min: 0.00, max: 71.00	6034	-
Fibrinogene	continuous	mg/dL	3642	10:00:00	median: 261.00, min: 200.00, max: 350.00	13230	-
GCS Eye	continuous		49324	1 d 00:00:00	median: 4.00, min: 1.00, max: 4.00	2841	4
GCS Motor	continuous		49325	1 d 00:00:00	median: 6.00, min: 1.00, max: 6.00	2841	6
GCS Verbal	continuous		49325	1 d 00:00:00	median: 5.00, min: 1.00, max: 5.00	2841	5
GCS score	continuous		49321	1 d 00:00:00	median: 15.00, min: 3.00, max: 15.00	2841	15
GOT (AST)	continuous	U/L	53538	23:45:00	median: 45.00, min: 0.00, max: 4992.00	3171	-
GPT (ALT)	continuous	U/L	94296	23:30:00	median: 26.00, min: 0.00, max: 4986.00	197	26.0
Gamma-GT	continuous	U/L	69286	23:45:00	median: 40.00, min: 0.00, max: 500.00	1864	-
LDH	continuous	U/L	30388	23:45:00	median: 275.00, min: 0.00, max: 1000.00	7831	-
Leucocytes	continuous	thousand/µL	95074	23:30:00	median: 11.49, min: 0.00, max: 99.41	117	11.49
Lipase	continuous	U/L	15405	1 d 00:00:00	median: 25.00, min: 0.00, max: 498.00	10235	-
MCH	continuous	pg	32522	23:45:00	median: 29.60, min: 0.00, max: 43.70	9424	-

MCHC	continuous	g/dL	32525	23:45:00	median: 33.20, min: 0.00, max: 100.00	9424	-
MCV	continuous	fL	32527	23:45:00	median: 88.80, min: 0.00, max: 140.50	9423	-
Magnesium	continuous	mmol/L	9155	1 d 00:00:00	median: 0.86, min: 0.14, max: 33.81	12296	-
Maximum decubitus stage	continuous		8194	16:00:00	median: 2.00, min: 1.00, max: 4.00	14505	-
Osmolality	continuous	mOsm/kg	6069	1 d 00:00:00	median: 297.00, min: 200.00, max: 350.00	13747	-
PTT	continuous	s	126767	13:15:00	median: 42.00, min: 0.00, max: 236.00	84	42.0
Phosphate	continuous	mg/dL	73646	23:45:00	median: 3.30, min: 0.00, max: 12.90	1357	-
Procalcitonin	continuous	ng/mL	28417	1 d 00:00:00	median: 0.40, min: 0.00, max: 5.00	8982	-
Protein	continuous	g/dL	4751	1 d 00:00:00	median: 5.30, min: 0.00, max: 8.70	14001	-
Protein (urine)	continuous	mg	5732	1 d 00:26:00	median: 0.00, min: 0.00, max: 150.00	12920	-
Prothrombin time (INR)	continuous		107160	14:22:00	median: 1.19, min: 0.00, max: 90.00	1180	-
Quick	continuous	%	117782	14:15:00	median: 75.00, min: 0.00, max: 140.00	88	75.0
T3 free	continuous	ng/dL	6352	6 d 23:30:00	median: 1.90, min: 0.40, max: 6.70	11762	-
T4 free	continuous	ng/dL	6661	6 d 23:15:00	median: 1.20, min: 0.20, max: 4.10	11612	-
TSH	continuous	μU/mL	8264	6 d 00:30:00	median: 1.30, min: 0.02, max: 86.00	10650	-
Thrombin time	continuous	s	3318	23:45:00	median: 20.00, min: 15.00, max: 50.00	13730	-
Thrombocytes	continuous	thousand/μL	104086	23:00:00	median: 166.00, min: 0.00, max: 1000.00	87	166.0
eGFR	continuous	L	92259	23:45:00	median: 77.61, min: 0.53, max: 306.55	108	77.60777
**Feature class: flow**							
Blood volume in	continuous	mL	28183	01:00:00	median: 250.00, min: 0.00, max: 3000.00	10917	-
Blood volume out	continuous	mL	1130600	00:00:00	median: 0.00, min: 0.00, max: 5000.00	1861	-
Cardiac stimulants (epinephrine equivalence dosage)	continuous		292237	00:20:00	median: 11.08, min: 0.00, max: 10556.40	11777	-
Drainage volume out	continuous	mL	34788	01:00:00	median: 7.00, min: 0.00, max: 4000.00	14451	-
Enteral nutrition volume in	continuous	mL	596044	01:00:00	median: 80.00, min: 0.00, max: 2550.00	357	-

Feeding tube volume out	continuous	mL	24857	06:55:00	median: 50.00, min: 1.00, max: 2000.00	11390	-
Glucocorticoids (cortison equivalence dosage)	continuous		44409	01:00:00	median: 4.00, min: 0.03, max: 5500.00	12378	-
Infusion volume in	continuous	mL	6914612	00:00:00	median: 1.00, min: 0.00, max: 100000.00	168	-
Norepinephrine and Dopamine (norepinephrine equivalence dosage)	continuous		563212	00:20:00	median: 79.20, min: 0.00, max: 12960.00	4792	-
Plasma expander volume in	continuous	mL	29669	01:00:00	median: 500.00, min: 0.00, max: 1500.00	10502	-
Stool volume out	continuous	mL	71488	06:00:00	median: 100.00, min: 1.00, max: 2000.00	8830	-
Ultrafiltrate volume out	continuous	mL	11942	06:00:00	median: 500.00, min: 0.00, max: 5200.00	14984	-
Urine volume out	continuous	mL	972240	01:00:00	median: 80.00, min: 1.00, max: 5000.00	116	-
**Feature class: interventions**							
Antithrombotic agents prophylactic dosage	categorical		192060	01:00:00	True (n=192060)	3261	-
Antithrombotic agents therapeutic dosage	categorical		869689	00:20:00	True (n=869689)	7775	-
Arterial line exists	categorical		139666	08:00:00	True (n=139666)	1072	-
Bladder catheter exists	categorical		138885	08:00:00	True (n=138885)	1259	-
Bronchoscopy performed	categorical		1020	2 d 04:07:30	True (n=1020)	15050	-
CCO exists	categorical		58782	00:15:00	True (n=58782)	15341	-
Cardiopulmonary resuscitation performed	categorical		153	01:50:30	True (n=153)	15467	-
Cardioversion performed	categorical		642	01:55:00	True (n=642)	15266	-
Central venous catheter exists	categorical		133589	08:00:00	True (n=133589)	2713	-
Central venous line exists	categorical		116510	08:00:00	True (n=116510)	2922	-
Chest tube exists	categorical		431150	01:00:00	True (n=431150)	3549	-
Defibrillation performed	categorical		143	00:20:00	True (n=143)	15536	-
Dialysis or CVVH performed	categorical		11930	06:00:00	True (n=11930)	14984	-
ECMO or RVAD	categorical		31631	01:00:00	True (n=31631)	15455	-

Feeding system exists	categorical		73011	05:00:00	True (n=73011)	12533	-
IABP	categorical		15390	01:00:00	True (n=15390)	15269	-
ICP probe exists	categorical		242	08:00:00	True (n=242)	15576	-
Is on automatic ventilation	categorical		692027	00:15:00	True (n=692027	4196	-
LVAD	categorical		119238	01:00:00	True (n=18314)	15191	-
Shaldon catheter exists	categorical		18314	08:00:00	True (n=93302)	14506	-
Thrombosis prophylaxis performed	categorical		93302	10:00:00	True (n=93302)	2018	-
Tracheal cannula exists	categorical		17465	08:00:00	True (n=17465)	15127	-
Tracheal secretion cleaned	categorical		80477	03:00:00	True (n=80477)	7098	-
Tubus exists	categorical		24358	08:00:00	True (n=24358)	6635	-
**Feature class: medication**							
ACE inhibitors plain and combinations	categorical		21311	12:00:00	Enalapril (C09AA02) (n=16763),Ramipril (C09AA05) (n=3595),Acerbon (C09AA03) (n=393),Lisinopril Tbl. (C09AA03) (n=311),Captopril (C09AA01) (n=227),Delix plus (C09BA25) (n=22)	11347	-
Anesthetics	categorical		261094	00:20:00	Propofol Perf. (N01AX10) (n=246642),Ketamin-S_Perfusor (N01AX03) (n=8088),Propofol (N01AX10) (n=2027),Remifentanil Perf. (N01AH06) (n=1990),Isofluran (N01AB06) (n=1744),Etomidat-Lipuro (N01AX07) (n=303),Ketamin-S (N01AX03) (n=180),Thiopental Perf. (N01AF03) (n=90),Thiopental (N01AF03) (n=22),Hydroxybuttersäure Perf. (N01AX11) (n=8)	5051	-
Angiotensin II receptor blockers	categorical		401	22:48:00	Candesartan (C09CA06) (n=219),Valsartan (C09CA03) (n=92),Lorzaar Tbl. (C09CA01) (n=88),Irbesartan (C09CA04) (n=2)	15433	-
Antiandrenergic agents, centrally acting	categorical		215952	00:20:00	Clonidin Perf. (C02AC01) (n=157259),Dexmedetomidin (C02AC) (n=50275),Catapresan (C02AC01) (n=6792),Moxonidin (C02AC05) (n=1626)	8255	-
Antiandrenergic agents, peripherally acting	categorical		44893	01:00:00	Urapidil Perf. (C02CA06) (n=38927),Urapidil (C02CA06) (n=5966)	12751	-
Antiarrhythmics, class I and III	categorical		100470	00:21:00	Amiodaron (Cordarex) Perf. (C01BD01) (n=86485),Amiodaron (Cordarex) Tbl (C01BD01) (n=9798),Amiodaron (Cordarex) (C01BD01) (n=3319),Ajmalin Perf. (C01BA05) (n=614),Flecainid (C01BC04) (n=174),Ajmalin (C01BA05) (n=50),Propafenon (C01BC03) (n=15),Propafenon Perf. (C01BC03) (n=15)	13148	-
Antibacterials for systemic use	categorical		104097	06:00:00	Cefuroxim (J01DC02) (n=19797),Meropenem (J01DH02) (n=19269),Piperacillin/Tazobactam (J01CR05) (n=13523),Cephazolin (J01DB04) (n=13097),Vancomycin (J01XA01) (n=6044),Flucloxacillin (J01CF05) (n=4030),Cefotaxim (J01DD01) (n=3036),Clindamycin (J01FF01) (n=2390),Ciprobay (J01MA02) (n=2195),Ampicillin (J01CA01) (n=2006),Ceftazidim (J01DD02) (n=1987),Gentamicin (J01GB03) (n=1651),Linezolid (J01XX08) (n=1451),Metronidazol (Clont) (J01XD01) (n=1337),Fosfomycin (J01XX01) (n=1263),Erythromycin (J01FA01) (n=1240),Penicillin G (J01CE01) (n=1235),Ampicillin/Sulbactam (J01CR01) (n=1001),Ceftriaxon (J01DD04) (n=901),Daptomycin (J01XX09) (n=798),Cotrim (J01EA01) (n=749),Tigecyclin (J01AA12) (n=670),Amoxicillin/Clavulansäure (J01CR02) (n=655),Teicoplanin (J01XA02) (n=471),Colistin (J01XB01) (n=378),Imipenem (J01DH51) (n=363),Moxifloxacin_Tbl (J01MA14) (n=319),Clarithromycin (J01FA09) (n=310),Moxifloxacin (J01MA14) (n=288),Baypen (J01CA10) (n=278),Combactam (J01CG01) (n=245),Metronidazol Tbl. (J01XD01) (n=220),Levofloxacin (J01MA12) (n=170),Ertapenem (J01DH03) (n=159),Tobramycin (J01GB01) (n=138),Amoxicillin (J01CA04) (n=130),Ciprofloxacin (J01MA02) (n=126),Cubicin (J01XX09) (n=117),Biklin	4824	-

					(J01GB06) (n=33),Flucloxacillin p.o. (J01CF05) (n=11),Kepinol Tbl. (J01EE01) (n=10),Pipril (J01CA12) (n=4),Binotal (J01CA01) (n=1),Polymyxin B (J01XB02) (n=1)		
Antidepressants	categorical		5572	1 d 00:00:00	Escitalopram (N06AB10) (n=2335),Saroten (N06AA09) (n=1341),Cipramil Tbl. (N06AB04) (n=1117),Remergil (N06AX11) (n=779)	14801	-
Antiemetic preparation	categorical		1344	1 d 00:00:00	Folsaeure (B03BB01) (n=478),Ferrosanol (B03AA01) (n=399),Vit.B12 (B03BA53) (n=214),Eisen-III (i.v.) (B03AC01) (n=85),Darbepoetin alfa (B03XA02) (n=80),Eisencarboxymaltose (B03AC01) (n=64),Erythropoetin (B03XA01) (n=20),Eisen-II-sulfat (B03AA07) (n=4)	15206	-
Antiepileptics	categorical		16286	01:00:00	Pregabalin (N03AX16) (n=5290),Levetiracetam (N03AX14) (n=2978),Levetiracetam Perf. (N03AX14) (n=1969),Orfiril Perf. (N03AG01) (n=1804),Neurontin (N03AX12) (n=1578),Phenytoin Perf. (N03AB02) (n=698),Carbamazepin (N03AF01) (n=447),Orfiril (N03AG01) (n=365),Valproinsäure (N03AG01) (n=350),Lacosamid (N03AX18) (n=246),Valproinsäure Perf. (N03AG01) (n=158),Frisium (N03A) (n=123),Clonazepam Perf. (N03AE01) (n=104),Phenytoin (N03AB02) (n=104),Clonazepam (N03AE01) (n=71),Phenobarbital (N03AA02) (n=1)	14480	-
Antihemorrhagics	categorical		5449	01:00:00	Cyclocapron (B02AA02) (n=3429),Konakion (B02BA02) (n=834),Haemocomplettan (B02BB01) (n=463),PPSB (B02BD01) (n=380),Konakion-Tropfen (B02BA02) (n=270),Trasylol (B02AB01) (n=24),Fibrogammin (B02BD07) (n=20),Novo7 (B02BD02) (n=16),Haemate (B02BD06) (n=13)	14228	-
Antihypertensiva	categorical		718002	00:20:00	Clonidin Perf. (C02AC01) (n=157259),Glyceroltrinitrat Perf. (C01DA02) (n=123303),Furosemid Perf. (C03CA01) (n=65459),Dihydralazin (C02DB01) (n=63836),Dexmedetomidin (C02AC) (n=50275),Torasemid (C03CA04) (n=42864),Urapidil Perf. (C02CA06) (n=38927),Beloc-Zok (C07AB02) (n=26663),Nitroprussidnatrium Perf. (C02DD01) (n=26265),Amlodipin (C08CA01) (n=23563),Enalapril (C09AA02) (n=16763),Bisoprolol (C07AB07) (n=16671),Furosemid (C03CA01) (n=13719),Metoprolol (C07AB02) (n=10819),Catapresan (C02AC01) (n=6792),Urapidil (C02CA06) (n=5966),Spironolacton i.v. (C03DA01) (n=4623),Ramipril (C09AA05) (n=3595),Spironolacton Tbl. (C03DA01) (n=3540),ISDN (C01DA08) (n=2345),Querto (C07AG02) (n=2039),Xipamid (C03BA10) (n=1900),Moxonidin (C02AC05) (n=1626),Diltiazem Tbl. (C08DB01) (n=1389),Hydrochlorothiazid (C03AA03) (n=1383),Lonolox (C02DC01) (n=892),Verapamil (C08DA01) (n=594),Nebivolol (C07AB12) (n=579),Acerbon (C09AA03) (n=393),Alprostadil (C04AG01) (n=373),Verapamil Perf. (C08DA01) (n=370),Esmolol Perf. (C07AB09) (n=320),Lisinopril Tbl. (C09AA03) (n=311),Dociton (C07AA05) (n=297),Captopril (C09AA01) (n=227),Eplerenon (C03DA04) (n=224),Candesartan (C09CA06) (n=219),Trental Perf. (C04AD03) (n=167),Nimodipin Saft (C08CA06) (n=163),Lercanidipin (C08CA13) (n=151),Carmen (C08CA13) (n=133),Nimodipin Perf. (C08CA06) (n=126),Molsidomin (Corvaton) (C01DX12) (n=120),Nimodipin Tbl. (C08CA06) (n=101),Diltiazem Perf. (C08DB01) (n=97),Valsartan (C09CA03) (n=92),Lorzaar Tbl. (C09CA01) (n=88),Nitro-Spray (C01DA02) (n=86),Sotalex (C07AA07) (n=69),Molsidomin (C01DX12) (n=66),Diltiazem (C08DB01) (n=62),Nifedipin Tbl. (C08CA05) (n=47),Unat (C03CA04) (n=24),Delix plus (C09BA25) (n=22),Nifedipin Perf. (C08CA05) (n=3),Irbesartan (C09CA04) (n=2)	2121	-
Antimycotics for systemic use	categorical		4313	1 d 00:00:00	Voriconazol Tbl. (J02AC03) (n=1266),Micafungin (J02AX05) (n=1137),Caspofungin (J02AX04) (n=606),Fluconazol (J02AC01) (n=555),Voriconazol (J02AC03) (n=372),Fluconazol Tbl. (J02AC01) (n=129),Posaconazol (J02AC04) (n=88),Anidulafungin (J02AX06) (n=80),AmBisome (J02AA01) (n=61),Flucytosin (J02AX01) (n=19)	15221	-
Antipsychotics (neuroleptics)	categorical		16896	07:24:00	Haldol (N05AD01) (n=9988),Quetiapin (N05AH04) (n=2563),Risperidon (N05AX08) (n=2262),Melperon (N05AD03) (n=1541),Pipamperon-Saft (N05AD05) (n=398),Promethazin (N05A) (n=144)	13649	-
Antithrombotic agents excl. Platelet inhibitors and enzymes	categorical		1044845	00:20:00	Heparin Perf. (B01AB01) (n=847558),Argatroban (B01AE03) (n=162454),Clexane (B01AB05) (n=29516),Heparin (B01AB01) (n=2770),Marcumar (B01AA04) (n=2052),Danaparoid-Na (B01AB09) (n=263),Fondaparinux (B01AX05) (n=220),Rivaroxaban (B01AF01) (n=6),Dabigatran (B01AE07) (n=4),Apixaban (B01AF02) (n=2)	1572	-
Antivirals for systemic use	categorical		4911	11:00:00	Aciclovir(Zovirax) (J05AB01) (n=2305),Cymeven (J05AB06) (n=2117),Valcyte (J05AB14) (n=482),Foscarnet (J05AD01) (n=4),Entecavir (J05AF10) (n=3)	15337	-

Anxiolytics, hypnotics and sedatives	categorical		17372	01:00:00	Midazolam Perf. (N05CD08) (n=8913),Lorazepam (N05BA06) (n=4924),Midazolam (N05CD08) (n=1530),Zolpidem (N05CF02) (n=825),Zopiclon (N05CF01) (n=408),Tranxilium (N05BA05) (n=304),Diazepam (N05BA01) (n=243),Rohypnol (N05CD03) (n=154),Bromazepam (N05BA08) (n=44),Temazepam (N05CD07) (n=25),Flurazepam (N05CD01) (n=1),Clomethiazol (N05CM02) (n=1)	12600	-
Arteriolar smooth muscle, agents acting on	categorical		90993	00:20:00	Dihydralazin (C02DB01) (n=63836),Nitroprussidnatrium Perf. (C02DD01) (n=26265),Lonolox (C02DC01) (n=892)	14015	-
Beta blocking agents	categorical		57457	12:00:00	Beloc-Zok (C07AB02) (n=26663),Bisoprolol (C07AB07) (n=16671),Metoprolol (C07AB02) (n=10819),Querto (C07AG02) (n=2039),Nebivolol (C07AB12) (n=579),Esmolol Perf. (C07AB09) (n=320),Dociton (C07AA05) (n=297),Sotalex (C07AA07) (n=69)	5931	-
Cardiac stimulants	categorical		312110	00:20:00	Epinephrin Perf. (C01CA24) (n=145660),Dobutamin Perf. (C01CA07) (n=133546),Milrinon Perf. (C01CE01) (n=20282),Levosimendan (C01CX08) (n=12068),Epinephrin (C01CA24) (n=554)	11776	-
Digitalis glycosides	categorical		3757	1 d 00:00:00	Digimerck (C01AA04) (n=1891),Lanicor (C01AA05) (n=1746),Lanitop (C01AA08) (n=78),Novodigal (C01AA02) (n=42)	14670	-
Diuretics	categorical		133736	01:00:00	Furosemid Perf. (C03CA01) (n=65459),Torasemid (C03CA04) (n=42864),Furosemid (C03CA01) (n=13719),Spironolacton i.v. (C03DA01) (n=4623),Spironolacton Tbl. (C03DA01) (n=3540),Xipamid (C03BA10) (n=1900),Hydrochlorothiazid (C03AA03) (n=1383),Eplerenon (C03DA04) (n=224),Unat (C03CA04) (n=24)	7785	-
Drugs for constipation	categorical		110436	08:00:00	Movicol (A06AD15) (n=53310),Laxoberal (A06AB08) (n=47723),Dulcolax (A06AB02) (n=7481),Practo-Klyss (A06AG20) (n=939),Bifiteral (A06AD11) (n=868),Prucaloprid (A06AX05) (n=88),Methylnaltrexon (A06AH01) (n=27)	3780	-
Drugs for functional gastrointestinal disorders	categorical		8482	08:00:00	MCP (Gastrosil) (A03FA01) (n=3599),Sab simplex (A03AX13) (n=2331),Neostigmin (A03) (n=1961),Buscopan (A03BB01) (n=230),Lefax (A03AX13) (n=186),Domperidon (A03FA03) (n=168),Robinul (A03AB02) (n=7)	13845	-
Drugs for obstructive airway diseases	categorical		35537	06:00:00	Sultanol (R03AC02) (n=10411),Berodual (R03AL01) (n=7918),Sultanol_Atrovent (R03AL02) (n=5187),Pulmicort DA (R03BA02) (n=2577),Formoterol (R03AC13) (n=1662),Reproterol Perf. (R03CC14) (n=1471),Budesonid (R03BA02) (n=1453),Orciprenalin Perf. (R03CB03) (n=1418),Berotec DA (R03AC04) (n=845),Tiotropiumbromid (R03BB04) (n=676),Bricanyl (R03CC03) (n=608),Theophyllin Perf. (R03DA04) (n=514),Symbicort (R03AK07) (n=349),Reproterol (R03CC14) (n=244),Theophyllin (R03DA04) (n=152),Flutide N forte DA (R03BA05) (n=41),Spiropent (R03CC13) (n=7),Orciprenalin (R03CB03) (n=4)	13419	-
Drugs for peptic ulcer and reflux without PPIs	categorical		13218	08:00:00	Ranitidin (Zantic) (A02BA02) (n=11974),Ulcogant (A02BX02) (n=1214),Gastrozepin (A02BX03) (n=24),Cimetidin (A02BA01) (n=6)	11554	-
Drugs used in diabetes	categorical		996126	00:20:00	Insulin Perf. (A10AB01) (n=988253),Insulin (A10AB01) (n=4988),Metformin (A10BA02) (n=2051),Insuman (A10AB01) (n=395),Sitagliptin (A10BH01) (n=198),Euglucon (A10BB01) (n=134),Insulin glargin (A10AE04) (n=78),Novorapid (A10AB05) (n=29)	5420	-
Glucocorticoids	categorical		44821	01:00:00	Hydrocortison Perf. (H02AB09) (n=27905),Solu-Decortin H (H02AB06) (n=5470),Decortin (H02AB07) (n=4897),Prednisolon (H02AB06) (n=3145),Dexamethason (H02AB02) (n=1360),Methylprednisolon (H02AB04) (n=1323),Hydrocortison (H02AB09) (n=721)	12376	-
Immunglobulins	categorical		270	22:00:00	Cytotect (J06BB09) (n=233),Pentaglobin (J06BA02) (n=16),Privigen (J06BA02) (n=14),Tetagam (J06BB02) (n=6),Varitect (J06BB03) (n=1)	15467	-
Immunsuppressants	categorical		79008	01:00:00	Ciclosporintabletten (L04AD01) (n=54402),Prograf (L04AD02) (n=8785),Cellcept (L04AA06) (n=7023),Sandimmun Optoral (L04AD01) (n=3778),Ciclosporin (L04AD01) (n=3608),Azathioprin (L04AX01) (n=557),ATG (L04AA18) (n=475),Everolimus (L04AA18) (n=260),Rapamune (L04AA10) (n=119),Alemtuzumab (L04AA34) (n=1)	15169	-

Inhalative vasodilatators	categorical		72185	01:59:00	Sildenafil (C02KX06) (n=27837),Ilomedin (C02KX08) (n=27741),NO (C02KX) (n=13711),Milrinon (C02KX) (n=1905),Bosentan (C02KX01) (n=957),Ilomedin-Perf. (C02KX08) (n=34)	14512	-
Lipid modifying agents	categorical		24342	1 d 00:00:00	Zocor (C10AA01) (n=8390),Atorvastatin (C10AA05) (n=6255),Simvastatin (C10AA01) (n=5787),Pravastatin (C10AA03) (n=3628),Ezetimib (C10AX09) (n=282)	8766	-
Mineral supplements	categorical		692876	00:20:00	Kalium Perf. (A12BA01) (n=617315),Calciumchlorid Perf. (A12AA07) (n=23516),Kalinor-Brause (A12BA02) (n=18355),NaCl 20proz. (A12CA01) (n=6925),Natriumphosphat (A12) (n=5486),Magnesiumaspartat (A12CC05) (n=5235),Magnesium p.o. (A12CC30) (n=4231),Calciumgluconat Perf. (A12AA03) (n=4184),Kalinor-Kps. (A12BA02) (n=2471),Magnesiumsulfat Perf. (A12CC02) (n=2328),Calciumgluconat (A12AA03) (n=744),Kalium (A12BA01) (n=678),Magnesiumaspartat Perf. (A12CC05) (n=404),Selenase (A12CE02) (n=400),Ideos (A12AX01) (n=260),Phosphat_Brausetbl (A12CX50) (n=183),Phosphat_Filmtbl (A12CX50) (n=89),NaCl 3proz (A12CA01) (n=49),NaCl Tablette (A12) (n=23)	5286	-
Minirin	categorical		275	12:00:00	Minirin (H01BA02) (n=275)	15375	-
Muscle relexants	categorical		2562	06:00:00	Cisatracurium (M03AC11) (n=1507),Esmeron (M03AC09) (n=531),Cisatracurium Perf. (M03AC11) (n=419),Baclofen (M03BX01) (n=71),Dantrolen (M03CA01) (n=26),Succinylcholin (M03AB01) (n=8)	14418	-
Non-opioid analgetics	categorical		59127	06:00:00	Novaminsulfon-Tropfen (N02BB02) (n=18636),Novaminsulfon (Novalgin) (N02BB02) (n=14675),Perfalgan (N02BE01) (n=11694),Benuron (N02BE01) (n=8739),Paracetamol (N02BE01) (n=3337),Arcoxia (M01AH05) (n=795),Ibuprofen (M01AE01) (n=662),Voltaren (M01AB05) (n=326),Indometacin (M01AB01) (n=260),Prodafalgan (N02BE01) (n=3)	5659	-
Norepinephrine and Dopamine	categorical		581798	00:20:00	Norepinephrin Perf. (C01CA03) (n=581700),Dopamin Perf. (C01CA04) (n=65),Norepinephrin (C01CA03) (n=33)	4788	-
Opioids	categorical		265173	01:00:00	Piritramid (N02AC03) (n=107223),Sufentanil Perf. (N02AB07) (n=75601),Piritramid Perf. (N02AC03) (n=26651),Palladon (N02AA03) (n=14227),Palladon retard (N02AA03) (n=13765),Hydromorphon (N02AA03) (n=12176),Morphin (N02AA01) (n=8476),Targin (N02AA05) (n=4938),Oxygesic (N02AA05) (n=988),Sufentanil (N02AB07) (n=400),Durogesic-Pflaster (N02AB03) (n=280),Fentanyl (N02AB03) (n=148),Fentanyl Perf. (N02AB03) (n=65),Levo-Methadon (N02AC52) (n=57),Fentanyl TTS (N02AB03) (n=51),MST_Granulat (N02AA01) (n=48),Pethidin (N02AB02) (n=39),Buprenorphin (N02AE01) (n=30),Tramadol (N02AX02) (n=10)	1754	-
Platelet aggregation inhibitors excl. Heparin	categorical		38581	1 d 00:00:00	ASS (B01AC06) (n=30526),Clopidogrel (B01AC04) (n=7156),Ticagrelor (B01AC24) (n=768),Prasugrel (B01AC22) (n=105),Aggrastat (B01AC17) (n=22),Aggrenox (B01AC36) (n=4)	6609	-
Proton pump inhibitor	categorical		49007	1 d 00:00:00	Pantoprazol (A02BC02) (n=26597),Nexium (A02BC05) (n=13581),Esomeprazol (A02BC05) (n=8559),Omeprazol (A02BC01) (n=270)	3204	-
Selective calcium channel blockers with direct cardiac effects	categorical		2512	01:00:00	Diltiazem Tbl. (C08DB01) (n=1389),Verapamil (C08DA01) (n=594),Verapamil Perf. (C08DA01) (n=370),Diltiazem Perf. (C08DB01) (n=97),Diltiazem (C08DB01) (n=62)	15357	-
Selective calcium channel blockers with mainly vascular effects	categorical		24287	04:00:00	Amlodipin (C08CA01) (n=23563),Nimodipin Saft (C08CA06) (n=163),Lercanidipin (C08CA13) (n=151),Carmen (C08CA13) (n=133),Nimodipin Perf. (C08CA06) (n=126),Nimodipin Tbl. (C08CA06) (n=101),Nifedipin Tbl. (C08CA05) (n=47),Nifedipin Perf. (C08CA05) (n=3)	11232	-
Vasodilatators used in cardiac diseases	categorical		125920	00:20:00	Glyceroltrinitrat Perf. (C01DA02) (n=123303),ISDN (C01DA08) (n=2345),Molsidomin (Corvaton) (C01DX12) (n=120),Nitro-Spray (C01DA02) (n=86),Molsidomin (C01DX12) (n=66)	12798	-
Vasopressin and analogues	categorical		22053	00:20:00	Vasopressin Perf. (H01BA01) (n=19004),Vasopressin (Pitresin) (H01BA01) (n=2984),Glycylpressin (H01BA04) (n=65)	15227	-

1 Description of data cleaning of included variables

We developed a data pipeline consisting of preprocessing, merging, filtering, and postprocessing. Treatment of duplicates with identical entries and numerical values was performed first. Preprocessing methods were applied to the raw recordings and could be reused between different items. They also included datatype-specific routines for continuous and categorical variables. Merging was optional and usually needed a custom merging procedure to account for different data formats. Filtering allowed to enforce an interval or a set of allowed values. Lastly, postprocessing was applied analogously to preprocessing. The most important data cleaning procedures are summarized below.

- **Duplicates for non-medication and non-fluid variables.** We used a similar approach as (1) to treat duplicates. On a global level, we removed all recordings of a patient with identical timestamps and values. In case the values differed, we distinguished categorical and numerical variables. For categorical variables, we removed all recordings since we considered them as malformed. For numerical variables, we used the mean of all duplicates when the SD of the duplicates was <5% of the SD of the variable across all patients. Otherwise, we also removed the duplicates. After merging variables, new duplicates might occur in the recordings. For categorical variables, we handled duplicates in the custom merging procedures. For numerical variables, we used the median values.
- **Valid values for non-medications.** For most variables, an interval or set of valid values was specified (see supplement). For continuous variables, lower and upper bounds in the PDMS served as a starting point but were narrowed in some cases. For categorical variables, lists for permissible values in the PDMS were used to construct sets of valid values. We checked all values outside these sets and detected several malformed recordings, which were due to manual data entries. Whenever a valid value could be determined for those, we mapped them accordingly. Value sets were sometimes reduced to more general categories when it seemed more appropriate.
- **Valid values for medications.** The medication variables contained many artifacts which made it impractical to use dosages as variables. Instead, we only used indicators if a certain medication was administered. To this end, we removed zero and negative entries. We added three medication categories with dosages. We determined valid maximum values during the medical review of the variables and removed all entries above them.

- **Adjusting body temperature for measuring site**. Based on the measuring site, body temperature measurements were adjusted for the core temperature. We applied an offset of 0.4°C for tympanic, 0.5°C for oral and, 0.6°C for axillary sites (2). Groin and axillar show similar behavior, so an offset of 0.6°C was used for groin (3). Since no evidence could be found for the nasal site, we used the same offset as for oral.
- **Adjusted blood pressure for measuring site**. Non-invasive systolic, mean, and diastolic blood pressures were adjusted for measurement at arm or thigh. We used offsets of 7, 4, and 3 mmHg for arm and -5, 6, 11 mmHg for thigh (4).
- **Missing values.** We used value imputation only for some variables to incorporate the missingness of variables in the model. However, when only a few values were missing or missingness indicated a normal value, we imputed them. For gender, a single missing value could be derived from another source. For static variables patient class and responsible clinic, we used “inpatient” and “other”. Missing Glasgow Coma Score or Richmond Agitation-Sedation Scale indicate a normal value in the clinic, so we used them for imputation. For 29 time-series features with few missing values, we used the median value.
- **Computation of estimated glomerular filtration rate (eGFR)**. The eGFR in the PDMS was based on different formulas. We used creatinine, gender, and age to calculate it as a new variable (5).

1. Hyland SL, Faltys M, Hüser M, Lyu X, Gumbsch T, Esteban C, et al. Early prediction of circulatory failure in the intensive care unit using machine learning. Nat Med. 2020 Mar;26(3):364–73.

2. Sund-Levander M, Forsberg C, Wahren LK. Normal oral, rectal, tympanic and axillary body temperature in adult men and women: a systematic literature review. Scandinavian Journal of Caring Sciences. 2002;16(2):122–8.

3. Smith LS. Reexamining age, race, site, and thermometer type as variables affecting temperature measurement in adults – A comparison study. BMC Nursing. 2003 Jun 15;2(1):1.

4. Lakhal K, Macq C, Ehrmann S, Boulain T, Capdevila X. Noninvasive monitoring of blood pressure in the critically ill: Reliability according to the cuff site (arm, thigh, or ankle). Critical Care Medicine. 2012 Apr;40(4):1207–13.

5. Levey AS, Stevens LA, Schmid CH, Zhang Y (Lucy), Castro AF, Feldman HI, et al. A New Equation to Estimate Glomerular Filtration Rate. Ann Intern Med. 2009 May 5;150(9):604–12.

Table 3. Overview of feature classes. Every variable was assigned to one feature class and the according features were generated. Timeseries variables were assigned to classes based on their median sampling frequency. For each time series variable, five statistical quantities were generated for three time intervals. Flow and medication Feature class use the same intervals as time series low. Medication features only use an indicator if a substance was given and the number of different substances because dosage information was of insufficient quality.

Feature class	Description	Generated features (total number)
static per patient	Variables are usually collected once per patient (e.g. height).	Last value (n=1)
static per hospital stay	Variables are usually collected once per hospital stay (e.g. admission type)	Last value per hospital stay (n=1)
static per ICU stay	Variables relevant for a single ICU stay (e.g. did heart arrhythmia occur)	Last value per ICU stay (n=1)
time series high	Variables are collected repeatedly with a median sampling interval <= 15 min (e.g. heart frequency).	For 4h, 12h, 24h intervals before ICU: median, IQR, min, max, trend (n=15)
time series medium	Timeseries with a median sampling interval <= 6 h (e.g. hemoglobin lab value).	For 12h, 24h, 3d intervals before ICU discharge: median, IQR, min, max, trend (n=15)
time series low	Timeseries with a median sampling interval > 6 h (e.g. GCS score).	For 1d, 3d, 7d intervals before ICU discharge: median, IQR, min, max, trend (n=15)
flow	Variables containing a continuous flow (e.g. urine)	For 1d, 3d, 7d intervals before ICU discharge: extrapolation of daily input/output (n=3)
medication	Medication variables were assigned to WHO ATC codes and grouped according to usage.	For 1d, 3d, 7d intervals before ICU: indicator if at least one substance of a group received and number of different substances from a group (n=6)
intervention	Indicator for intervention performed during the ICU stay.	A boolean indicator if an intervention was performed and the time interval between discharge and last time it was performed (n=2)

Table 4. Features generated in addition to the standard feature classes. There were no variables in the PDMS for these features, so they were generated based on administrative data in the HIS.

**Variable name**	**Feature**
HIS data	- An indicator if current stay a 3d-ICU readmission<br>- Length of ICU stay<br>- Length of hospital stay before ICU admission<br>- Name of ICU station

2 Description of ML models for comparison

For the SAPS II model, we calculated the SAPS II for the last 24 hours of each stay and used the score as a prediction. SAPS II was developed and validated to predict mortality in the ICU based on the first 24 hours of a stay (1). However, in the included ICUs it is calculated and manually validated every day. Hence, we think it is reasonable to consider the current SAPS II for our experiments. We included the SAPS II to evaluate the potential of a validated clinical score. Due to its simplicity, we expected a rather low performance for a weak baseline. Other ICU scores exist (2). However, since the included ICUs only collected SAPS II variables consistently during the study period, we decided to use SAPS II in our experiments. Previous studies also evaluated its association with ICU readmissions (3,4).

We aimed for an LR model with similar complexity and, hence, a comparable level of interpretability as the EBM model. To this end, we performed feature selection analogously to risk function selection for EBMs. Since LR cannot handle categorical data and unknown values, we added 106 dummy variables for 39 categorical variables and 856 unknown indicators which increased the number of features from 1,423 to 2,346. This corresponds to an increase of 65% so that we aimed for 130 instead of 80 features for the LR baseline model. Feature importance for LR was determined via mean absolute feature contribution on z-normalized data. GBMs served as a strong performance baseline trained on all features. We used the widely used XGBoost library with a parallelized implementation for our experiments (5). We replaced 39 categorical variables with 106 dummy variables. An RNN was used as another strong baseline model that could incorporate raw time series data (6). Hence, the variables were used directly as inputs without generating features. For time series variables (n=152), we limited the time interval to hospital stays or at most 21 days before discharge and resampled all data to one-hour intervals to reduce the input size. A fraction of 8.23% (n=1283) stays was longer than 21 days. We padded all input series to 21 days using unknown values, imputed variables at each timestamp, forward filled unknown values. The minimum value minus one was used as an unknown indicator. For static variables, we added dummy variables for categorical variables (n=15) and unknown indicators (n=3). Parameter tuning was performed for all models (see supplement). We chose the configuration with the highest PR-AUC on the validation data. Some parameter settings caused erroneous PR-AUC values due to few recall steps in the PR curve. We filtered these models by looking at the ROC-AUC to verify whether the mode learned any useful relationships. Particularly, for RNNs this effect was strong, and we used a cutoff of 0.55 ROC-AUC to filter valid runs of parameter tuning.

1. Le Gall J-R. A New Simplified Acute Physiology Score (SAPS II) Based on a European/North American Multicenter Study. JAMA. 1993 Dec 22;270(24):2957.

2. Vincent J-L, Moreno R. Clinical review: Scoring systems in the critically ill. Critical Care. 2010 Mar 26;14(2):207.

3. Hosein FS, Bobrovitz N, Berthelot S, Zygun D, Ghali WA, Stelfox HT. A systematic review of tools for predicting severe adverse events following patient discharge from intensive care units. Critical Care. 2013 Jun 29;17(3):R102.

4. Wong EG, Parker AM, Leung DG, Brigham EP, Arbaje AI. Association of severity of illness and intensive care unit readmission: A systematic review. Heart & Lung. 2016 Jan 1;45(1):3-9.e2.

5. Chen T, Guestrin C. XGBoost: A Scalable Tree Boosting System. In: Proceedings of the 22nd ACM SIGKDD International Conference on Knowledge Discovery and Data Mining [Internet]. New York, NY, USA: Association for Computing Machinery; 2016 [cited 2021 Aug 13]. p. 785–94. (KDD ’16). Available from: https://doi.org/10.1145/2939672.2939785

6. Hochreiter S, Schmidhuber J. Long Short-Term Memory. Neural Computation. 1997 Nov;9(8):1735–80.

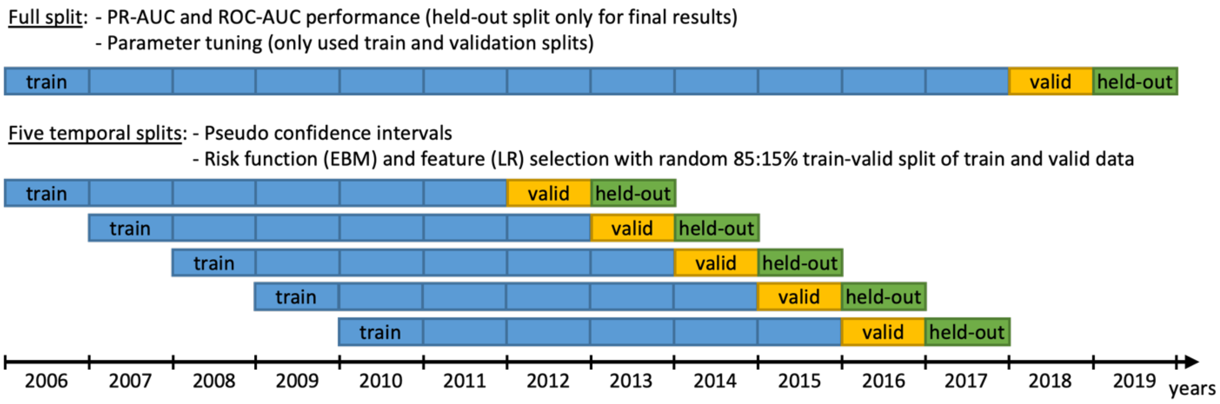

Figure 3. Data splits for the experiments. The full split utilizing all data was used to determine the performance in our experiments. We used data from 2018 and 2019 as validation (valid) and held-out sets to simulate predictions made on future data. Five additional temporal splits were used for confidence intervals and risk function (for EBM model) and feature (for LR model) selection. We optimized the performance on the validation split in our experiments. Held-out data was only used for the final results reported in the manuscript.

3 Settings used during parameter tuning of all models

3.1 Explainable Boosting Machine

Based on implementation in interpret.glassbox.ExplainableBoostingClassifier in interpret library with slight modifications enabling unknown values and exposing the argument min_samples_bin.

Table 5. Parameter settings for parameter tuning of EBM.

Parameter	Values	Comment
learning_rate	10^x for x from -12 to 1	
min_samples_leaf	2	Not tuned because determined by min_samples_bin.
max_leaves	2, 4, 6, 8, 10	
interactions	0	
min_samples_bin	100, 150, 200, 250, 300, 350, 400	
binning	quantile	
max_rounds	5000	
outer_bags	8	The default value, which was a good trade-off between robustness and computation time.
random_state	87	Project-wide seed determined a-priori.
max_interaction_bins	4, 6, 8, 10, 12, 14	Considered 2D training for at most 20 iterations.

Best parameters for all features:

learning_rate: 0.01, max_leaves: 8, min_samples_bin: 200

Best parameters for EBM with limited size (80 1D risk function, 5 2D risk functions):

learning_rate: 0.1, max_leaves: 4, min_samples_bin: 200, max_interaction_bins: 4

3.2 Logistic Regression

Based on implementation in sklearn.linear_model.LogisticRegression in scikit-learn library.

Table 6. Parameter settings for parameter tuning of LR model.

Parameter	Values	Comment
C	2^x for x from -20 to 3	Taken from https://www.csie.ntu.edu.tw/~cjlin/papers/liblinear.pdf and refined by additional experiments.
penalty	l1, l2	
tol	0.1, 0.01, 0.001, 0.0001, 0.00001	
solver	liblinear	
max_iter	10, 100, 500	
random_state	87	Project-wide seed determined a-priori.

Best parameters for all features:

C: 0.125, penalty: ‘l1’, tol: 0.01, max_iter: 100 (same results for 500)

Best parameters for LR model with limited size (130 features):

C: 1, penalty: ‘l1’, tol: 0.1, max_iter: 100 (same results for 10, 500)

3.3 Gradient Boosting Machine (XGBoost)

Based on implementation in xgboost.sklearn.XGBModel in xgboost library.

Table 7. Parameter settings for parameter tuning of GBM model.

Parameter	Values	Comment
learning_rate	x*0.02 for x from 1 to 10	
min_child_weight	1, 3, 5, 7, 9	
max_depth	1, 2, 3, 4, 5	Already observed overfitting for 4-5 so used in as maximum.
objective	binary:logistic	
n_estimators	100, 500	

eval_metric	aucpr	
use_label_encoder	False	Hide deprecation warning.
seed	87	Project-wide seed determined a-priori.

Best parameters for all features:

learning_rate: 0.02, min_child_weight: 7, max_depth: 3, n_estimators: 500

3.4 Recurrent Neural Network with Long Short-Term Memory

Based on implementation in tensorflow.keras.Model in tensorflow library.

Table 8. Parameter settings for parameter tuning of RNN model.

**Parameter**	**Values**	**Comment**
lstm_neurons	32, 64	RNN layers for time series data.
dense_neurons	64, 128	Fully connected layers for RNN output and static variables.
dropout	0, 0.1, 0.2, 0.3, 0.4, 0.5	
recurrent_dropout	0	
learning_rate	0.1, 0.01, 0.001, 0.0001, 0.00001	
batch_size	32	
rnn_layers	1	The number of RNN layers. Preliminary experiments with more layers showed no benefit.
epochs	1, 2, 3, 4, 5, 6, 7, 8, 9, 10	
seed	87	Project-wide seed determined a-priori.

Best parameters for all features:

lstm_neurons: 64, dense_neurons: 64, dropout: 0.4, learning_rate: 0.001, epochs: 3

4 Qualitative feedback during EBM model inspection

General comments

1. Most risk functions exhibited one of the listed problems to some extent. However, often the effect should not be large. Hence, we agreed to only exclude risk functions when a problem was clearly fulfilled and there is a considerable impact for patients.
2. Small peaks and drops of the risk function were often ignored. Especially, when they occurred in a sparse region where probably only a few patients would be affected (e.g. 7, 23).
3. Outlier values often received inexplicable risk values, however, since only a few patients would be affected or since they are caused by erroneous data they were often ignored (e.g. 42).
4. It is unusual in clinical practice to consider only isolated variables or pairs, so this information was sometimes not sufficient for a reasonable decision (e.g. 66).
5. It was difficult to interpret a feature over the complete cohort because sub-cohorts behaved or were treated very differently (e.g. 13). For instance, features over long time intervals contain patients with very short and long stays.
6. Feature values did depend on the measuring frequency which could differ between patients and was unknown during risk function inspection (e.g. 62).
7. The severity of surgery probably acted as a confounder for some variables. However, adding surgery as an explicit variable did not change the behavior, so there was probably a more complicated relationship (e.g. in 31 increase of CK as a sign of the severity of the surgery).
8. It was difficult to consider the situation at discharge because many variables were also used routinely during the ICU stay. Also, different clinical paths could lead to the same situation at discharge and it was hard to incorporate them all.
9. The team tended to construct an explanation for each risk function even though it is very hard to find evidence for different explanations (e.g. 8, 17).
10. It was hard to interpret risk for values outside the usual value ranges and variables that were seldomly used in clinical practice (e.g. 8, 15, 59).
11. There was limited intuition for IQR and trend features and it was very hard to mentally switch between the different quantities during inspection (e.g. 36, 62). Also, small values for trend features were hard to interpret (e.g. 20).
12. Even though the content of the 2D risk function could be understood it was very hard to determine its clinical relevance. Often variables were combined that in practice are not interpreted together and have no obvious connection (1-5).
13. When a risk function was less interpretable there was a tendency to rely more on the model to find useful relationships and the risk function was included.

Factors hindering interpretability

14. Fluctuating risk functions that rapidly switch between positive and negative values made it hard to interpret them and to determine their effect (e.g. 20, 30, 47, 56).
15. It was difficult to interpret the number of cases in a bin because bin sizes differed (e.g. in 6, 12 histograms suggested even data distribution).
16. Bins were sometimes too large and included abnormal and normal values, so the team was unable to distinguish them (e.g. 30).

17. Minimum and maximum features were more susceptible to outlier values (e.g. 30).

Factors supporting interpretability

18. It was helpful to see the data distribution as a sanity check to verify the item distribution and see if it was collected as expected.

5 Cohort selection and data processing for external validation with MIMIC-IV

We performed an external validation of the EBM and the GBM model on the MIMIC-IV database version 1.0 (1,2). MIMIC-IV consists of 76,540 stays of 53,150 patients admitted to an ICU at BIDMC between 2008-2019. It contains all data collected at the ICUs making it a good candidate for external validation. We used code from the shared code repository to load the data and generate medical concepts.

We tried to mimic the cohort selection as close as possible. MIMIC-IV already provided consecutive ICU stays as so-called concepts so that merging of transfer was not necessary. Especially, the manual procedure to classify gaps between ICU stays was irrelevant for MIMIC-IV. The flow chart in the supplement shows the cohort selection. First, 104 malformed ICU stays of 76 patients were excluded due to hospital discharges before admission, overlapping hospital stays, and a time of death before ICU admission. Second, stays that were not discharged from an ICU managed by the Department of Anesthesia, Critical Care, and Pain Medicine at BIDMC were excluded (n=43,154). The included ICUs were Trauma Surgical Intensive Care Unit, Surgical Intensive Care Unit, Cardiovascular Intensive Care Unit, and Neuroscience Intensive Care Unit. Next, we excluded all stays who died during the ICU stay (n=1,888). This included two stays that were transferred to an ICU after death which we also considered as death at ICU. Analogously to the original cohort, we required a length of stay at the hospital after the ICU of at least 72 hours (n=12,187). The ratio of the excluded cases was considerably higher than in the original cohort. Lastly, we removed cases that had no heart frequency entries for at least two hours (n=99).

We also labeled discharges from an included ICU to standard care as true when a patient was readmitted to any ICU or IMC units or died within three days. For the UKM cohort, we designed a special procedure and performed manual annotation for transfers of at most twelve hours. However, this was not feasible for the MIMIC-IV cohort because we lacked clinical knowledge about the data. Hence, we used a simplified procedure that required stays at a standard care unit or consecutive readmission to an ICU or IMC unit to last at least one hour to prevent artifacts. Also, we excluded the post-anesthesia care unit and unknown from the standard care units because they could indicate a planned surgery leading to readmission to an ICU or IMC unit. BIDMC had more IMC units so that fewer ICU stays were directly discharged to a standard care unit. Hence, we expected a lower fraction of positive labels. However, 1626 ICU stays were labeled as readmission or death. Of those, 1273 were readmitted to an ICU, 39 to an IMC unit, and 314 died within three days. We controlled 20 random positive labels stratified by ICUs to verify the labeling procedure.

We extracted 41 variables for the EBM model from MIMIC-IV. We also used the data collected during ICU stays. We searched through the item definitions and the provided code to identify relevant items. Analogously to the UKM cohort we defined allowed value ranges and applied median value imputation. Only the variable procalcitonin was not contained in MIMIC-IV. As a result, 66 features were generated for the EBM and 515

features for the GBM model. The same code was used for feature generation. Less effort was put into data cleaning compared to the UKM cohort. However, we expected that the MIMIC-IV database has better data quality data since it is publicly available, and the developers integrated the feedback of several researchers.

Variables were collected differently in the MIMIC cohort. For instance, blood loss was collected less frequently. Hence, we deemed it necessary to retrain the EBM model for this task. We used the same parameter setting determined on the UKM cohort. For a proper performance comparison, we resampled negative cases in the MIMIC-IV cohort to obtain the same ratio of positive cases. MIMIC-IV only provides intervals of three years for each ICU stay to protect the patient's privacy. Hence, it was impossible to use the same temporal splits. Instead, we used data between 2008 until 2016 as training set and data from 2017 to 2019 as held-out split. For confidence intervals, we used five random 85:15% splits of the data between 2008 and 2016 for training and testing.

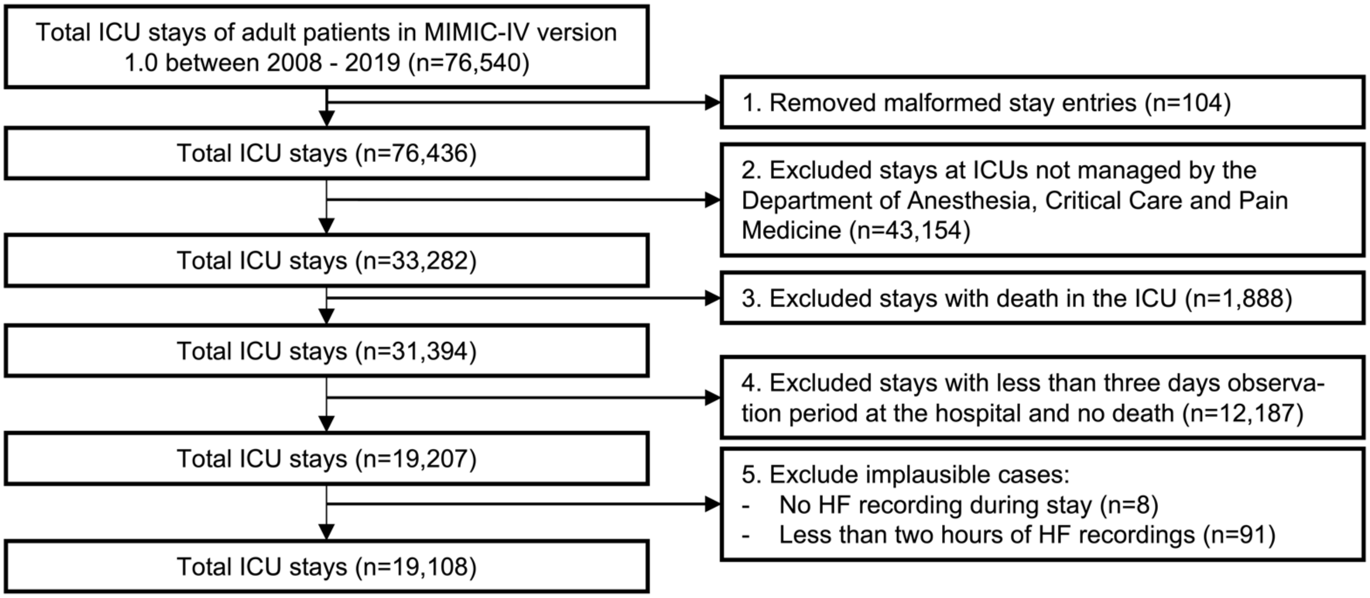

Figure 4. Cohort selection for the MIMIC cohort. We tried to apply the same selection procedure as for the UKM cohort. Merging was not necessary since MIMIC-IV already contained consecutive ICU stays.

Table 9. Overview of the MIMIC cohort.

Characteristic	All ICU stays	No 3-day readmission or death after ICU discharge	3-day readmissions or death after ICU discharge
Number of stays	19,108 (100.0%)	17,482 (91.5%)	1626 (8.5%)
Number of patients	17,499 (100.0%)	16,591 (94.8%)	1525 (8.7%)
Mean age (SD)	65.16 (15.86)	64.81 (15.86)	68.97 (15.34)
Gender female	7681 (100.0%)	6971 (90.8%)	710 (9.2%)
Gender male	11,427 (100.0%)	10,511 (92.0%)	916 (8.0%)
Mean length of ICU stay (SD)	3.52 days (4.69 days)	3.47 days (4.63 days)	4.05 days (5.26 days)
ICU at discharge	Cardiac Vascular Intensive Care Unit (n=6891) Surgical Intensive Care Unit (n=6397)<br><br>Trauma SICU (n=5291) Neuro Surgical Intensive Care Unit (n=529)	Cardiac Vascular Intensive Care Unit (n=6523) Surgical Intensive Care Unit (n=5778)<br><br>Trauma SICU (n=4744) Neuro Surgical Intensive Care Unit (n=437)	Cardiac Vascular Intensive Care Unit (n=368)<br><br>Surgical Intensive Care Unit (n=619)<br><br>Trauma SICU (n=547) Neuro Surgical Intensive Care Unit (n=92)

1. Johnson, Alistair, Bulgarelli, Lucas, Pollard, Tom, Horng, Steven, Celi, Leo Anthony, Mark, Roger. MIMIC-IV [Internet]. PhysioNet; [cited 2021 Aug 23]. Available from: https://physionet.org/content/mimiciv/1.0/

2. Goldberger AL, Amaral LAN, Glass L, Hausdorff JM, Ivanov PCh, Mark RG, et al. PhysioBank, PhysioToolkit, and PhysioNet: Components of a New Research Resource for Complex Physiologic Signals. Circulation [Internet]. 2000 Jun 13 [cited 2021 Aug 23];101(23). Available from: https://www.ahajournals.org/doi/10.1161/01.CIR.101.23.e215

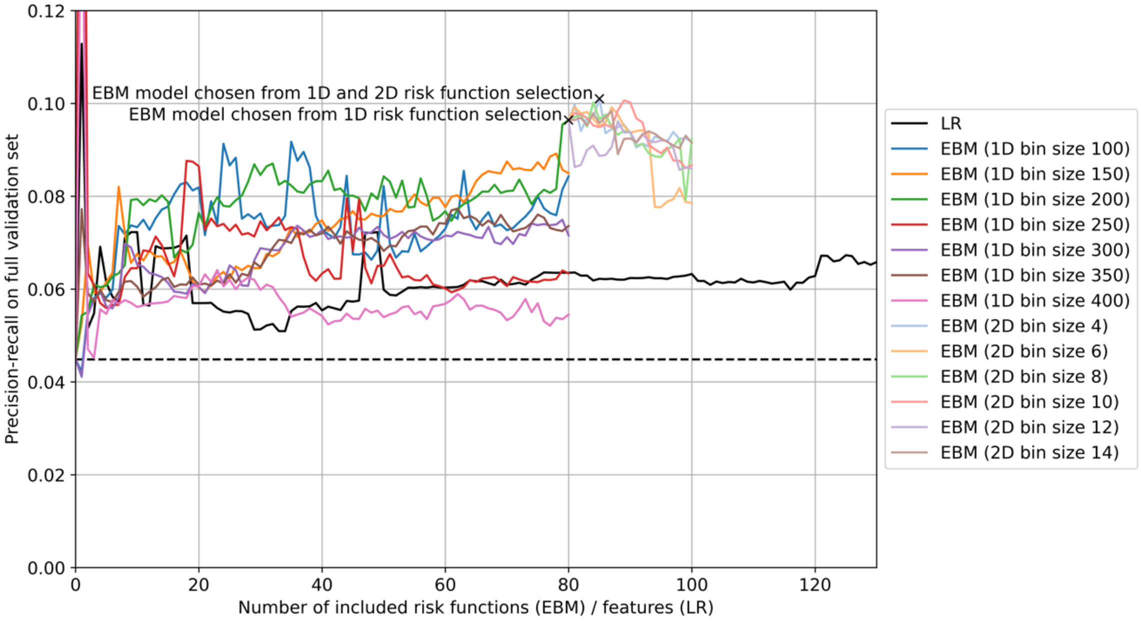

Figure 5. Performance during risk function selection for EBM and feature selection for LR models.
