# Supplementary material for: Development and validation of an interpretable 3 day intensive care unit readmission prediction model using explainable boosting machines
Source: Front Med (Lausanne). 2022 Aug 23;9:960296. doi: 10.3389/fmed.2022.960296 (PMC9445989; doi:10.3389/fmed.2022.960296)
Supplement: Supplementary file 3 [file Data_Sheet_3.PDF]

Risk functions of the final EBM model

1. Age (static all data) [years] x BE (iqr 3d) [mmol/L]

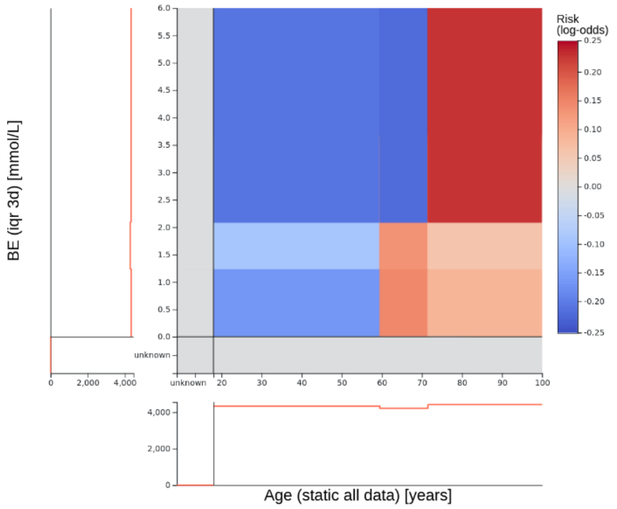

Relative importance: 4.20%

Applicable exclusion criteria: 4

Notes: -

Decision: 3

2. Drugs for constipation (unique 1d) x Leucocytes (median 1d) [thousand/µL]

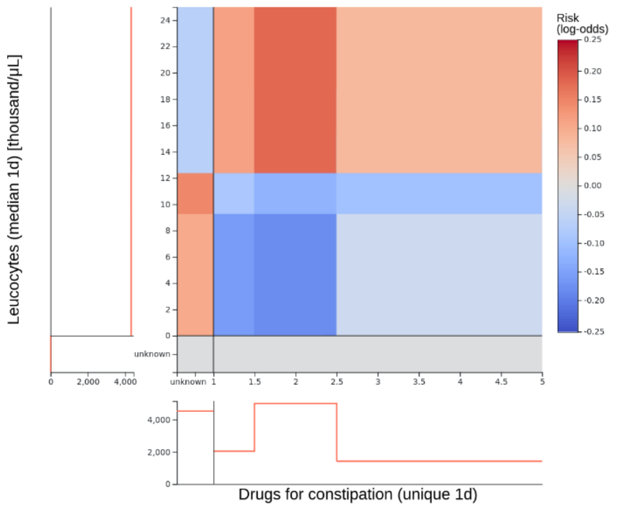

Relative importance: 3.52%

Applicable exclusion criteria: 4

Notes: -

Decision: 3

3. Blood volume out (extrapolate 7d) [mL] x Procalcitonin (max 7d) [ng/mL]

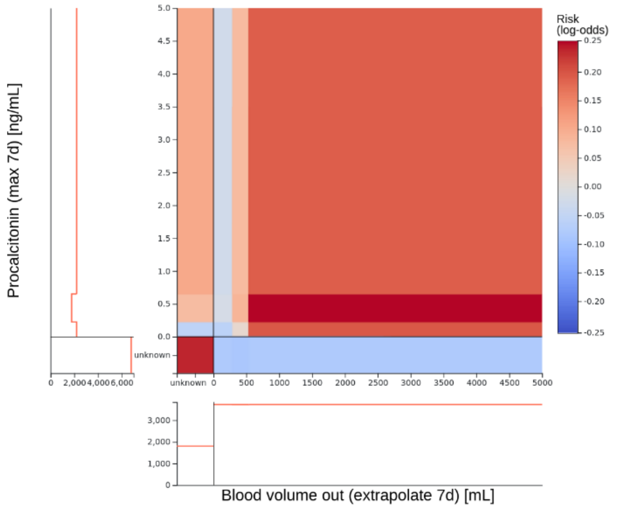

Relative importance: 2.57%

Applicable exclusion criteria: 4

Notes: -

Decision: 3

4. Hematocrit (max 3d) [%] x Blood volume out (extrapolate 3d) [mL]

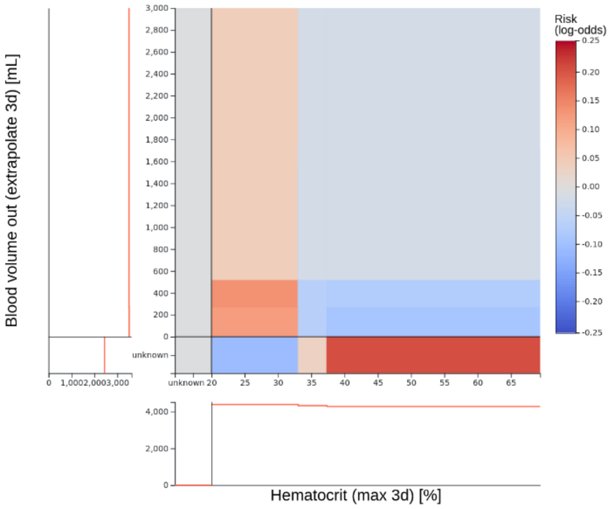

Relative importance: 2.19%

Applicable exclusion criteria: 4

Notes: -

Decision: 3

5. Leucocytes (median 1d) [thousand/µL] x Blood volume out (extrapolate 3d) [mL]

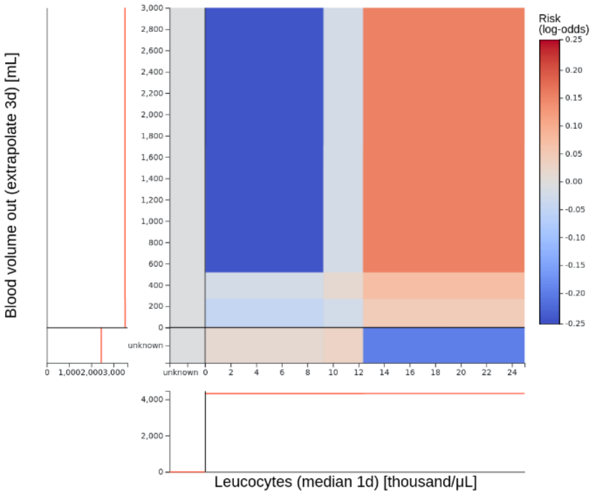

Relative importance: 1.87%

Applicable exclusion criteria: 4

Notes: -

Decision: 3

6. Tubus exists (days since last application per icu stay)

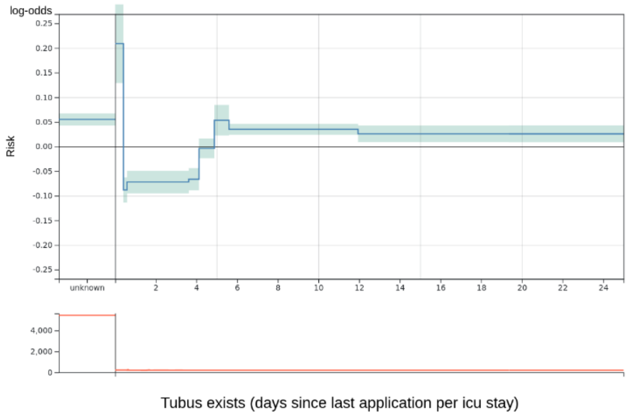

Relative importance: 1.71%

Applicable exclusion criteria: -

Notes:

- Decreased risk between 0.416 and 4.130 might be due to surgical patients that are extubated as planned

Decision: 1

7. Age (static all data) [years]

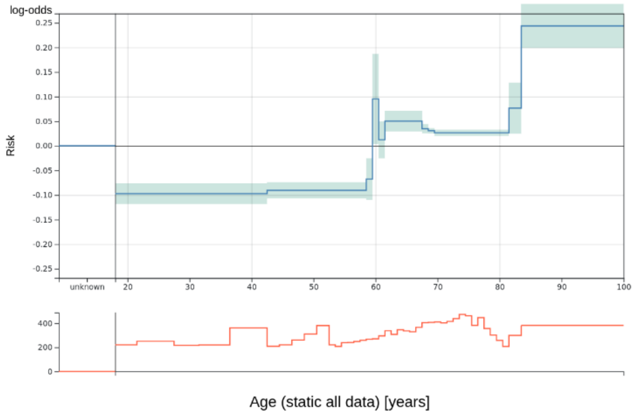

Relative importance: 1.70%

Applicable exclusion criteria: 3

Notes:

- Effect of peak at 60 considered as negligible

Decision: 2

8. Antithrombotic agents prophylactic dosage (days since last appl. per icu stay)

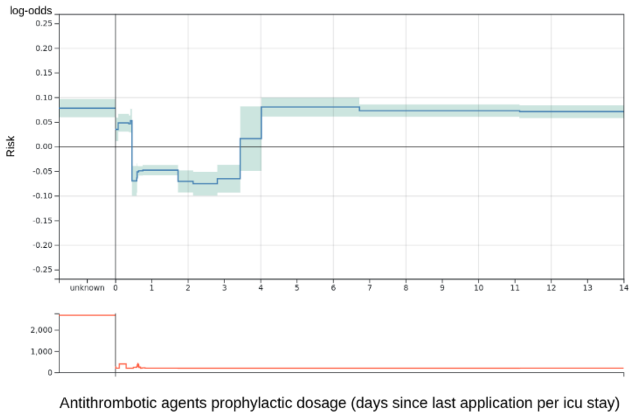

Relative importance: 1.65%

Applicable exclusion criteria: -

Notes:

- Difficult to determine patient cohorts responsible for different interval
- Information about therapeutic dosage would be helpful, but not included in the model

Decision: 1

9. PTT (max 1d) [s]

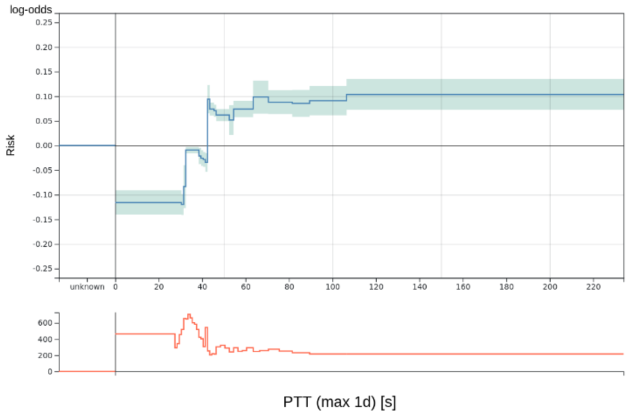

Relative importance: 1.63%

Applicable exclusion criteria: 2

Notes:

- Practice of measuring PTT changed in 2019, which cannot be corrected easily.

Decision: 3

10. O2 saturation (min 12h) [%]

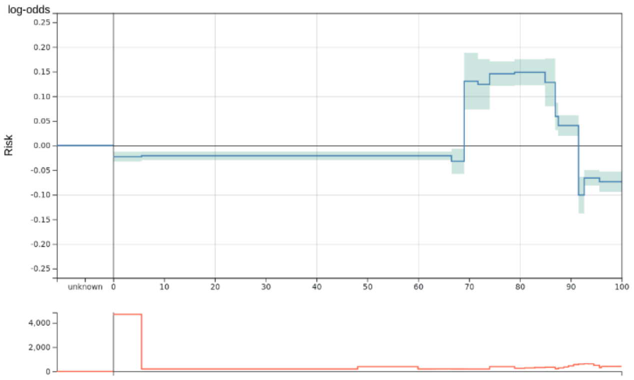

O2 saturation (min 12h) [%]

Relative importance: 1.58%

Applicable exclusion criteria: -

Notes: -

Decision: 1

11. Blood volume out (extrapolate 7d) [mL]

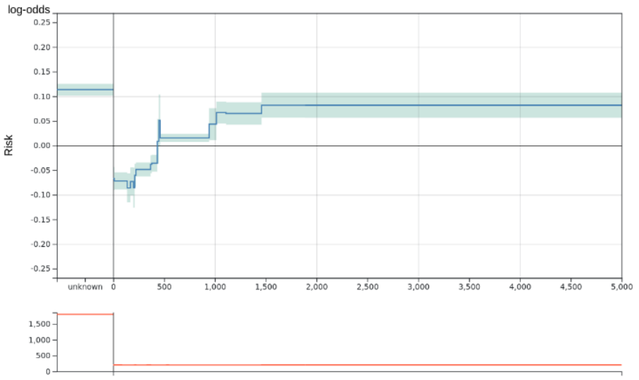

Blood volume out (extrapolate 7d) [mL]

Relative importance: 1.52%

Applicable exclusion criteria: -

Notes:

- Effect of peak at 450 ml considered as negligible

Decision: 1

12. Gamma-GT (median 7d) [U/L]

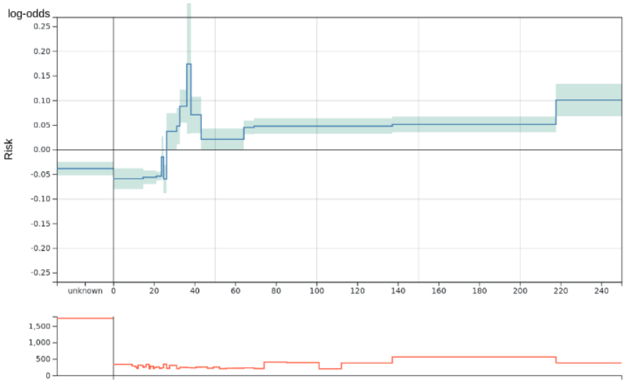

Gamma-GT (median 7d) [U/L]

Relative importance: 1.46%

Applicable exclusion criteria: -

Notes:

- Higher risk between 26.25 and 43.25 probably due to medical patients (i.e. no surgery)

Decision: 1

13. Chloride (trend per day 3d) [mmol/L]

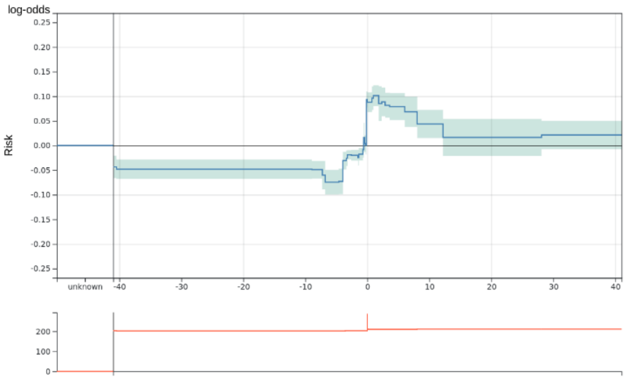

Chloride (trend per day 3d) [mmol/L]

Relative importance: 1.40%

Applicable exclusion criteria: -

Notes:

- Mixed cohort of hyper- and hypochloremia making it hard to determine a general trend
- More hyperchloremia patients, so that negative trend better

Decision: 1

14. Heart rate (min 4h) [bpm]

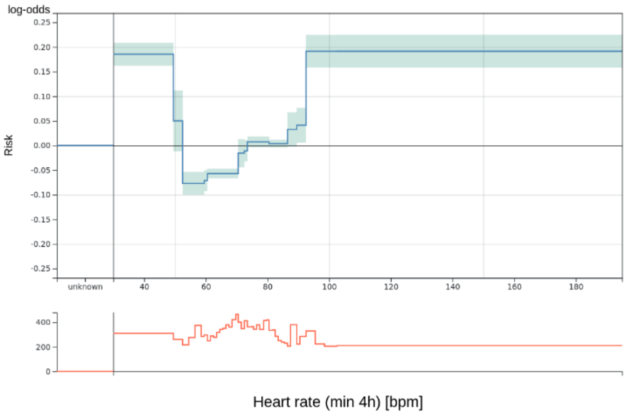

Relative importance: 1.39%

Applicable exclusion criteria: -

Notes: -

Decision: 1

15. PTT (max 3d) [s]

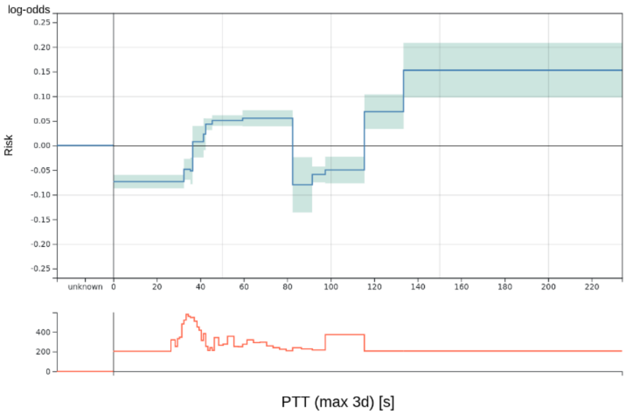

Relative importance: 1.37%

Applicable exclusion criteria: 2

Notes:

- Practice of measuring PTT changed in 2019, which cannot be corrected easily

Decision: 3

16. Chloride (min 1d) [mmol/L]

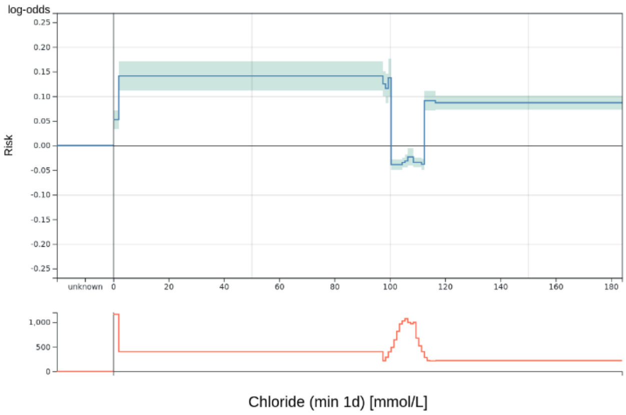

Relative importance: 1.37%

Applicable exclusion criteria: -

Notes: -

Decision: 1

17. Hemoglobin (max 3d) [mmol/L]

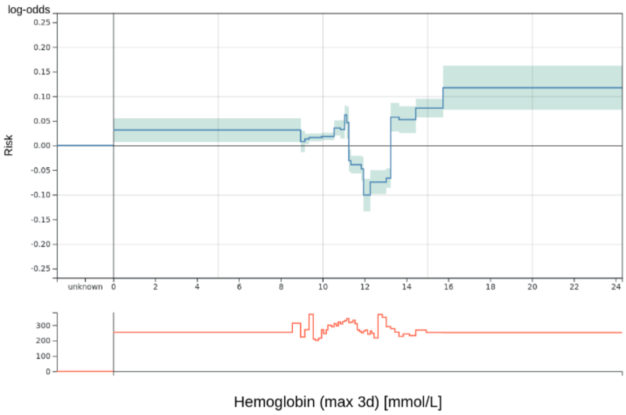

Relative importance: 1.30%

Applicable exclusion criteria: -

Notes: -

Decision: 1

18. Length of stay before ICU [days]

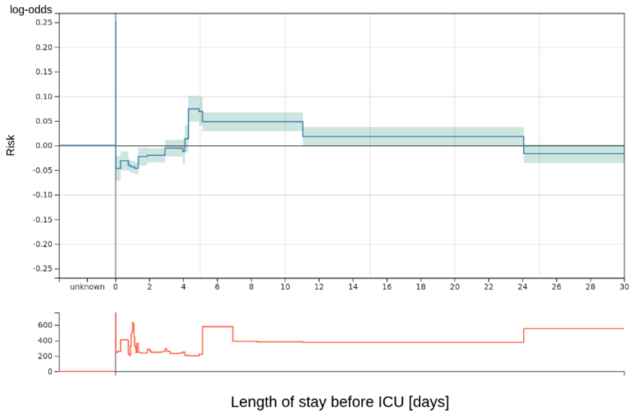

Relative importance: 1.28%

Applicable exclusion criteria: -

Notes:

- Difficult to interpret for patients with repeated ICU stays where this quantity is high.

Decision: 1

19. Hematocrit (max 3d) [%]

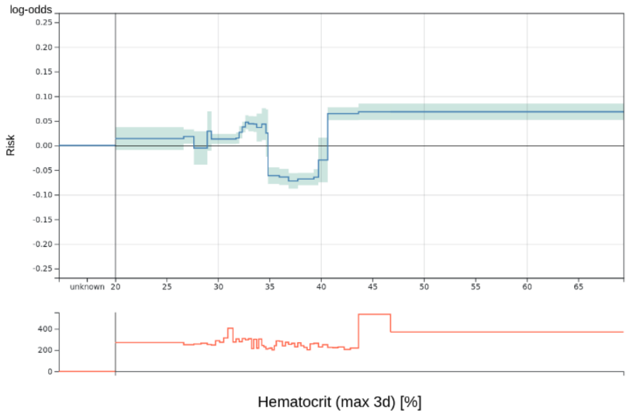

Relative importance: 1.26%

Applicable exclusion criteria: -

Notes: -

Decision: 1

20. Calcium (trend per day 3d) [mmol/L]

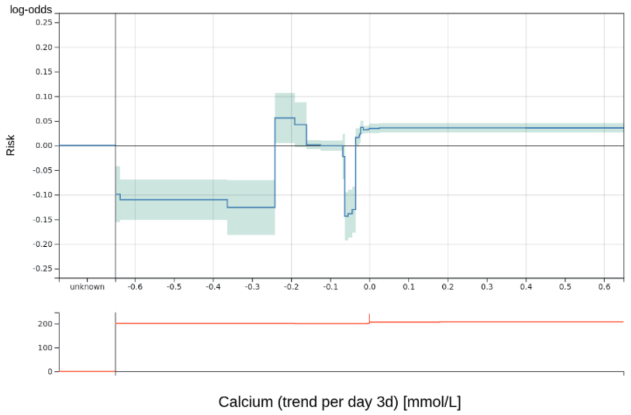

Relative importance: 1.26%

Applicable exclusion criteria: 3, 4

Notes: -

Decision: 3

21. eGFR (trend per day 7d) [L]

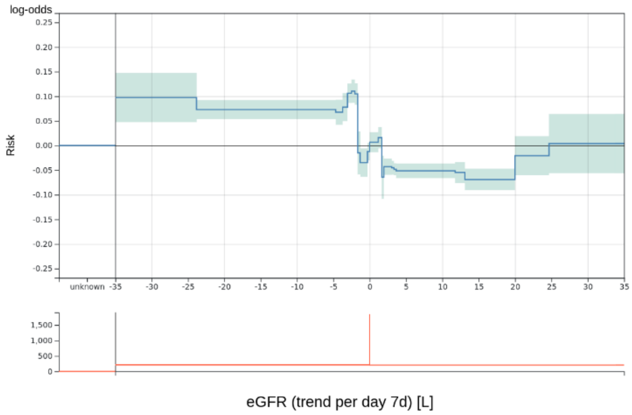

Relative importance: 1.24%

Applicable exclusion criteria: -

Notes: -

Decision: 1

22. RAS scale (max 3d)

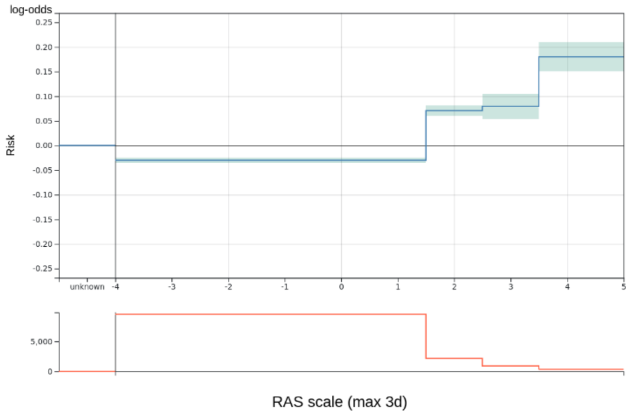

Relative importance: 1.24%

Applicable exclusion criteria: -

Notes: -

Decision: 1

23. Urine volume out (extrapolate 1d) [mL]

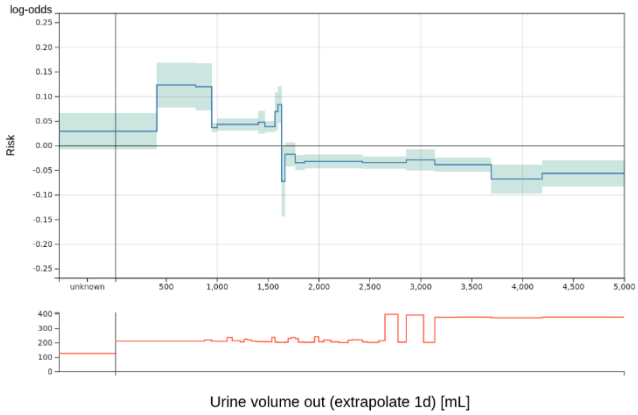

Relative importance: 1.24%

Applicable exclusion criteria: -

Notes:

- Peaks at 1600 and 1650 considered as irrelevant

Decision: 1

24. Thrombocytes (trend per day 7d) [thousand/μL]

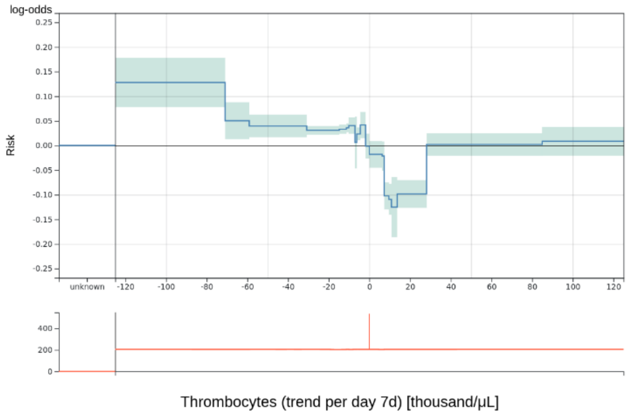

Relative importance: 1.24%

Applicable exclusion criteria: -

Notes: -

Decision: 1

25. Blood volume out (extrapolate 3d) [mL]

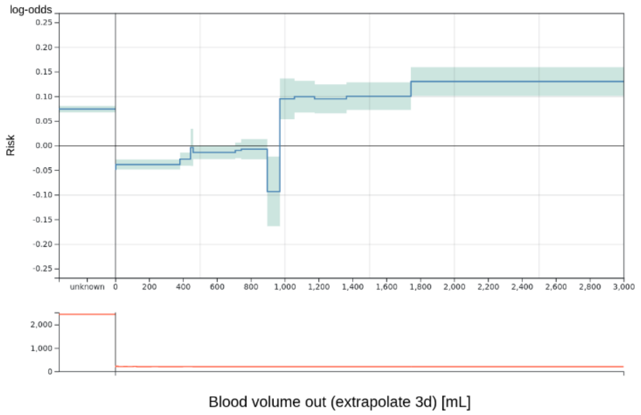

Relative importance: 1.23%

Applicable exclusion criteria: 3

Notes:

- No medical explanation for drop at 900.

Decision: 2

26. paO2/FiO2 (median 1d) [mmHg/FiO2]

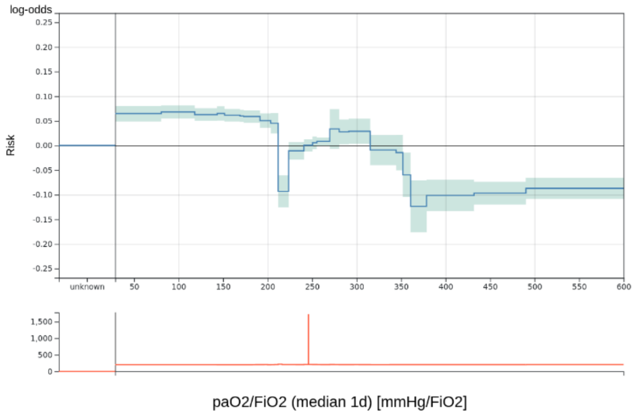

Relative importance: 1.21%

Applicable exclusion criteria: 2

Notes:

- Drop at 215 because of venous blood gas analyses.

Decision: 2

27. pH (trend per day 3d)

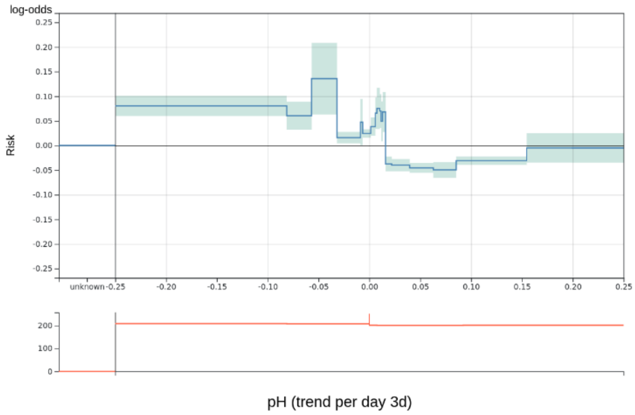

Relative importance: 1.21%

Applicable exclusion criteria: -

Notes: -

Decision: 1

28. Phosphate (min 7d) [mg/dL]

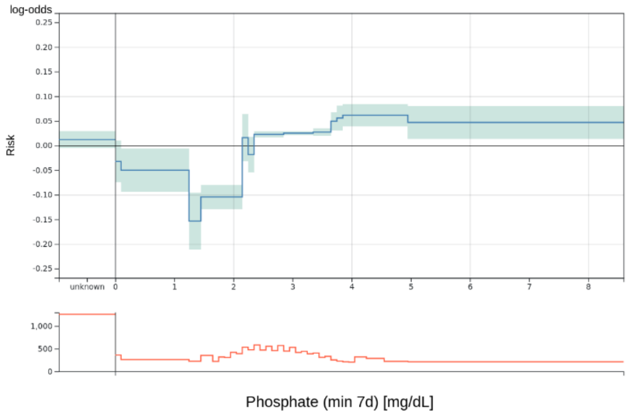

Relative importance: 1.20%

Applicable exclusion criteria: 3

Notes:

- Increasing risk for values larger than 2.35 might be due to cohort of ICU discharges.

Decision: 2

29. pH (median 1d)

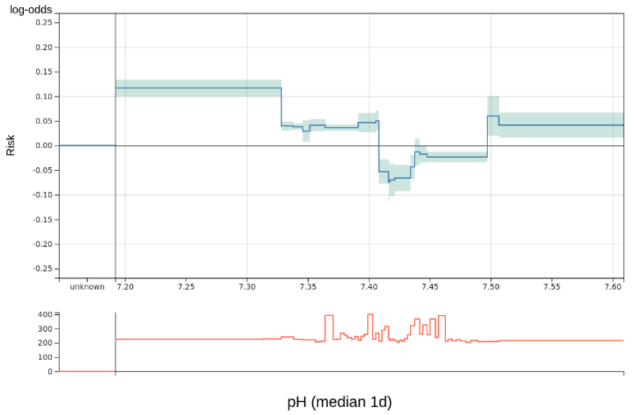

Relative importance: 1.20%

Applicable exclusion criteria: -

Notes: -

Decision: 1

30. Body core temperature (min 1d) [°C]

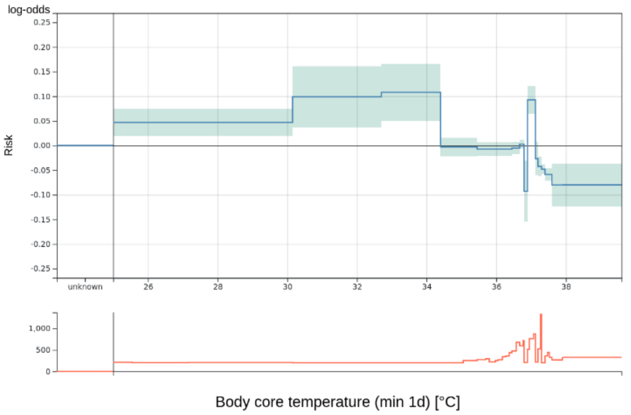

Relative importance: 1.18%

Applicable exclusion criteria: 3

Notes:

- Strong fluctuation for similar value ranges.
- Maximum bin goes from 37.9 to 39.6 which should behave differently

Decision: 3

31. CK (min 7d) [U/L]

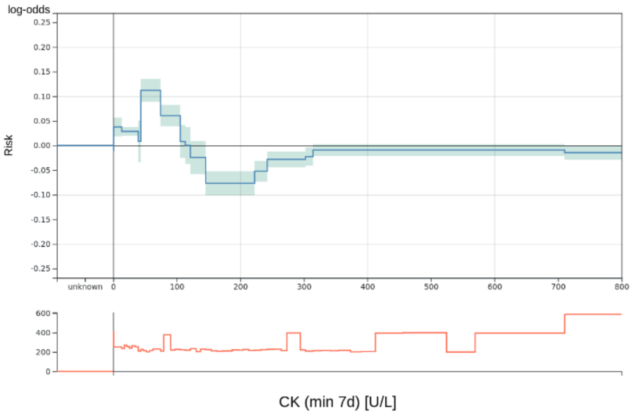

Relative importance: 1.15%

Applicable exclusion criteria: -

Notes:

- Severity of a surgery probably a confounder for this variable.

Decision: 1

32. RAS scale (trend per day 12h)

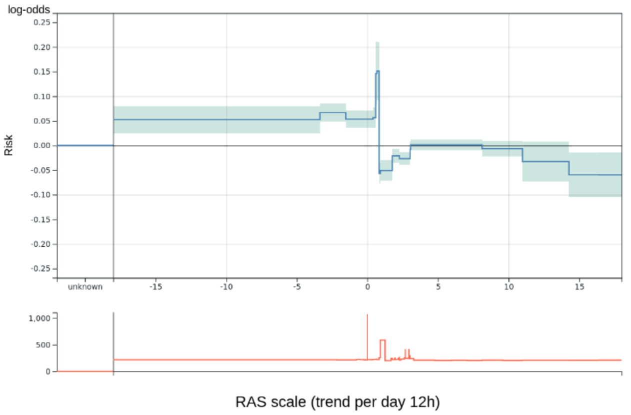

Relative importance: 1.13%

Applicable exclusion criteria: 2, 4

Notes:

- Trend of categorical variable not meaningful.

Decision: 3

33. Potassium (median 1d) [mmol/L]

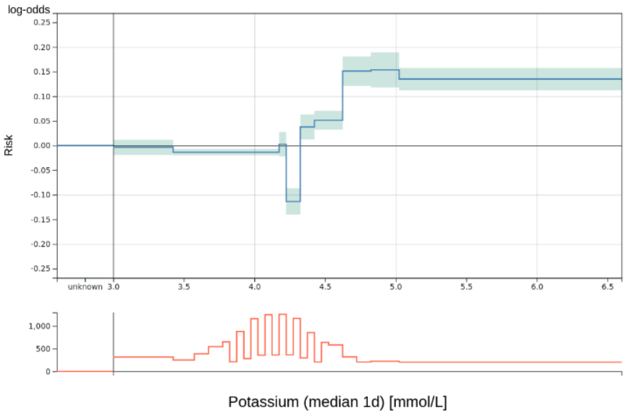

Relative importance: 1.13%

Applicable exclusion criteria: -

Notes:

- Drop at 4.25 probably due to cardiac surgery patients that have this as a target value.

Decision: 1

34. GCS score (min 3d)

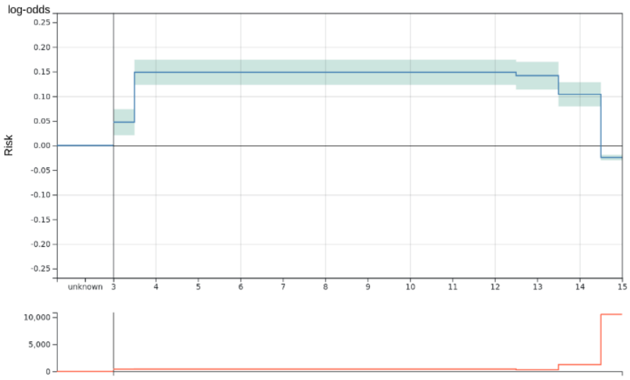

GCS score (min 3d)

Relative importance: 1.11%

Applicable exclusion criteria: -

Notes:

- Lower risk for GCS 3 probably due to sedated patients after surgery.

Decision: 1

35. Body core temperature (median 1d) [°C]

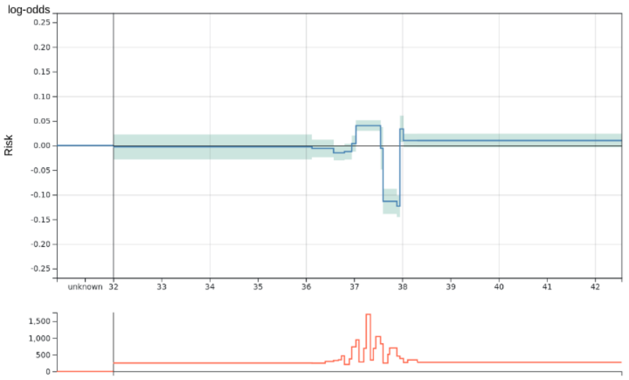

Body core temperature (median 1d) [°C]

Relative importance: 1.10%

Applicable exclusion criteria: -

Notes: -

Decision: 1

36. BE (iqr 3d) [mmol/L]

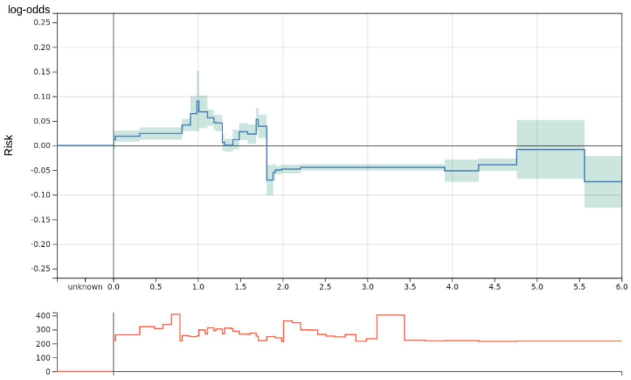

BE (iqr 3d) [mmol/L]

Relative importance: 1.10%

Applicable exclusion criteria: 3, 4

Notes:

- Low risk for large values against medical practice.

Decision: 3

37. Blood Urea Nitrogen (min 3d) [mg/dL]

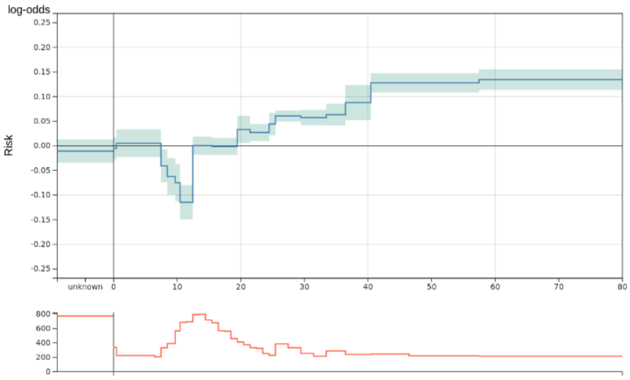

Blood Urea Nitrogen (min 3d) [mg/dL]

Relative importance: 1.10%

Applicable exclusion criteria: -

Notes: -

Decision: 1

38. paO2/FiO2 (trend per day 3d) [mmHg/FiO2]

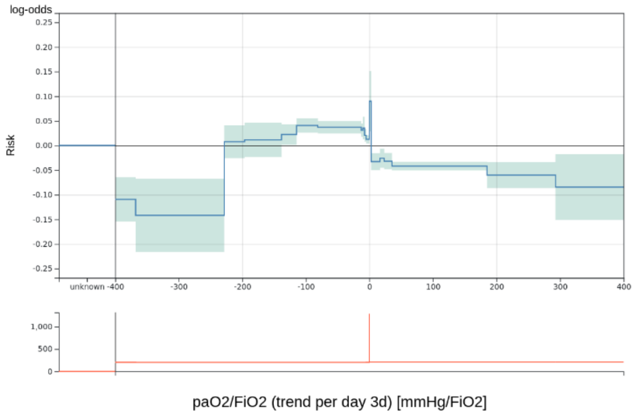

Relative importance: 1.09%

Applicable exclusion criteria: 4

Notes: -

Decision: 2

39. Drugs for constipation (unique 1d)

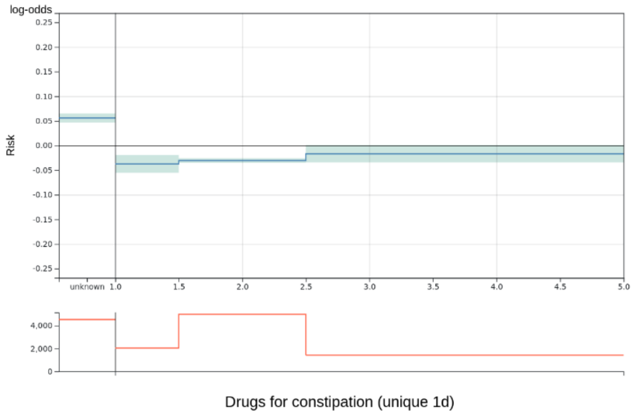

Relative importance: 1.09%

Applicable exclusion criteria: -

Notes: -

Decision: 1

40. Urine volume out (extrapolate 7d) [mL]

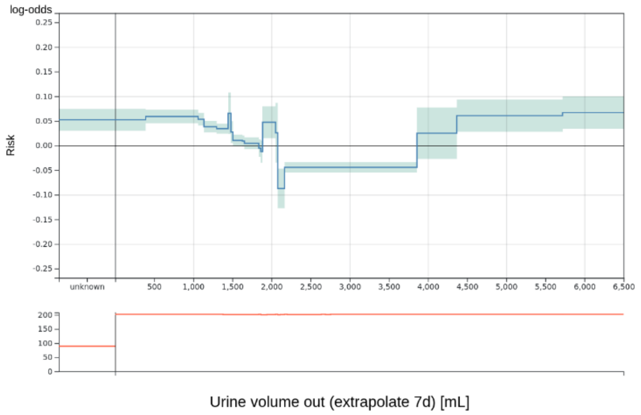

Relative importance: 1.09%

Applicable exclusion criteria: -

Notes: -

Decision: 1

41. PTT (min 7d) [s]

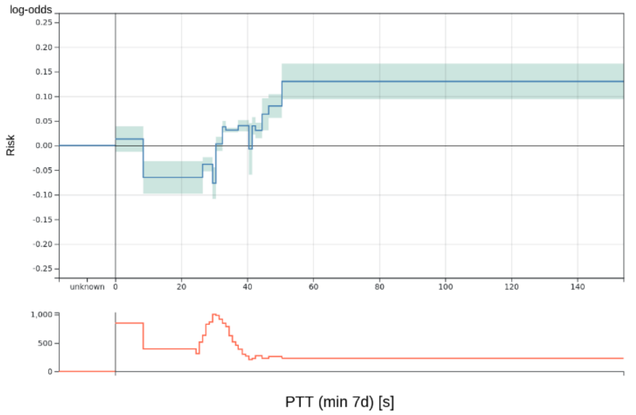

Relative importance: 1.07%

Applicable exclusion criteria: 2

Notes:

- Practice of measuring PTT changed in 2019, which cannot be corrected easily.

Decision: 3

42. Diastolic blood pressure (median 1d) [mmHg]

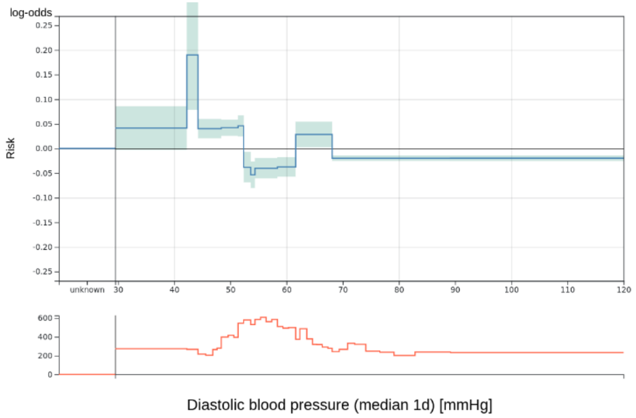

Relative importance: 1.06%

Applicable exclusion criteria: 3

Notes:

- Peak at 43 considered problematic.

Decision: 2

43. pO2 (min 12h) [mmHg]

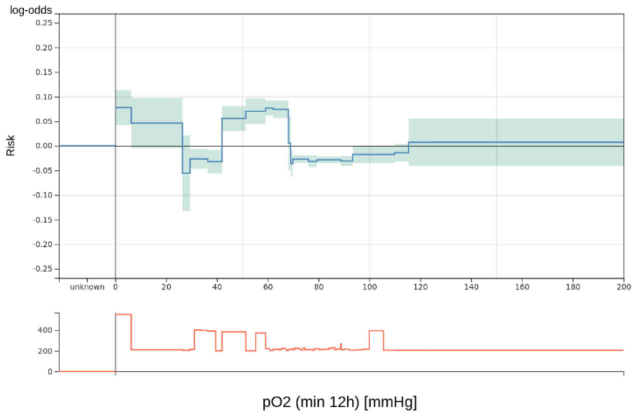

Relative importance: 1.06%

Applicable exclusion criteria: 2

Notes:

- Drop at 26.45 to 42.05 because of venous blood gas analyses.

Decision: 2

44. CK-MB (max 3d) [U/L]

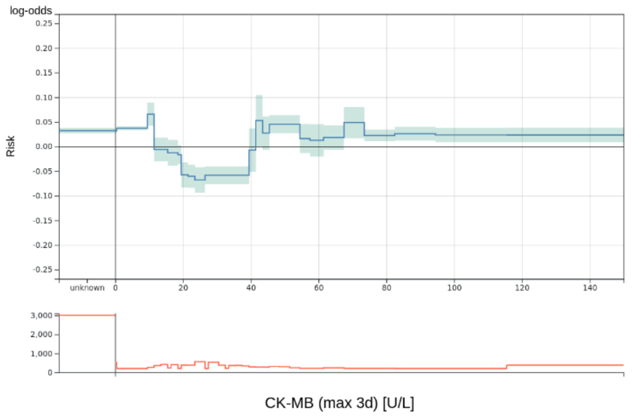

Relative importance: 1.05%

Applicable exclusion criteria: -

Notes: -

Decision: 1

45. RAS scale (max 1d)

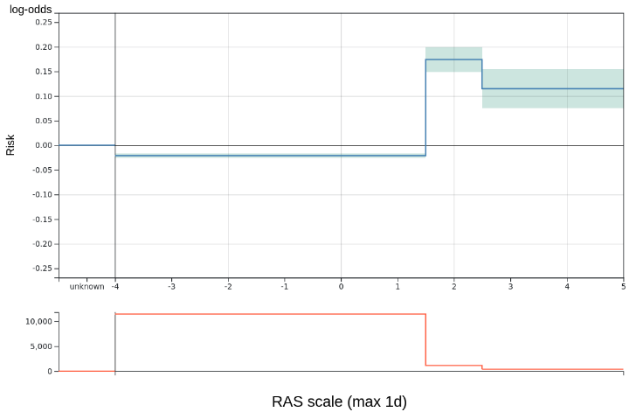

Relative importance: 1.05%

Applicable exclusion criteria: -

Notes: -

Decision: 1

46. PTT (min 3d) [s]

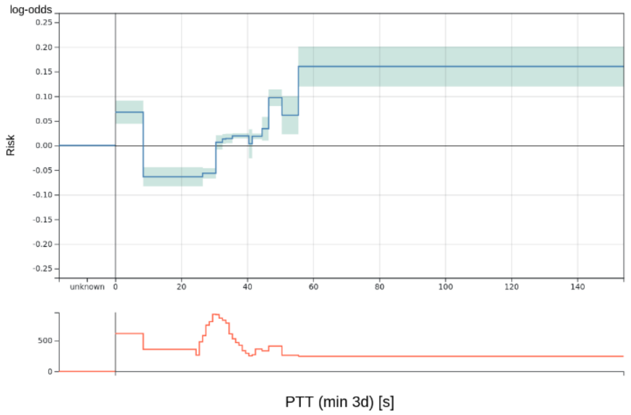

Relative importance: 1.05%

Applicable exclusion criteria: 2

Notes:

- Practice of measuring PTT changed in 2019, which cannot be corrected easily.

Decision: 3

47. Systolic blood pressure (iqr 12h) [mmHg]

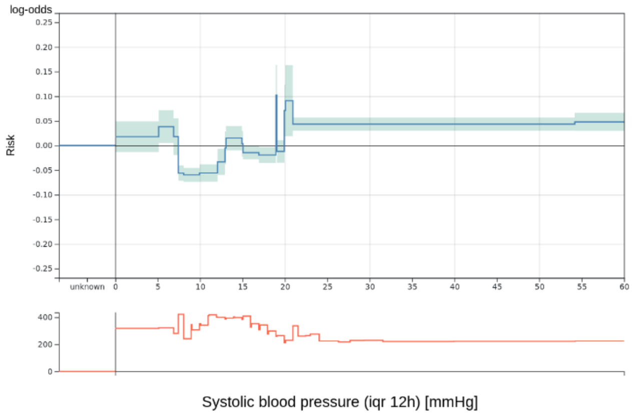

Relative importance: 1.05%

Applicable exclusion criteria: 4

Notes: -

Decision: 2

48. paO2/FiO2 (median 3d) [mmHg/FiO2]

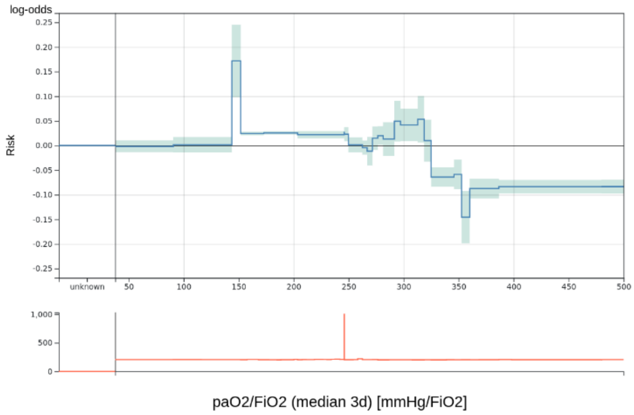

Relative importance: 1.04%

Applicable exclusion criteria: -

Notes: -

Decision: 1

49. CK (median 7d) [U/L]

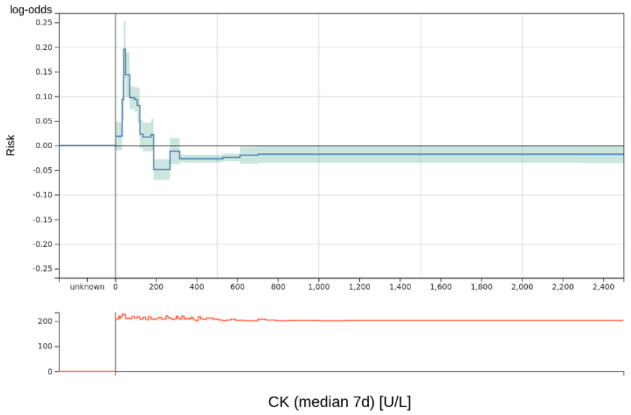

Relative importance: 1.04%

Applicable exclusion criteria: 3

Notes:

- Peak at 45 probably due to non-surgery patients (confounder). High values for example after heart surgery.
- Low risk for high values against medical knowledge.

Decision: 3

50. Lactate (max 3d) [mmol/L]

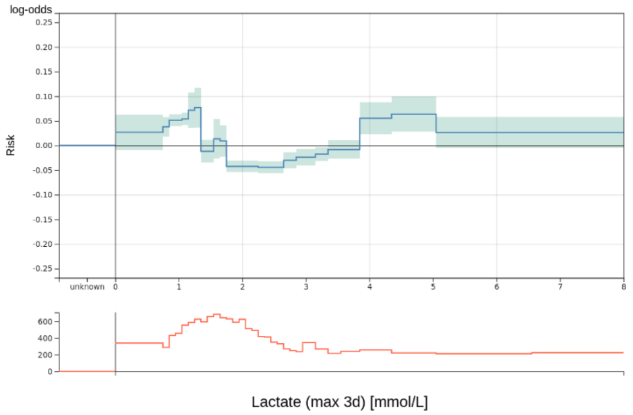

Relative importance: 1.04%

Applicable exclusion criteria: -

Notes:

- Drop only at 1.8 and not earlier probably because higher lactate values are monitored more closely.

Decision: 1

51. CK-MB (median 3d) [U/L]

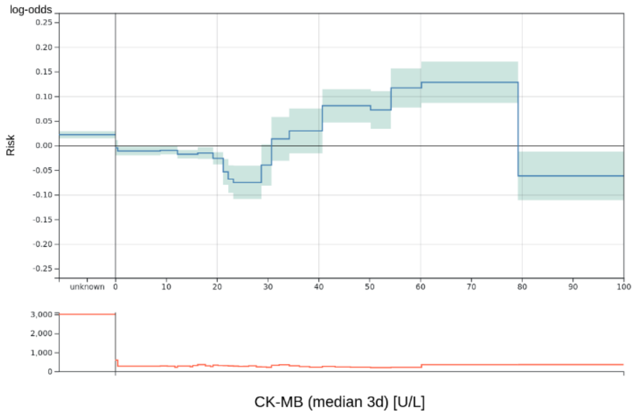

Relative importance: 1.04%

Applicable exclusion criteria: -

Notes: -

Decision: 1

52. Lactate (min 12h) [mmol/L]

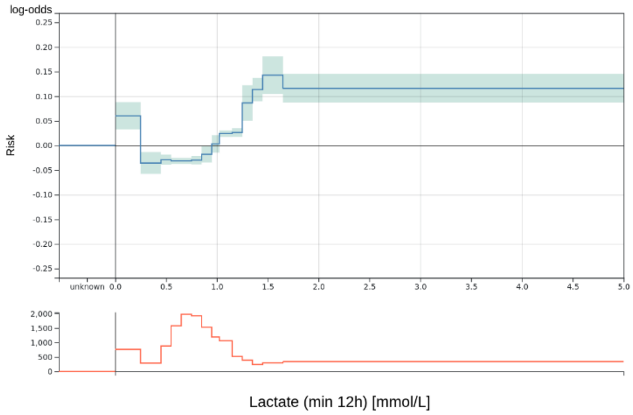

Relative importance: 1.00%

Applicable exclusion criteria: -

Notes: -

Decision: 1

53. Phosphate (max 1d) [mg/dL]

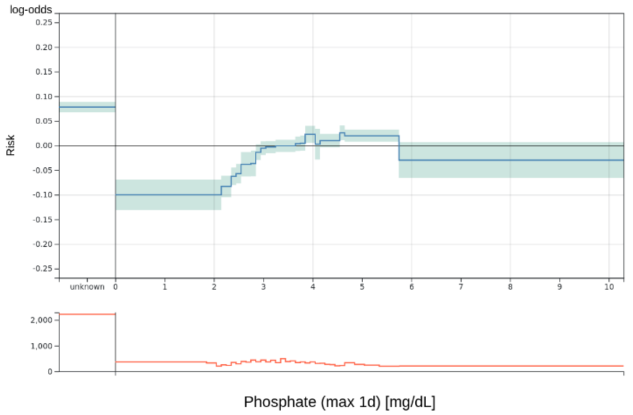

Relative importance: 1.00%

Applicable exclusion criteria: -

Notes: -

Decision: 1

54. PTT (max 7d) [s]

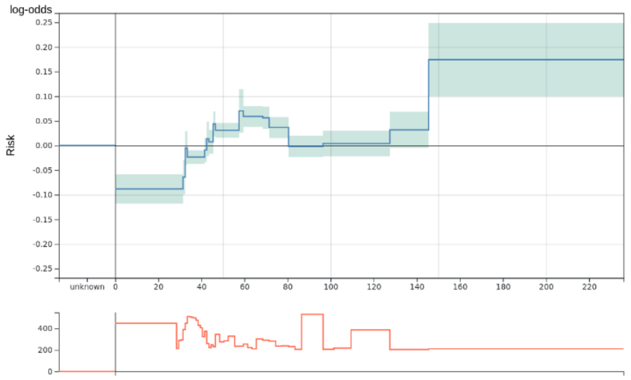

PTT (max 7d) [s]

Relative importance: 0.98%

Applicable exclusion criteria: 2

Notes:

- Practice of measuring PTT changed in 2019, which cannot be corrected easily.

Decision: 3

55. pCO2 (median 1d) [mmHg]

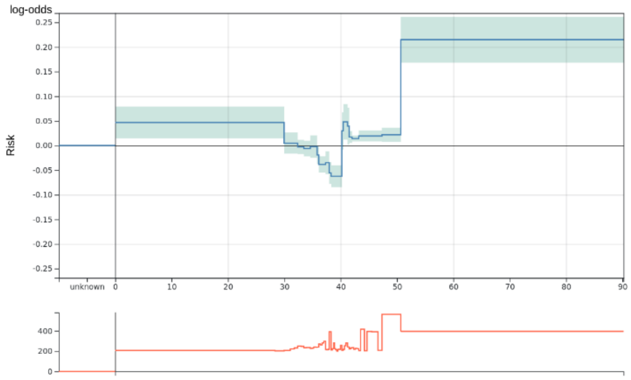

pCO2 (median 1d) [mmHg]

Relative importance: 0.98%

Applicable exclusion criteria: -

Notes: -

Decision: 1

56. BE (trend per day 3d) [mmol/L]

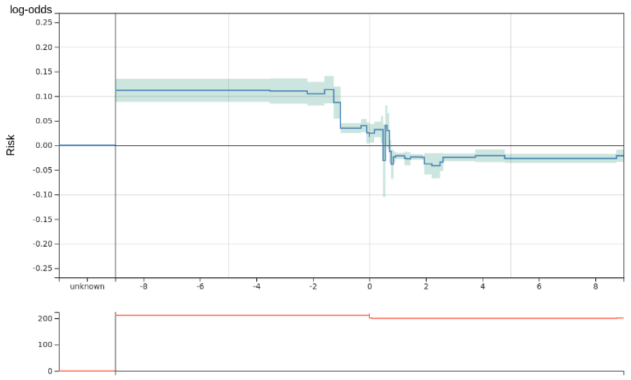

BE (trend per day 3d) [mmol/L]

Relative importance: 0.97%

Applicable exclusion criteria: -

Notes: -

Decision: 1

57. Glucose (median 3d) [mg/dL]

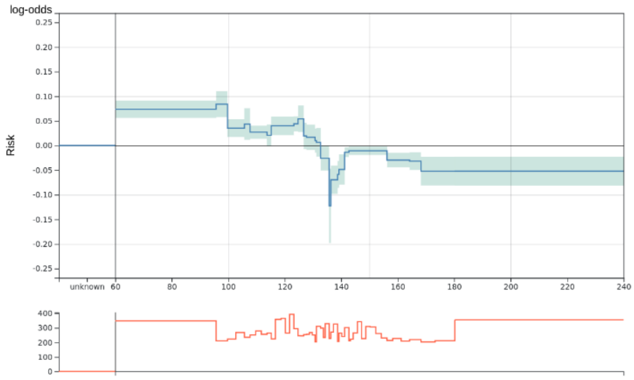

Glucose (median 3d) [mg/dL]

Relative importance: 0.97%

Applicable exclusion criteria: -

Notes: -

Decision: 1

58. BE (min 12h) [mmol/L]

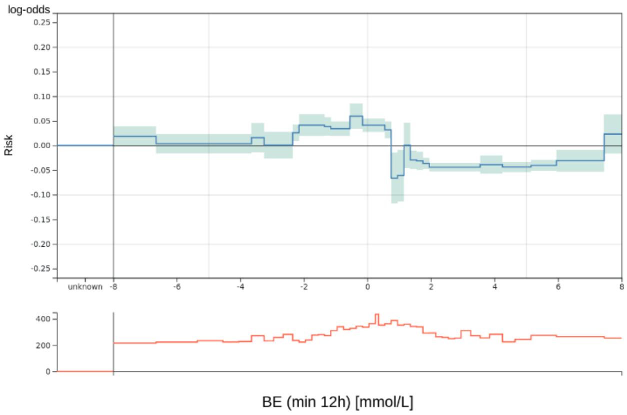

Relative importance: 0.96%

Applicable exclusion criteria: -

Notes: -

Decision: 1

59. MetHb (min 12h) [%]

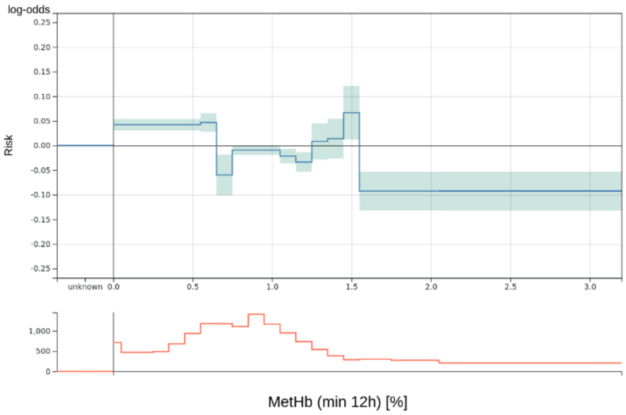

Relative importance: 0.96%

Applicable exclusion criteria: 4

Notes:

- Seldomly used in clinical practice.

Decision: 2

60. Is on automatic ventilation (days since last application per icu stay)

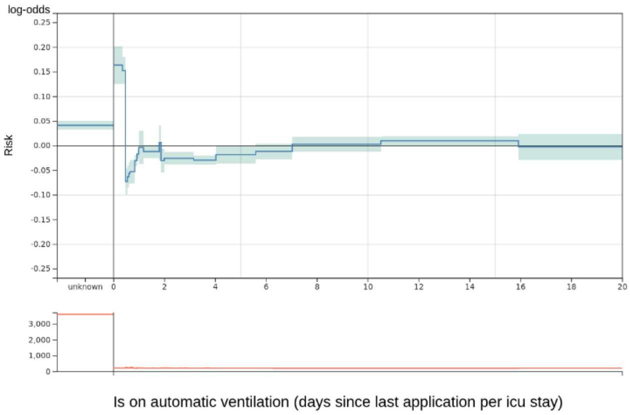

Relative importance: 0.95%

Applicable exclusion criteria: -

Notes:

- Drop at 0.5 similarly to tubus features probably because of regularly treated surgical patients.

Decision: 1

61. Body core temperature (min 4h) [°C]

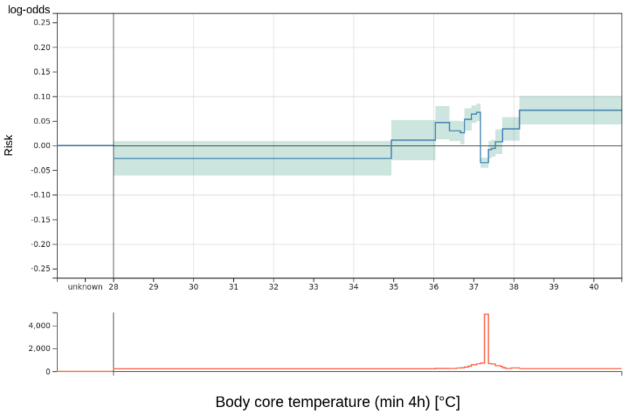

Relative importance: 0.95%

Applicable exclusion criteria: 3, 4

Notes:

- Values in normal range receive very different risk values.

Decision: 3

62. pCO2 (iqr 1d) [mmHg]

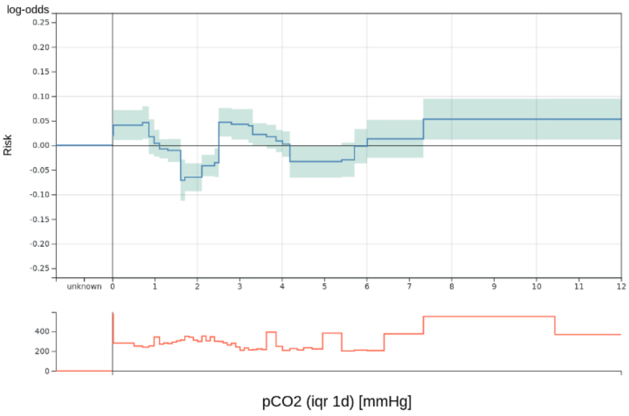

Relative importance: 0.95%

Applicable exclusion criteria: 4

Notes: -

Decision: 2

63. Sodium (median 3d) [mmol/L]

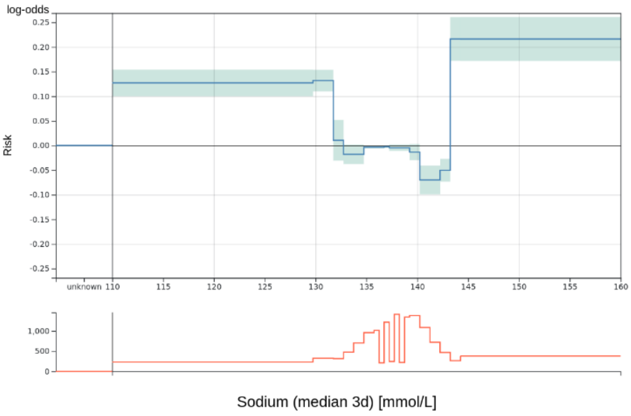

Relative importance: 0.93%

Applicable exclusion criteria: -

Notes: -

Decision: 1

64. Leucocytes (median 1d) [thousand/µL]

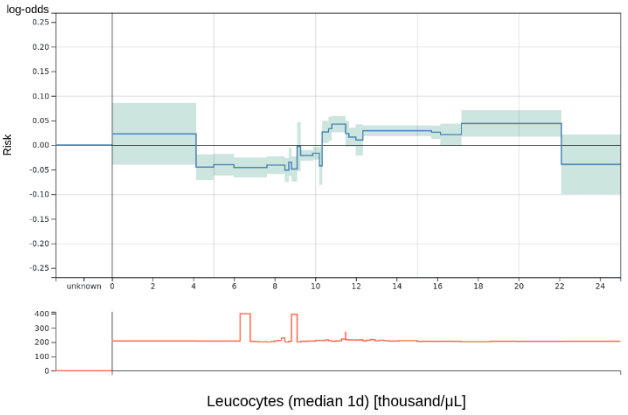

Relative importance: 0.92%

Applicable exclusion criteria: -

Notes: -

Decision: 1

65. Sodium (trend per day 3d) [mmol/L]

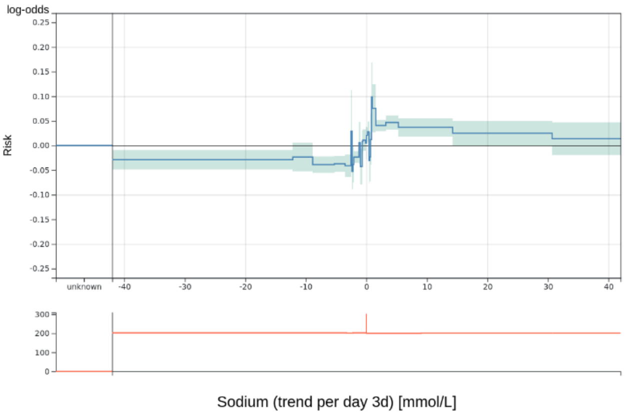

Relative importance: 0.92%

Applicable exclusion criteria: 4

Notes: -

Decision: 2

66. Procalcitonin (max 7d) [ng/mL]

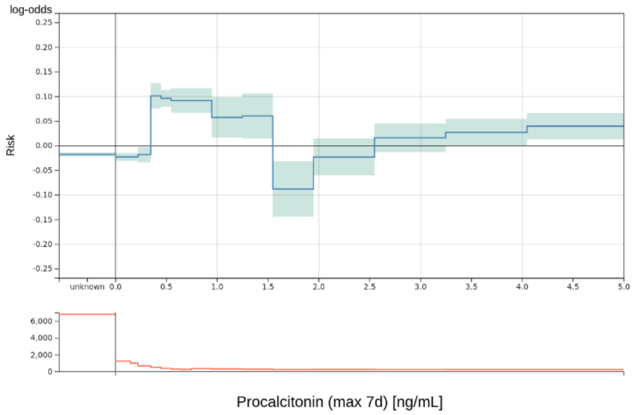

Relative importance: 0.91%

Applicable exclusion criteria: -

Notes:

- Lower risk for higher values probably because monitored more closely.

Decision: 1

67. BE (median 12h) [mmol/L]

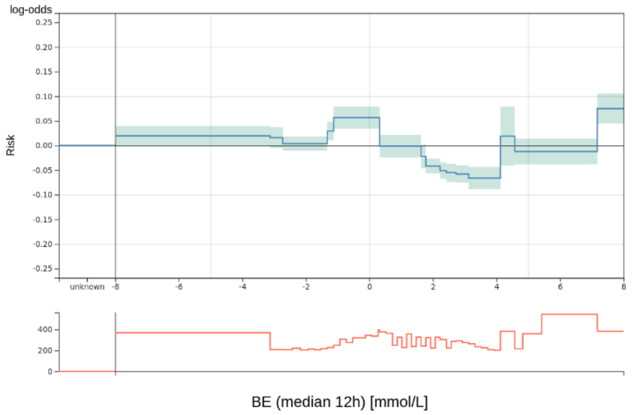

Relative importance: 0.91%

Applicable exclusion criteria: -

Notes: -

Decision: 1

68. Mean blood pressure (median 4h) [mmHg]

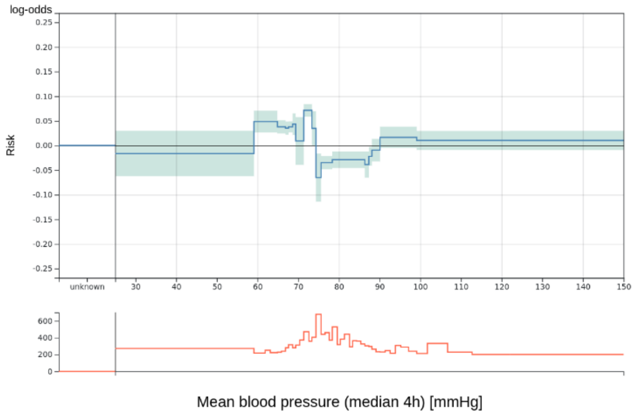

Relative importance: 0.87%

Applicable exclusion criteria: -

Notes: -

Decision: 1

69. Leucocytes (trend per day 3d) [thousand/μL]

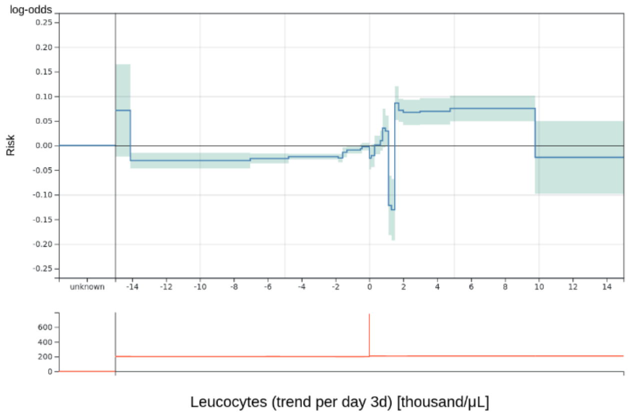

Relative importance: 0.84%

Applicable exclusion criteria: 3

Notes:

- No medical explanation for the drop at 1.2.

Decision: 3

70. pH (median 3d)

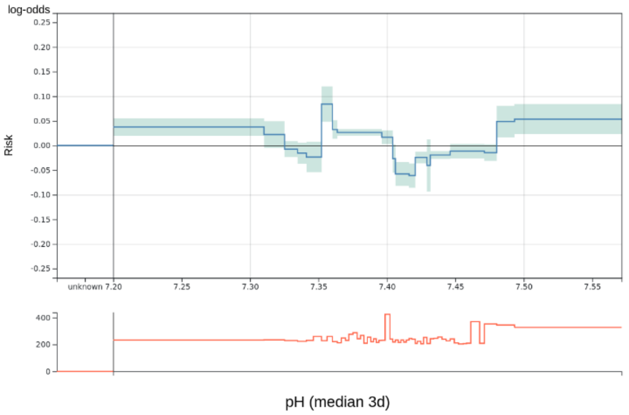

Relative importance: 0.84%

Applicable exclusion criteria: -

Notes: -

Decision: 1

71. Bilirubin total (max 7d) [mg/dL]

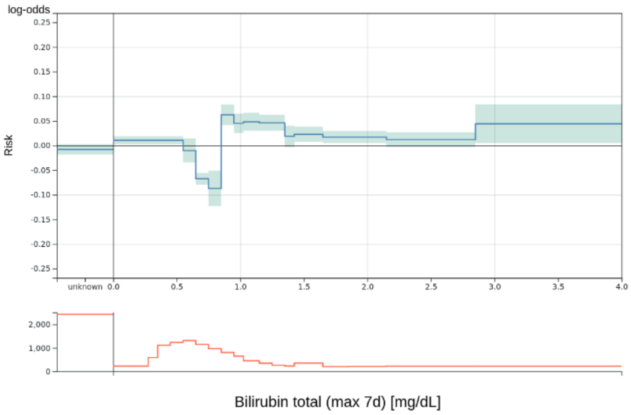

Relative importance: 0.84%

Applicable exclusion criteria: -

Notes: -

Decision: 1

72. pO2 (iqr 12h) [mmHg]

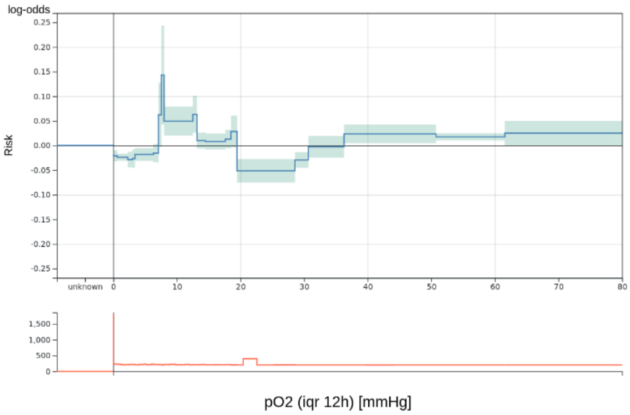

Relative importance: 0.84%

Applicable exclusion criteria: 3

Notes: -

Decision: 2

73. BE (iqr 1d) [mmol/L]

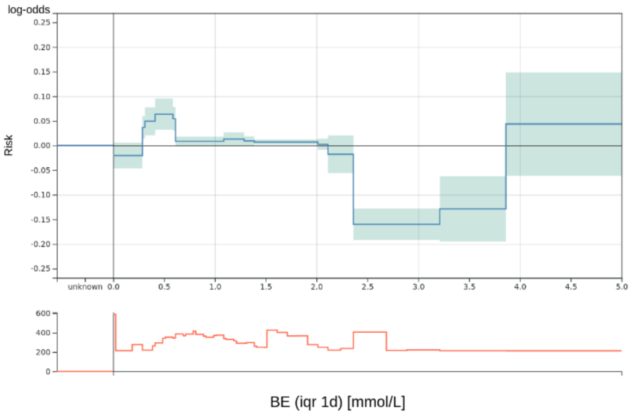

Relative importance: 0.83%

Applicable exclusion criteria: -

Notes: -

Decision: 1

74. Body core temperature (trend per day 1d) [°C]

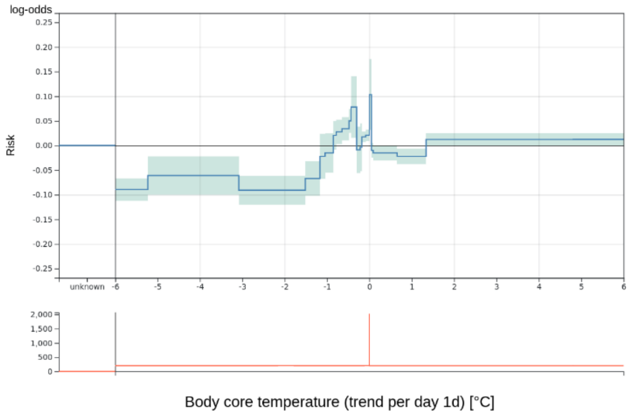

Relative importance: 0.83%

Applicable exclusion criteria: 3, 4

Notes:

- Even though difficult to interpret, no clear evidence to remove it.

Decision: 2

75. C-reactive protein (max 3d) [mg/dL]

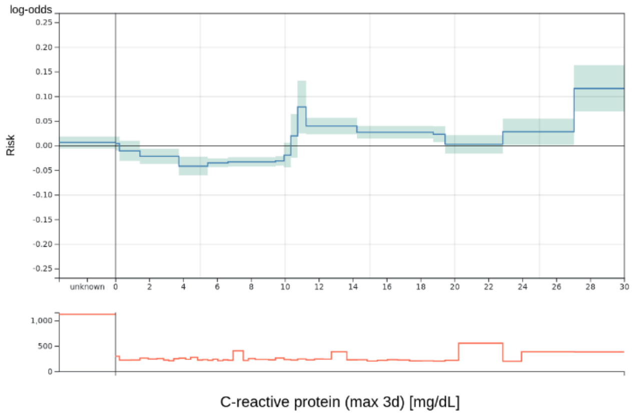

Relative importance: 0.83%

Applicable exclusion criteria: -

Notes: -

Decision: 1

76. Heart rate (min 1d) [bpm]

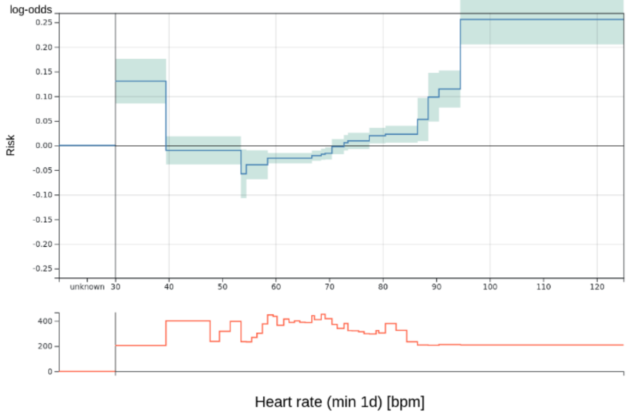

Relative importance: 0.82%

Applicable exclusion criteria: -

Notes: -

Decision: 1

77. Hematocrit (median 12h) [%]

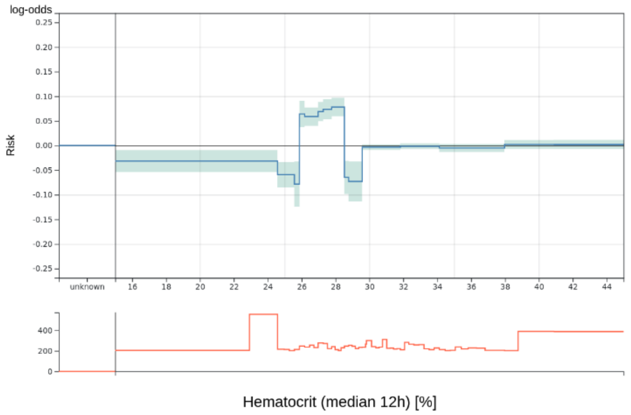

Relative importance: 0.80%

Applicable exclusion criteria: 3

Notes:

- Drops at 25 and 28 against medical knowledge but effect considered small.

Decision: 2

78. pCO2 (min 3d) [mmHg]

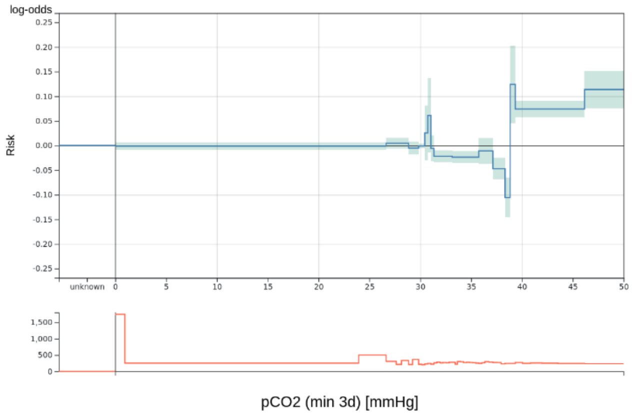

Relative importance: 0.76%

Applicable exclusion criteria: -

Notes: -

Decision: 1

79. Mean blood pressure (median 12h) [mmHg]

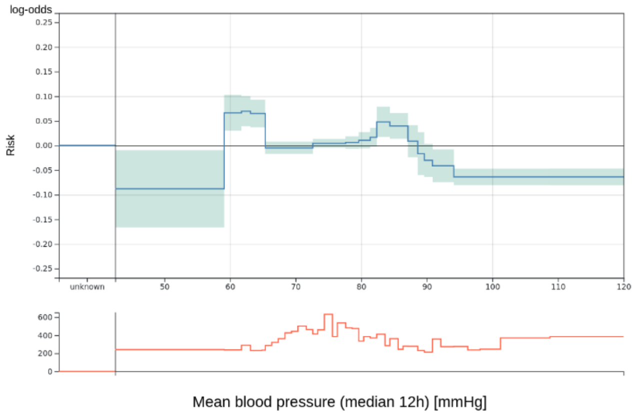

Relative importance: 0.72%

Applicable exclusion criteria: -

Notes: -

Decision: 1

80. Calcium (max 1d) [mmol/L]

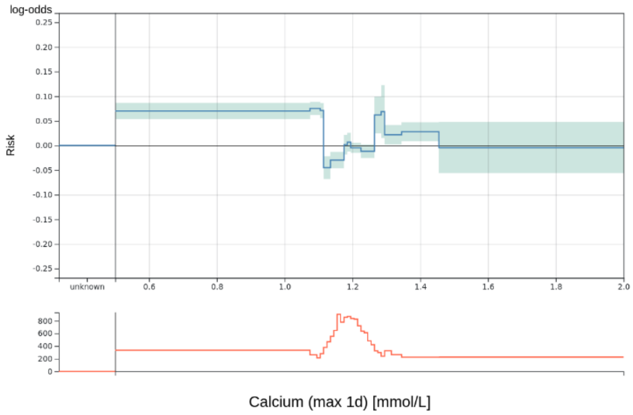

Relative importance: 0.69%

Applicable exclusion criteria: -

Notes: -

Decision: 1

81. Estimated respiratory rate (median 1d)

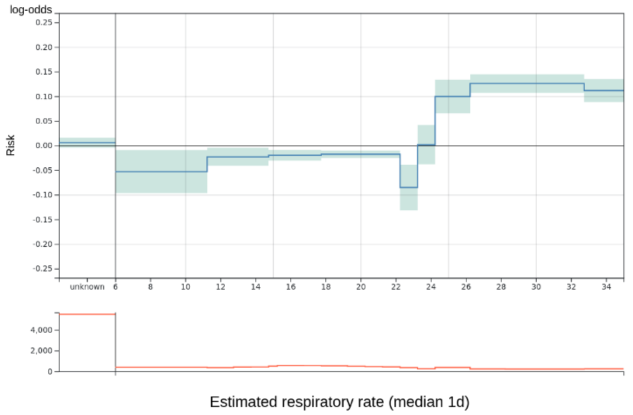

Relative importance: 0.68%

Applicable exclusion criteria: -

Notes: -

Decision: 1

82. pH (iqr 1d)

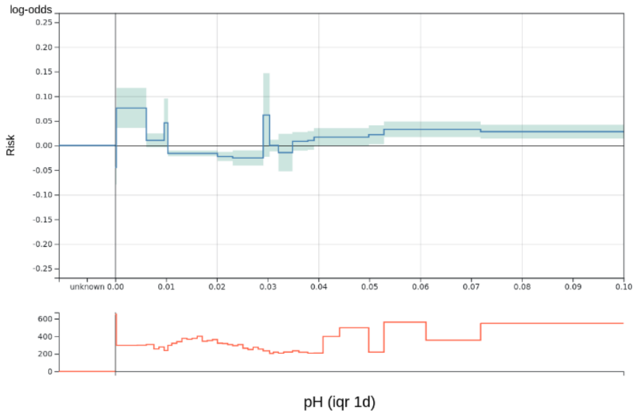

Relative importance: 0.67%

Applicable exclusion criteria: -

Notes: -

Decision: 1

83. Leucocytes (iqr 3d) [thousand/μL]

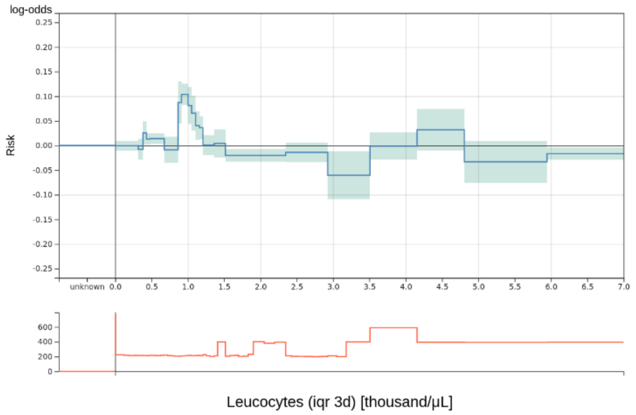

Relative importance: 0.63%

Applicable exclusion criteria: 4

Notes: -

Decision: 2

84. Heart rate (iqr 4h) [bpm]

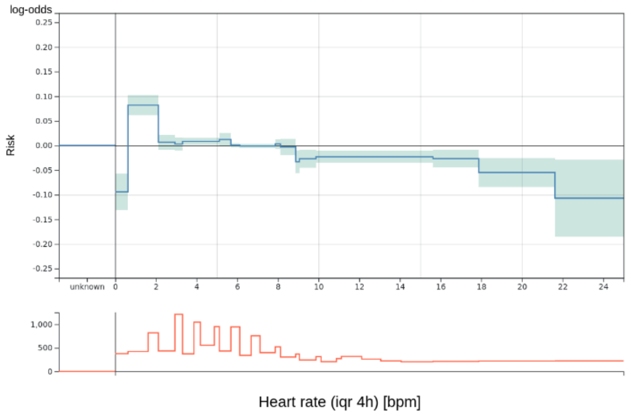

Relative importance: 0.60%

Applicable exclusion criteria: -

Notes: -

Decision: 1

85. RHb (median 12h)

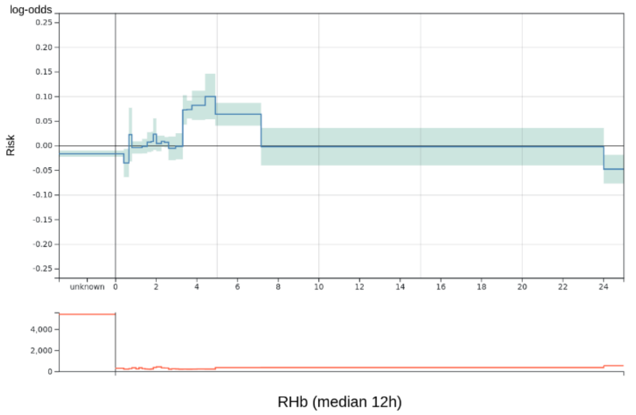

Relative importance: 0.60%

Applicable exclusion criteria: 4

Notes:

- Calculated variable without medical relevance that is only used as quality measure in clinical practice, so cannot determine its effect.

Decision: 3
